# Digital phenotyping for mental health conditions: a systematic review of implementation and application

**DOI:** 10.3389/fdgth.2026.1772744

**Published:** 2026-07-09

**Authors:** Nadia Binte Alam, Tahsinul Haque, Sanjana Subedar, Domenico Giacco, Swaran P. Singh, Sagar Jilka

**Affiliations:** 1 Warwick Medical School, University of Warwick, Coventry, United Kingdom; 2 Warwick Centre for Global Health, University of Warwick, Coventry, United Kingdom; 3 Office of Research, North South University, Dhaka, Bangladesh; 4 Coventry and Warwickshire NHS Partnership Trust, Coventry, United Kingdom

**Keywords:** digital phenotyping, mental health, methodolody, psychology, standardisation

## Abstract

**Introduction:**

Digital phenotyping (DP) has emerged as a promising approach for monitoring mental health conditions using passive and active data from personal digital devices. However, existing research is highly fragmented, with variability in study settings, device choices, data collection procedures, preprocessing pipelines, and analytical strategies. This lack of methodological consistency limits reproducibility, comparability across studies, and the translation of DP into routine clinical practice.

**Objective:**

The objective of the article is to examine and synthesise the methodological approaches used to implement DP systems for mental health conditions, including setting, device selection, data collection procedures, data storage, preprocessing workflows, feature extraction, and analytical strategies.

**Methods:**

A systematic search across seven databases (PubMed, Embase, PsycINFO, Scopus, Web of Science, ScienceDirect, and Google Scholar), including studies published till June 2025. We included primary empirical studies using smartphone or wearable-based DP in clinically diagnosed mental health populations. Study quality was assessed using the Mixed Methods Appraisal Tool (MMAT). Findings were synthesised using a narrative approach focused on implementation methodologies.

**Findings/results:**

Forty-seven studies were included, primarily conducted in high-income countries and focusing on conditions such as schizophrenia, bipolar disorder, and major depressive disorder. Across studies, wide heterogeneity was observed in digital devices used, sensing modalities, preprocessing strategies, feature definitions, and analytical techniques.

**Conclusions:**

DP demonstrates potential. However, methodological heterogeneity, inconsistent reporting, and concentration of evidence in high-income settings constrain reproducibility and clinical translation. Developing standardised implementation and reporting protocols may enhance the reliability of DP and facilitate its integration into routine mental health care.

**Systematic Review Registration:**

https://www.crd.york.ac.uk/PROSPERO/view/CRD42023406094, PROSPERO CRD42023406094.

## Introduction

Current methods for monitoring mental illnesses, primarily relying on in-person consultations, pose logistical challenges (1, 2). These clinic-based services demand frequent visits, limited by operating hours, and resource-intensive one-on-one interactions with healthcare providers, hindering widespread adoption (1). Despite the DSM-5's (3) standardised criteria, diagnosing mental disorders remains complex due to variations in symptom onset and comorbidities (1, 4). Similar limitations are reflected in the ICD-11 (5), and the longstanding debate between categorical diagnostic systems (DSM/ICD) and dimensional frameworks such as the NIMH Research Domain Criteria (RDoC) highlights the broader uncertainty regarding how best to characterise mental disorders (6–8). Cross-country differences in diagnostic practices and treatment guidelines further emphasise the lack of global consistency. Self-assessment tools offer sporadic insights and suffer from poor reliability stemming from memory limitations (1, 9). Therefore, identifying objective markers for mental illnesses is crucial.

Digital phenotyping (DP) can track everyday life outside of clinical settings to enhance the ecological validity of monitoring mental health (10). DP refers to the “moment-by-moment quantification of the individual-level human phenotype *in situ* using data from personal digital devices” (11). DP commonly relies on two types of data: passive and active (11). Passive data are collected automatically from smartphone and wearable sensors without user input, including metrics such as mobility patterns (GPS), physical activity (accelerometer), communication logs, screen interactions and sleep-wake behaviour (11). Active data, in contrast, require user participation and typically include brief self-reports, symptom scales or cognitive tasks delivered through mobile devices (11). A widely used approach for active data capture is Ecological Momentary Assessment (EMA), which involves prompting individuals to report on their current experiences in real time and in their natural environments (12). Although EMA alone is not considered DP, it is commonly integrated with passive sensing to complement behavioural signals with subjective experiences, enabling richer, contextually grounded characterisation of mental health fluctuations in daily life (11). Given the variety of methodologies and data incorporated into DP (4, 13, 14), it is essential to collate all the collected evidence into a thorough review.

DP uses smart devices like smartphones to gather data for understanding mental health status (13). Handling the vast amounts of data generated by smart devices necessitates advanced analytical techniques, often using artificial intelligence (15, 16). Machine learning is widely employed in DP research to analyse datasets and gain insights into behaviour and mental health (16). This approach has been applied in various mental disorders, including schizophrenia (17, 18), mood disorders (19, 20), and suicide prevention (21, 22), and has enabled early detection of symptoms, treatment response, and potentially avert relapse, and hospitalisation (23, 24). In recent years, the concept of DP has emerged as a powerful adjunct to traditional mental health assessment, offering continuous, real-world behavioural and physiological monitoring via smartphones and wearables (4). For example, large-scale reviews have found that spontaneous smartphone interaction data, such as screen touches, typing dynamics, and app usage can serve as proxy behavioural markers of mental health status (23). Reviews also document several distinct digital biomarkers associated with cognitive function and mood disorders (15).There is consistent evidence that mobility, phone usage, sleep and accelerometery-derived features could predict relapse or symptom exacerbation (23). Several studies have shown that changes in social communication patterns, such as reduced call frequency, altered messaging behaviour and irregular screen-on rhythms can signal worsening depressive symptoms or heightened relapse risk in mood disorders (25, 26). Wearable-derived physiological data, including heart-rate variability and autonomic arousal, have also been used to detect early signs of stress and anxiety, with machine-learning models achieving accuracies up to 78%–85% in predicting short-term symptom changes (27). In schizophrenia, deviations in geolocation entropy, reduced mobility radius and disrupted sleep–wake patterns have been shown to precede clinical relapse by several days, demonstrating the potential of passive sensing to support early warning systems (28). Collectively, this evidence shows the growing translational potential of DP to complement conventional clinical assessments and enable more proactive, data-driven models of psychiatric care.

DP generates a high volume of continuous, multi-modal sensor data (e.g., GPS, accelerometery, screen usage, keystrokes) which presents unique analytical challenges that go far beyond traditional statistical methods (23). To transform these rich data streams into usable clinical insights, machine-learning algorithms are commonly applied to handle feature extraction, identify non-linear patterns, detect anomalies, and forecast future states. For example, supervised learning methods such as support-vector machines, gradient-boosted decision trees and deep neural networks have been used to classify depressive vs. non-depressive states and to predict relapse over time (13). Moreover, systematic reviews of DP in mental health consistently highlight the prevalence of machine learning pipelines in processing sensor-derived behavioural features and stress the need for greater standardisation of these analytic workflows (29). Thus, the methodological connection between DP and machine-learning is fundamental: without machine learning, vast sensor datasets could not be effectively translated into behavioural phenotypes and predictive models that hold clinical promise.

However, current research is fragmented (11, 30), with researchers lacking a common language for describing problems, interventions, and outcomes, particularly in DP studies (10). Typically, research varies in terms of data collection, analysis and the evaluation metrics reported (10). Studies showed diversity in data collection methodologies, ranging from passive smartphone sensor data to active user inputs, which are often tailored to specific study designs rather than adhering to standard guidelines (10).

Given this diversity across studies, there's a need for a comprehensive review focusing on the implementation of DP systems. In this review, we use the term implementation to refer to the methodological processes through which DP systems are operationalised within empirical studies, including device selection, sensing modalities, data collection procedures, preprocessing workflows, feature extraction, and analytical strategies. This usage differs from the concept of implementation used in dissemination and implementation science, where the term typically refers to the adoption and integration of interventions into healthcare systems or routine clinical practice. As implementation pathways commonly occur in generalist mental health services and mixed-diagnosis settings, we adopted a broad definition of mental health conditions. This allows us to capture how DP tools are deployed across the full spectrum of clinical contexts, rather than limiting the evidence base to diagnosis-specific research. In doing so, we recognise that the value of DP lies not only in its disorder-specific predictive potential but also in its capacity to provide flexible, scalable sensing frameworks that can be adapted to diverse clinical workflows. Existing reviews primarily focus on the clinical potential of DP, with limited attention to how systems are implemented across the methodological pipeline. Consequently, reporting of implementation practices remains inconsistent. Accordingly, the aim of this review is to examine and synthesise the methodological approaches used to implement DP systems, including setting, device selection, data collection procedures, data storage, preprocessing workflows, feature extraction, and analytical strategies, with the goal of clarifying current practice and identifying areas where greater methodological consistency is needed to support future standardisation.

## Methods

This systematic review was conducted based on the Preferred Reporting Items for Systematic Reviews and Meta-analyses (PRISMA)3 guidelines and registered with PROSPERO (registration number CRD42023406094). We adopted a narrative synthesis approach to interpret and integrate the findings from the included studies. This is well suited as the objective is to map methodological diversity and synthesise complex, heterogeneous evidence rather than quantify effect sizes or evaluate clinical efficacy (14). The purpose of this narrative approach is to produce a broad structured interpretation of how DP has been implemented for serious mental disorders (SMD), while identifying areas of convergence, inconsistency, and gaps requiring further investigation.

### Search strategy

Our search terms can be found in the Supplementary File 1. The search was conducted in three phases. Firstly, an exhaustive search of databases (PubMed, Embase, PsychInfo, Scopus, Science Direct and Web of Science) was conducted on 8th June 2025 to initially screen articles. Secondly, hand searching was performed on Google Scholar and Elsevier database platform using key terms from the search strategy. The same key terms used in the database search were applied in Google Scholar, and the first 200 results (approximately the first 10 pages of results) sorted by relevance were screened. No additional filters were applied beyond restricting results to English-language publications. Lastly, manual forward and backward citation search was conducted on published reviews of DP and mental disorders.

### Study selection

#### Eligibility criteria

Our inclusion and exclusion criteria were designed to ensure that this review captured primary DP studies directly relevant to mental health while maintaining methodological coherence. We restricted inclusion to studies involving participants with clinician-diagnosed mental disorders and aged ≥14 years, as SMDs typically emerge during adolescence or early adulthood (31), with many DP studies starting recruitment from that age Using this cutoff allowed inclusion of adolescent clinical samples while excluding paediatric populations where smartphone ownership and independent device use may differ substantially. We excluded studies involving participants with non-mental-health conditions, substance-use–only populations, or participants without access to smart devices, as these would not allow us to meaningfully examine DP methodologies for psychiatric care. Likewise, we limited the review to primary empirical studies, studies were required to report original, participant-level data (observational or interventional) and excluded systematic reviews, protocols, commentaries, and conference abstracts. Systematic reviews were intentionally excluded to maintain a consistent methodological scope and avoid data redundancy. Our aim was not to conduct an umbrella review but to synthesise original evidence on how DP systems are implemented; restricting inclusion to primary studies ensures that the findings directly reflect foundational data rather than secondary interpretations. Furthermore, they are designed around their own specific and often narrower research questions, diagnostic boundaries, and methodological criteria, which differs from the broader focus of this review. Including them as study types would therefore have introduced conceptual inconsistency and the possibility of inheriting methodological biases from earlier work. However, in line with best practice for systematic reviews, we screened the reference lists of relevant reviews to ensure that no eligible primary studies were missed. Finally, studies focusing solely on online mental health tools (e.g., journaling, digital diaries, telehealth appointments) were excluded because they do not involve sensor-derived behavioural or physiological data and therefore do not meet the operational definition of DP used in this review. The studies were selected adhering to PICOS elements (32). This can be found in Table 1.

**Table 1:** Study eligibility criteria.

Criteria	Inclusion	Exclusion
Participants/population	Clinical population with diagnosed mental disorder.	History of substance-use.
	Participants aged ≥14 years Participants using/with a smart device (smartphone/smartwatch).	Health conditions unrelated to mental health. Participants aged <14 years. Participants without/unwilling to use a smart device
Interventions, exposures	Digital phenotyping data collected via smart devices.	Online mental health solutions only. Online journaling only Outcome-only digital tools (e.g., diaries, tele-appointments, self-help apps) Sensor data from non-smart or non-wearable devices.
Comparator(s)/control	N/A	N/A
Types of study	Observational or interventional studies in English.	Non-English studies. Non-primary publications (protocols, reviews, theses, commentaries, posters). Animal studies.

#### Operational definition of smart digital devices

Given the absence of universally agreed definitions of DP devices in the literature, we adopted a pragmatic but conceptually informed definition of smart digital devices for the purposes of this review. To ensure comprehensive coverage of DP methodologies, we adopted a deliberately broad operational definition of smart digital devices. This definition was guided by our search strategy, which encompassed smartphones, smartwatches, wearable devices, GPS, accelerometers, sensor data, and digital biomarkers. For the purposes of this review, smart digital devices were defined as: any consumer-grade or research-grade electronic device capable of generating passive or active sensor-based data relevant to DP, including smartphones, commercial wearables, smartwatches, and actigraphy devices when used within a DP protocol. Both commercial and research-grade wearables were included, provided they produced digitally recorded behavioural or physiological signals suitable for quantitative analysis. By adopting this broad definition of smart digital devices, we were able to reveal the significant heterogeneity in data collection methods, device types, preprocessing approaches, and analytic techniques, which is a central finding of this review.

#### Selection

All articles were uploaded on the Rayyan systematic review management program (33). Study titles and abstracts were screened and selected by three authors (NA, TH and SS) independently in Rayyan. Full-text articles of potentially eligible studies were then independently assessed by the same reviewers against the predefined inclusion and exclusion criteria. Screening at both stages was conducted blind to reviewer decisions. Reasons for exclusion were documented in Rayyan. Any discrepancies between authors were adjudicated by a fourth author (SJ). The selection process was documented in the PRISMA flow diagram. No automation tools were used to assist study selection beyond the use of Rayyan for record management.

#### Data extraction

Three authors (NA, SS and TH) independently extracted the following data from the included studies: study characteristics (e.g., location, setting), study methodology (e.g., data collection platform, pre-processing, analysis), sample characteristics (e.g., sample size, gender), data characteristics (e.g., active, passive), outcomes (e.g., findings, limitations). Each included study was reviewed in full, and relevant information was systematically recorded. Discrepancies in extracted data were resolved through discussion and consensus among the reviewers. Where necessary, extracted data were cross-checked against the original publications to ensure accuracy. No automation tools were used for data extraction.

#### Data synthesis

We carried out a narrative synthesis (34) of the included studies, as the focus of this review was the implementation and applications of DP research. We used Popay et al.'s guidance for narrative synthesis (35), focusing on the settings of the study, data points collected from each digital device, how this data was collected, stored, and analysed, and assess the robustness of synthesis. The synthesis involves organizing and describing the findings from the included studies to identify patterns or common implementation themes (35). The studies included in this review varied widely in mental disorders, device type, data collection protocols, preprocessing methods, feature extraction techniques, and analytical strategies, and did not share a common outcome measure suitable for quantitative pooling, therefore a meta-analysis was not conducted. Narrative synthesis enabled us to systematically catalogue and compare these methodological dimensions, explore patterns and relationships between methodological choices and study contexts, and map gaps and inconsistencies in the literature (14, 36, 37).

Following this approach, all studies meeting the inclusion criteria were included in a single synthesis and were not excluded or grouped based on study design, clinical population, device type, or analytical approach, as the aim was to characterise methodological diversity across DP research. No statistical transformation, data conversion, or imputation was undertaken, as quantitative synthesis was not performed; where information required for synthesis was missing or unclear, data were recorded as ‘not applicable/ N/A’ rather than inferred. Implementation characteristics and study-level variables were tabulated to support structured comparison across studies, including data modalities, devices, data collection platforms, preprocessing methods, feature extraction strategies, and analytical techniques. Methodological heterogeneity was explored descriptively by comparing these characteristics across studies, and no formal subgroup analyses, meta-regression, or sensitivity analyses were conducted.

#### Data items

The primary outcomes of interest were implementation-related characteristics of DP. These included digital devices used, data modalities collected (active/clinical and passive), data collection platforms, preprocessing and data-cleaning methods, feature extraction strategies, and analytical techniques, including statistical and machine-learning models. For each outcome domain, all results reported within individual studies that were relevant to implementation were extracted, irrespective of the specific measures, time points, or analytic approaches used. This approach was adopted to capture methodological diversity rather than prioritising a single outcome or analysis. Extracted outcomes are summarised in Tables 2–4 and Supplementary File 2.

**Table 2:** Characteristics of study.

Study ID	Authors	Year	Country	Disorder	Sample, n	Gender,n	Symptom Investigated	Settings (inpatient/outpatient etc)
1.	Abdullah et al.	2016	United States	Bipolar Disorder	9	Male,4; Female,5	Rhythmicity (consistency of daily activites and social interactions)	Outpatient
2.	Adler et al.	2020	United States	Schizophrenia Spectrum Disorders (SSD)	60	Female, 60	Early warning signs of psychotic relapse	Outpatient
3.	Camargo et al.	2025	Australia	Major Depressive Disorder	40	Male, 12;Female, 28	Depressive symptoms	Outpatient
4.	Barnett et al.	2018	United States	Schizophrenia	17	Not specified	Depression, sleep quality,psychosis, anxiety	Inpatient
5.	Bartolomeo et al.	2023	United States	Schizophrenia	44	Not specified	Negative symptoms (anhedonia, avolition, asociality)	Outpatient
6.	Baynham et al.	2025	Australia	Major Depressive Disorder	22	Male, 5;Female, 17	Depressive symptoms, positive and negative affect, stress and anxiety.	Outpatient
7.	Ben-Zeev et al.	2017	United States	Schizophrenia, schizoaffective disorder, and bipolar disorder	28	Male,15; Female,13	Delusional beliefs, suicidal ideation, substance cravings, withdrawal symptoms,…	Inpatient
8.	Ben-Zeev et al.	2016	United States	Schizophrenia and schizoaffective disorder	20	Male 16; Female 4	Behavioural and contextual patterns	Inpatient and outpatient
9.	Cella et al.	2018	United Kingdom	Schizophrenia	55	Male 31; Female 22; Unknown 2	Autonomic Dysfunction,positive symptoms and their association with…	Outpatient
10.	Cho et al.	2019	South Korea	Major depressive disorder and bipolar disorder types 1 and 2	55	Male, 28; Female 27	Mood states and episodes	Not Specified
11.	Cho et al.	2020	South Korea	Major mood disorder	163	Male, 81; Female,82	Depressive, manic, and hypomanic episodes	Outpatient
12.	Cohen et al.	2023	United States and India	Schizophrenia	132	Male, 72; Female,58	Relapse	Outpatient
13.	Cormack et al.	2019	United Kingdom	Major Depressive Disorder	30	Male,11;Female,19	Cognitive and mood symptoms	Outpatient
14.	Daniel et al.	2022	United States	Social anxiety disorder	98	Male,26;Female,72	Social anxiety symptoms and mobility patterns	Outpatient
15.	Dominiak et al.	2022	Poland	Bipolar Disorder	84	Not specified	Depressive and manic symptoms	Inpatient and outpatient
16.	Matcham et al.	2024	United Kingdom, Netherlands, Spain, Denmark, Italy, United States, Belgium	MDD	393	Males, 94 Females, 299	Depression relapse and depressive symptom severity	Outpatient
17.	Faurholt-Jepsen et al.	2016	Denmark	Bipolar Disorder	29	Male,11;Female,18	Depressive and manic symptoms	Outpatient
18.	Faurholt-Jepsen et al.	2015	Denmark	Bipolar Disorder	78	Male,26;Female,52	Depressive and manic symptoms	Outpatient
19.	Faurholt-Jepsen et al.	2022	Denmark	Bipolar disorder and unipolar disorder	67	Female (all)	Mobility patterns	Outpatient
20.	Faurholt-Jepsen et al.	2014	Denmark	Bipolar disorder	17	Male,5;Female,12	Depressive and manic symptoms, social and communication behaviors, mobility…	Outpatient
21.	Fulford et al.	2021	United States	Schizophrenia	35	Male,26;Female,9	Social interactions and mobility patterns	Outpatient
22.	Gillett et al.	2021	United Kingdom	Bipolar Disorder and Borderline Personality Disorder	55	Male,14;Female,41	Manic and depressive symptoms	Outpatient
23.	Grünerbl et al.	2015	Austria	Bipolar disorder	10	Not specified	Depressive and manic episodes	Outpatient
24.	Haines-Delmont et al.	2020	United Kingdom	mental health disorders, specifically assessing suicide risk among patients	66	Not specified	Suicide risk	Inpatient
25.	Hays et al.	2020	United States	Schizophrenia	80	Male,43;Female,37	Mood, anxiety, psychosis symptoms Sleep quality Social functioning Cognitive…	Outpatient
26.	Henson et al.	2021	United States	Schizophrenia	90	Male,49;Female,34	Relapse	Outpatient
27.	Henson et al.	2020	United States	Schizophrenia	88	Male,45;Female,43	No individual symptom	Outpatient
28.	Henson Philip et al.	2021	United States	Schizophrenia	88	Male,44;Female,38;Other 6	Cognitive functions and schizophrenia symptomatology	Outpatient
29.	Jacobson et al.	2020	United States	Social Anxiety Disorder	72	Male,35;Female,27	SAD symptom severity	Outpatient
30.	Hsu et al.	2024	Taiwan	Bipolar Disorder	181	Male, 78;Female, 103	Daily mood score, depressive and mania symptoms.	Outpatient
31.	Kalisperakis et al.	2023	Greece	Schizophrenia and bipolar disorder	35	Male,25;Female,10	Positive and negative psychopathology	Outpatient
32.	Lakhtakia et al.	2022	United States and India	Schizophrenia	60	Male,29;Female,30;Other,1	No individual symptom	Outpatient
33.	Lee et al.	2022	South Korea	Major depressive disorder, bipolar disorder I, and bipolar II disorder​	270	Male,123;Female,147	Symptoms related to mood episodes	Outpatient
34.	Lipschitz et al.	2024	United States	Bipolar Disorder	54	Not specified	Depressive, manic, and hypomanic episodes	Outpatient
35	Narkhede et al.	2022	United States	Psychotic Disorders	107	Male,35;Female,72	Negative symptoms	Outpatient
36	Naslund et al.	2016	United States	Schizophrenia spectrum disorder, major depressive disorder, and bipolar disorder​​.	34	Male,13;Female,21	No individual symptom	Outpatient
37	Niendam et al.	2018	United States	Schizophrenia spectrum disorders and mood disorders with psychotic features​	76	Male,50;Female,26	No individual symptom	Outpatient
38	Palmius et al.	2017	United Kingdom	Bipolar disorder	36	Not specified	Depressive symptoms	Outpatient
39	Pedrelli et al.	2020	United States	Major Depressive Disorder	31	Male,8;Female,23	Depressive symptoms	Outpatient
40	Place et al.	2017	United States	post-traumatic stress disorder and depression	73	Male,49;Female,24	Depression: Depressed mood most of the day, diminished interest or pleasure in…	Outpatient
41	Staples et al.	2017	United States	Schizophrenia	17	Male,15;Female,2	Sleep	Outpatient
42	Lee et al.	2024	Taiwan	Mood disorders (bipolar and MDD)	143	Male, 51 Female, 92	Depressive and manic symptoms	Outpatient
43	Tseng et al.	2022	Taiwan	Bipolar Disorder	159	Male,70;Female,89	Mood, sleep and activity	Outpatient
44	Torous et al.	2018	United States	Schizophrenia	16	Not specified	Not individual symptoms	Outpatient
45	Zulueta et al.	2018	United States	Bipolar Disorder	16	Male,8;Female,8	Mood disturbance	Outpatient
46	Čermák et al.	2023	Czech Republic	Major Depressive Disorder	10	Male,3;Female,7	No individual symptom	Outpatient
47	Yang et al.	2025	Singapore	schizophrenia spectrum disorders	99	Male, 42;Female, 57	Positive symptoms, negative symptoms, neurocognition, depression, anxiety,…	Outpatient

Summary of the studies included in the systematic review, detailing the authors, publication year, country, target mental health disorder(s), total sample size ($n$), gender distribution, specific symptom(s) investigated, and the study setting (inpatient/outpatient).

**Table 3:** Methodology.

Study ID	Data Collected		Data Collection on Platform(s)	Data Pre-processing	Data Analysis
	Active Data	Sensor Data			
1.	SRM-5	accelerometer,microphone, location and communication	MoodRhythm app	Not mentioned.	Used SVR to predict scores and Support Vector Machine (SVM) for stability classification with 10-fold cross-validation.
2.	BPRS	accelerometer, app use, call, conversation,location, screen activity, sleep, text	CrossCheck	Derived hourly features; handled missing data by imputing with '0' or feature mean value.	Core analysis used Encoder-Decoder Neural Network models (FNN AD, GRU Seq2Seq) for Anomaly Detection based on reconstruction error.
3.	EMA, MINI diagnostic interview & QIDS-16	communication, location, phone usage, sleep, physical activity	AWARE-Light smartphone sensing app (Android only); GENEActiv wrist-worn actigraphy device; EMA surveys via AWARE-Light app	No imputation applied; actigraphy features extracted using the GGIR R package; EMA scores averaged.	Used Descriptive Statistics, Pearson Correlations, and LLMs with FDR correction for multiple comparisons.
4.	PHQ-9, GAD-7, SF-36, SFS, PSQI,WSS, BASIS-24, BACS	accelerometer, GPS, screen state	Beiwe app	GPS data converted to mobility trajectories (flights/pauses); missing data imputed via weighted resampling; daily mobility and sociability features extracted.	Used Time Series Decomposition and Hotelling's T2 Test for multivariate anomaly detection, with bootstrapping for multiple comparison adjustment.
5.	EMA, SCID-5, SCID-PD, mDES	geolocation	Beiwe	Not applicable.	Calculated positivity offset/negativity bias via regression parameters; used paired t-tests, one-way ANOVAs, and Pearson correlations to assess group differences and variable relationships.
6.	DSM-V MINI, QIDS-16	Physical activity	AWARE-Light smartphone app (Android only); GENEActiv wrist-worn actigraphy device; EMA surveys via AWARE-Light app	Actigraphy data processed using GGIR; EMA scores averaged.	Used Pearson Correlations followed by MLMMs with random intercepts for participants.
7.	EMA, BDI-2, G-PTS	Activity and location	Adapted smartphones with specialized data collection software, utilizing sensors embedded in the devices	Not mentioned.	Used Nonlinear mixed-effects models for each outcome/predictor, focusing on exploratory bivariate models without adjustment for multiple comparisons.
8.	N/A	Activity and speech	Smartphones installed with study-specific software developed by the research group	Real-time processing of ambient sound for speech features (no audio recording); accelerometer data used for active/sedentary ratings; location data fused from GPS/WiFi/cellular or tracked via Bluetooth beacons.	Not specified.
9.	PANSS, Time Use Survey	EDA, blood volume, accerometer	mHealth, Empatica E4	Excluded short/incomplete recordings; EDA data processed using Ledalab for MATLAB; HRV parameters calculated using Kubios HRV.	Used Spearman rho correlations and t-tests/univariate ANOVAs to compare groups and assess associations with clinical scores, with multiple correlation adjustments.
10.	Interviews	Activity, sleep, heart rate	eMoodchart	Extracted 13 basic features and derived 117 extended features (mean, SD, gradient) over multiple days; used only complete datasets.	EmployedRandom Forest for mood prediction; used supervised learning with temporal validation and assessed feature importance.
11.	eMoodChart	Activity, sleep, heart rate	FitBit, eMoodChart and CRM app (for intervention group)	Extracted 13 basic features and derived 117 extended features (mean, SD, slope) across 3, 6, and 12 days for a 130-feature set.	Used chi-square/t-tests for baseline comparison; employed a GLM to compare episode frequency, adjusting for baseline factors.
12.	PANSS, PHQ-9, GAD-7, SF-36, SFS, PSQI, WSS and BASIS-24	Geolocation, accelerometer, and screen state	mindLAMP	Aggregated all data streams to a daily timescale.	Used Multivariate Anomaly Detection and compared it to a logistic regression model; employed Permutation Testing and supplementary PELT Changepoint Detection.
13.	SWM, CANTAB RVP, PHQ-9, UCLA-LS	Activity, heart rate	Apple Watch app, paired with participants’ iPhones	Excluded days with <100 steps or missing HR data; computed daily performance (dprime), mood, HR, and step count; no adjustments for missing data.	Used Multilevel Reliability Analysis and Longitudinal Mixed-Effects Modeling; performed Correlation Analysis to assess validity.
14.	SIAS	Geolocation	MetricWire	GPS data segmented into spatiotemporal clusters (using a 100m/300s threshold); DBSCAN used for consolidation; OpenStreetMap/Google Maps for semantic labeling.	Used Descriptive Statistics and Linear Mixed-Effects Models to analyze intervention effects on mobility outcomes (e.g., homestay, location entropy).
15.	HDRS, YMRS	Communication, speech	BDmon	Aggregated daily call/text metrics and extracted acoustic speech features; cleaned, normalized, and handled missing data. Temporally aligned behavioral data with clinical assessments and structured for mixed-effects modeling.	Used Generalized Linear Mixed-Effects Models (GLMM) for linear regression (on severity) and logistic regression (for binary classification of affective state).Conducted analyses in R with significance set at 0.05.
16.	CIDI-SF, IDS-SR	Sleep	Fitbit Charge 2 or Fitbit Charge 3 wearable devices, integrated into the RADAR-MDD	Sleep data aggregated into four-week windows (minimum 8 valid days); features were centered, standardized, and polynomial terms generated.	Used Bayesian multivariable regression models (logistic/linear) and calculated Population Attributable Fractions (PAFs).
17.	HDRS, YMRS	Activity	Bdmon	Not mentioned.	Used Generalized Linear Mixed-Effects Models (GLMM) for both linear regression (on severity scales) and logistic regression (for binary classification).
18.	HDRS,YMRS, FAST, WHOQOL-BREF, CISS, MDI, ASRM, MASS	Sleep	MONARCA system	Standardised measures, aggregated to daily means; actigraphy segmented to 1-hour windows; missing data handled via multiple imputation (MI).	Intention-to-treat analysis with linear mixed models for repeated HAMD-17 and YMRS outcomes; YMRS log-transformed for model assumptions and back-transformed for interpretation. Exploratory subgroup analyses performed by symptom profiles and baseline severity.
19.	HDRS, YMRS	Geolocation	MONARCA system	Not mentioned.	Mixed-effects regression with random intercepts and slopes; smartphone averages aligned with HDRS-17 and YMRS ratings from 17 patients (102 assessments).
20.	HDRS, YMRS	Physical and social activity	MONARCA system	Averaged smartphone data on days with HDRS-17 and YMRS assessments and used these averages for correlation analysis with clinical ratings.	Mixed-effects regression with random intercepts and slopes using averaged smartphone data aligned to HDRS-17 and YMRS assessments (17 patients, 102 ratings).
21.	QLSIR, UCLA-LS	Ethica Data application	Daily mobility metrics were derived from imputed GPS flight/pause data, speech activity quantified using rVAD with manual validation, and EMA responses standardized for analysis.	Bivariate correlations between EMA-reported social interaction/alone time and passive mobility/voice features were analyzed by group (moderate effects ≥0.30 and *p* < 0.05 noted), with additional correlations to loneliness and social functioning compared using Fisher r-to-Z, including only participants with ≥25% EMA response rate.	
22.	QIDS,BIS-11, ASRM	Communication	True Colors system	Not mentioned.	random intercept, and clinical/demographic predictors as fixed effects, with significance at *p* < 0.05. Separate classifiers for each sensor modality were fused Linear mixed-effects regression was used with communication variables as outcomes, participant ID as a random intercept, and age, diagnosis, mood state, mood symptoms, and impulsivity as fixed effects (*p* < 0.05). Separate BD and HC models accounted for gender imbalance, and unstandardized coefficients were reported with diagnosis dummy-coded for interpretation.
23.	HDRS, YMRS	Social interaction, physical motion, travel patterns	Android smartphones equipped with a custom-developed logging application	Daily features were extracted from phone-call behavior (e.g., number, duration, variability, distinct caller IDs) and from speech/voice signals capturing conversational dynamics and acoustic properties (e.g., MFCCs, ZCR, HNR, F0). These measures were derived to support pattern-recognition analysis of communication and vocal behavior.	Separate classifiers were trained for each sensor modality (phone use, audio, GPS, accelerometer) and combined using fusion techniques (weighted, AND/OR) to improve mood-state recognition and detect state changes via deviations from a default model. Performance was evaluated with cross-validation and metrics such as accuracy, precision, and recall.
24.	Interiviews, C-SSRS	Sleep, mood, steps, engagement patterns	Custom smartphone app, integrated with wearable technology like Fitbit and health data platforms like Apple Health kit	Features were extracted (step counts, sleep) and reduced via PCA.	KNN, SVM, and Random Forests predicted suicide risk, validated with k-fold cross-validation.
25.	PHQ-9, GAD-7, PSQI, BACS, PANSS, Social Functioning Scale, Short Form Health, Survey, Behavior and Symptom Identification Scale 24	N/A	mindLAMP	Data was normalized, binned into 3-day intervals, and categorized as "elevated" or "stable."	Transition probabilities between states were calculated and validated with chi-squared tests.
26.	EMA,PHQ-9, GAD-7,PANSS, CGI, Jewels Trails A and B	Mobility, sociability, screen time, sleep	mindLAMP and Beiwe	Data was normalized, features created from passive/active data (GPS, EMAs), and missing data was addressed.	Anomaly detection identified deviations from baseline; k-means clustering categorized participants.
27.	PHQ-9, GAD-7	GPS, accelerometer, screen on/off, and communication	mindLAMP and Beiwe	Data was normalized/baselined; active/passive features were extracted and clustered using k-means.	k-means clustering classified subjects; Spearman correlation with FDR correction explored feature relationships.
28.	PHQ-9, GAD-7	GPS, accelerometer, screen time	mindLAMP and Beiwe	Screen time and cognitive scores were aggregated daily; Spearman correlation with FDR correction was used for initial preparation.	Multivariate Regression and Specification Curve Analysis (SCA) explored relationships between screen time and cognition/symptoms.
29.	SIAS, DASS-21, PANAS	Accelerometer, communication	Sensus mobile app	Raw data (accelerometer, text) was cleaned, normalized, and used to extract comprehensive temporal/autoregressive features; multiple imputation handled missing data.	An XGBoost ensemble predicted social anxiety severity, evaluated via Leave-One-Out Cross-Validation (LOOCV).
30.	HDRS, YMRS, Mini diagnositc interview	Geolocation	MoodSensing smartphone app.	Data was categorized, zero-padded, and multimodal features were extracted using pretrained models (RoBERTa, wav2vec).	GRU Deep Learning model predicted scale scores, with feature importance assessed via Lasso weights and 5-fold cross-validation.
31.	IPAQ, PANSS	Accelerometer, HRA, HRV, walking activity, sleep/wake ratio	Samsung Gear S3 smartwatch	Heart rate and sensor data were cleaned, interpolated, and de-noised; key digital phenotypes (HRV, TMA) were extracted.	Linear Mixed-Effects Models estimated the relationship between smartwatch phenotypes and PANSS score, using FDR correction.
32.	PANSS, BACS,PHQ-9, GAD-7, PSQI, EMA, cognitive games	geolocation, screen-time,call/text logs, accelerometer	Samsung Galaxy M31 (android), mindLAMP 2 (iOS)	Data cleaning was performed locally; features like "home time" and "entropy" were extracted from passive (GPS) and active data.	Non-parametric tests (Kruskal–Wallis) and linear regressions explored the relationship between clinical measures and app data (engagement, GPS).
33.	eMoodChart, clinical interviews	phone use. Sleep, steps, heart rate	Fitbit & smartphones (app not mentioned)	Missing data was imputed; features were constructed from wearable data, including circadian rhythm features via Cosinor analysis, followed by feature selection.	Random Forest predicted outcomes, evaluated by AUC, with feature importance assessed using Shapley Values.
34.	PHQ-9, ASRM	Sleep, activity, heart rate	Fitbit	Missing heart rate data was imputed at the minute level using random forest imputation.	The BiMM forest (longitudinal model) was compared against six other ML algorithms using time series split cross-validation.
35	BNSS, PANSS, LOF, clinical interviews, EMA, cognitive and rewards processing tests	Geolocation, accelerometer	Embrace smartband	A comprehensive feature selection strategy was used, combining six ML methods (e.g., Boruta, RFECV) and three statistical tests.	Multiple ML algorithms (e.g., XGBoost, Random Forest) were used for classification and validated using a split-sample approach (accuracy, AUC).
36	6-WMT, weight	Step count	Fitbit Zip	Days with zero steps were coded as 'missing' due to assumed non-wear/malfunction.	Linear Regression and Penalized Functional Regression models analyzed the relationship between step count and changes in weight/fitness.
37	BPRS	GPS, communication	Ginger.io	Passive data (metadata) was cleaned/anonymized; features like step count and interaction frequencies were extracted.	Mixed-effects linear models validated smartphone data longitudinally against clinician-rated BPRS scores.
38	QIDS-SR16	Geolocation	Ginger.io	Inaccurate location data was filtered, down-sampled, imputed, and clustered; features like entropy, location variance, and home stay were extracted.	Regression models estimated symptom scores; Quadratic Discriminant Analysis (QDA) classified episodes, validated by LOPO/K-Fold cross-validation.
39	MINI, HDRS-28	EDA, peripheral skin temperature, HR, accerometer, sleep, social interactions, activity patterns, app usage	E4 Empatica, MovisensXS	Data handling for EDA, motion, and location was improved (e.g., normalization, down-sampling), and dimensionality reduction was applied.	Ensemble Boosting and Random Forest estimated HDRS scores; the Boruta algorithm identified informative features.
40	SCID	Activity, social communication, location, device interaction & information, vocal cues	E4 Empatica wristband andMovisensXS	Features were derived from the most recent week of digital trace and audio data; cluster analysis and LASSO were used for feature reduction.	Logistic regression predicted symptom likelihood using digital and audio features, validated by 10-Fold Cross-Validation (measured by AUC).
41	PSQI, PHQ-9, GAD-7, Warning Symptoms Scale and MINI (psychosis section only)	Study specific application	Sleep features were derived from accelerometer patterns (phone stationary).	Linear regression models and linear mixed models predicted PSQI scores, validated with LOOCV.	
42	EMA, YMRS, HAMD	Geolocation,	Beiwe	GPS data was filtered, down-sampled, imputed, and used to classify movement status to generate mobility features.	ANOVA compared groups; GEE models captured dynamic, time-lagged associations between GPS mobility and EMA mood.
43	MINI,HAMD, YMRS,PSQI, ASRM, DASS-21	Geolocation	Beiwe	Outliers were excluded, data sufficiency was checked, and features were created by averaging over daily, weekly, and monthly periods.	Simple and Repeated-Measures Correlation Analysis tested the temporal associations between app-derived data and clinical measures.
44	Symptoms surveys on mood, anxiety, sleep, psychosis, and medication adherence	GPS, accelerometer, call and text logs, screen on/off status, phone battery charging status	Beiwe	Data quality was defined and quantified using metrics like Daily Number of Bursts and Frequency of Pings.	Linear Mixed Models analyzed the relationship between lagged data quality and future symptom responses, corrected via Benjamini–Hochberg–Yekutieli.
45	HDRS, YMRS	Keystrokes, accelerometer	BiAffect (keyboard), Samsung Galaxy Note 4	Missing data was handled via pairwise deletion; subject-level random intercepts were used for mood ratings.	Multiple Linear Regression Models determined predictive power on mood severity (HDRS, YMRS), evaluated by R-squared and Likelihood Ratio Testing.
46	MADRS	Distance traveled, steps, calories, sleep	Smartwatch, Health Mate (app)	Digital markers were averaged over 7 days with a validity threshold; features were Log-transformed and standardized.	Multiple linear regressions (with age/phone type as covariates) analyzed the relationship between digital markers and clinical measures.
47	PANSS, BNSS, BACS,	Geolocation, touchscreen tapping speed, sociability indices	Fitbit charge 3 or 4 HOPES app	Pre-processing steps were not mentioned in the provided text.	Scatter plots and Loess regression analyzed parameter trends over time; Spearman's correlations explored relationships with MADRS scores.

**Table 4:** Study findings and limitations.

Study ID	Findings	Limitations
1.	Automated smartphone sensing is feasible for inferring the Social Rhythm Metric score in bipolar disorder, achieving reasonable generalized accuracy (RMSE 1.40) and high classification accuracy (precision 0.85, recall 0.86) for distinguishing stable and unstable states.	Small (*n* = 7) and limited sample size (euthymic state only); short 4-week duration; risks of data gaps due to inconsistent phone carriage; use of provided phones may have altered behavior; passive nature could reduce therapeutic engagement.
2.	A significant increase in detected behavioral anomalies (social, activity, and sleep patterns) was found within the 30 days leading up to a psychotic relapse.	Limited sample size (60 participants, 18 relapses) restricts generalizability across diverse patient populations.
3.	This study provides proof-of-concept for applying a deep dynamic phenotyping approach to evaluate the effects of behavioral and pharmacological interventions in individuals with severe mental illness.	Not specified in the provided text.
4.	Individuals with schizophrenia (SZ) showed a significantly reduced positivity offset, indicating diminished approach motivation in low-arousal contexts, which was linked to lower physical activity, greater sedentary behavior, and more severe avolition/anhedonia.	Relied on subjective reports for bias (lacks physiological data); accelerometry/geolocation measures still undergoing validation; limited generalizability (chronic, stable adult outpatients); no clinical comparison group; finding may be transdiagnostic.
5.	Vigorous physical activity may have protective effects on stress and positive affect in young people with MDD, while greater variability in light and moderate activity was linked to increased stress and anxiety.	Very small analytic sample (*n* = 22) limits power/generalizability; no multiple-testing correction; COVID-19 influence; lack of clinical details (treatment/severity); sedentary behavior stratification missing; no clinical intervention tested; sample skewed toward females/older youth.
6.	Self-reported delusions and substance cravings were significantly associated with an increased likelihood of engaging in various forms of violent ideation and behavior.	The limitation field is blank/Not specified in the provided text.
7.	Significant associations were found between self-reported delusions and violent ideation/behavior, and between alcohol and cigarette cravings and violent ideation/behavior.	The limitation field for this study contains a description of the study findings, not limitations (e.g., delusions/cravings associated with violence).
8.	Digital phenotyping via active (EMA) and passive methods was both safe and acceptable in a clinical youth population with moderate-to-severe MDD, but passive sensing features did not show significant associations with depressive symptoms after correction.	Accuracy depended on participants carrying the device; data gaps for outpatients indoors (no GPS) or out of network; inpatients lacked data when leaving the Bluetooth-equipped unit.
9.	The mHealth device was highly acceptable, and individuals with schizophrenia exhibited lower Heart Rate Variability (HRV) and reduced movement compared to controls, with these measures associating with positive and negative symptoms, respectively.	Medication effects were not fully isolated; small, non-diverse sample limits generalizability; short 8-hour recording period may miss data; reliance solely on device activity measurements; tobacco use was not controlled for.
10.	The developed algorithm could moderately accurately predict mood states and episodes (including no episode, depressive, manic, and hypomanic) in patients with mood disorders using machine learning and passive digital phenotypes.	Lacked intrinsic/biological assessments; variable predictive capability (focused on prognosis, not diagnosis); HAMS/LAMS scoring method may not accurately reflect mood; model performance generalizability uncertain (no external validation).
11.	The Circadian Rhythm Management (CRM) intervention group experienced substantially fewer and shorter mood episodes and showed significant positive behavioral changes (e.g., CR amplitude, steps) after receiving warning alert feedback.	Limited generalizability due to specific sample; reliance on self-reported measures introduces bias; short duration limits long-term sustainability assessment; lack of blinding could introduce Hawthorne effect or researcher bias.
12.	A significant association was demonstrated between the occurrence of statistically significant data anomalies, detected through digital phenotyping, and relapse events in schizophrenia patients.	Relapse data relied on clinical interviews (potential for unreported relapses); COVID-19 pandemic influenced behavior/generalizability (impact varied by site); data quality issues from non-adherence (e.g., ignoring reminders).
13.	The study achieved high adherence rates, found that cognitive performance improvement plateaued over time, and revealed a modest overall linear improvement in mood across the study duration.	Small sample size (*n* = 30) limits generalizability; technical issues/device discrepancies affected data completeness/reliability; inaccurate heart rate measurements suggest sensor limits; potential self-selection bias; lack of clinical validation of findings.
14.	Participants in the EMA-only condition showed an unexpected larger decrease in the proportion of time spent at home during weekday days compared to the CBM-I condition following the intervention.	Not specified in the provided text.
15.	Behavioral data from smartphones showed a significant correlation with clinically rated symptoms (HDRS and YMRS), and specific behavioral markers, such as decreased phone call activity for depression, had predictive value in distinguishing affective states.	Small final sample (*n* = 51) limits power/generalizability; Android-only platform introduced selection bias; technical app issues/nonrandom missing data (manic patients); high privacy refusal (90% refused distance tracking); exploratory design increased risk of Type I errors.
16.	Greater variability in sleep duration/midpoint and higher sleep fragmentation were linked to increased relapse risk and greater depressive symptom severity in recurrent MDD.	Potential underpowering of primary outcome due to fewer relapses identified than anticipated.
17.	Objective smartphone data (e.g., calls, screen time, cell tower movement) showed significant correlations with clinical ratings of depression and mania, and were able to distinguish between affective states.	Small sample size limits generalizability/power; short 12-week duration may miss long-term dynamics; no iOS support introduces bias; high dependence on participant compliance for data integrity.
18.	Electronic self-monitoring did not significantly affect depressive or manic symptoms overall, but the intervention group had a trend towards more sustained depressive symptoms and fewer manic symptoms in the subgroup analysis.	Lack of blinding introduced bias; specialized clinic setting limits generalizability; potential ceiling effect (mild symptoms at baseline); complex, multi-component intervention; control group confound; short duration/follow-up limits long-term conclusions.
19.	Patients with Bipolar Disorder (BD) exhibited statistically significantly lower mobility and lower location entropy across all affective states compared to those with Unipolar Depression (UD).	Small sample size limits power/generalizability; selection bias towards capable smartphone users (excluding severely affected individuals).
20.	There was a significant correlation between self-rated mood and clinically rated depressive symptoms (HDRS-17), but not with YMRS scores (mania).	Very small sample size (*n* = 17) limits power/generalizability; selection bias (iPhone users excluded); non-representation of manic symptoms; subjective nature of self-reported data.
21.	Unlike controls, mobility metrics in individuals with schizophrenia were mostly unrelated to EMA-reported social interactions, and loneliness was associated with distinct mobility patterns compared to controls.	Small sample size limits power; use of study-provided phones may affect generalization to personal use; lacked focus on the quality of social relationships; could not adequately control for confounds (e.g., age/race).
22.	Manic symptoms were positively associated with frequency and duration of phone calls and text messages, while depressive symptoms were associated with longer durations of incoming phone calls.	Small, unevenly distributed sample (*n* = 55); potential bias from using provided phones; tracked only calls/SMS (missed social media); subjective self-reported mood; frequent assessments caused fatigue; uncontrolled confounding factors; risk of Type I errors due to no multiple-testing correction.
23.	The use of multiple sensor modalities (e.g., GPS, acceleration, phone usage) was effective in recognizing and accurately detecting state changes in depressive and manic states in bipolar disorder patients.	Very small sample size (*n* = 10) limits generalizability; inconsistent sensor use/charging led to variable/incomplete data; irregular ground truth data (only every 3 weeks) relied on subjective self-assessment.
24.	Using smartphone-generated data is feasible for developing a risk algorithm for suicide risk among inpatients, with machine learning demonstrating potential for accurate prediction.	Small cohort (*n* = 66) limits generalizability/robustness; short 7-day follow-up period; model complexity may lead to overfitting.
25.	The study demonstrated that digital phenotyping can help map the temporal dynamics of symptom interactions in schizophrenia, showing that certain symptoms (e.g., anxiety, psychosis) might trigger or elevate other symptoms in future time steps.	Small sample size (especially validation set) limits reliability; only considered 3-day intervals (may miss immediate symptom interactions).
26.	Anomaly detection demonstrated high sensitivity (89%) and reasonable specificity (75%) in predicting clinical relapses.	Variability in participation/EMA frequency affected anomaly rates; smartphone data is a proxy for behavior (potential inaccuracies); lack of specific relapse dates complicates intervention timing; control group mismatch (age/race); high anomalies in controls suggests confounds; COVID-19 period lacked relapse events.
27.	Greater dysregulation in social rhythms (suggesting less routine) correlated with higher severity of self-reported depression, anxiety, psychosis, and sleep symptoms only in individuals with schizophrenia.	Data quality/missingness issues (technical, engagement) affect results; app sensitivity might be biased; sample mismatch on age/race/education introduced confounds.
28.	A considerable heterogeneity was found in the associations between screen time and various mental health symptoms, and longitudinal analysis showed a modest association between screen time metrics and cognitive performance in schizophrenia.	Daily data aggregation misses intraday fluctuations; incomplete survey completion limited generalizability; same-day data pairing missed potential lagged effects between screen time and symptoms.
29.	Smartphone sensor data successfully predicted Social Anxiety Disorder (SAD) symptom severity with moderately strong accuracy (r=0.702) and demonstrated discriminant validity from other psychological states.	Modest, homogeneous sample (undergraduate students) limits generalizability; passive data collection lacks qualitative assessment of social interactions; focused on between-person differences, not within-person fluctuations over time.
30.	Self-scale features (PHQ-9, GAD-7) were consistently the strongest predictors of mood scores, though multimodal data (including passive sensing) supported mood prediction.	Lacked external validation; labor-intensive, staff-dependent data collection; patient pool skewed toward stable patients; poor multimedia/GPS data collection; no physician contact feature in the app; lacked illness severity stratification.
31.	Increased Heart Rate Average (HRA) and decreased HRV were associated with increases in positive and negative psychopathology, respectively, suggesting heart rate metrics are significant indicators of mental health status.	Small sample size (*n* = 35) limits generalizability; specific diagnostic group inclusion; monthly data aggregation caused loss of finer temporal resolution for acute changes.
32.	The study found no significant difference between app activities or digital phenotyping data across the three study sites, but preliminary findings suggest app-based assessment correlates with standard cognitive and clinical assessments.	COVID-19 hindered recruitment/applicability; sample was young, mild-symptom FEP patients (limits extrapolation to severe/older patients); heterogeneity across sites/small size complicates conclusions; language proficiency variation was a factor.
33.	The study successfully predicted the onset of mood episode recurrences exclusively using digital phenotypes, with circadian rhythm (CR) misalignment contributing the most to the prediction of episodes.	Accuracy of episode onsets compromised by retrospective evaluation; model lacked external validation (limits generalizability); data constraints (iOS light data missing/imputed); reliance on potentially biased/inaccurate self-report/wearable data.
34.	The BiMM forest model achieved high predictive accuracy for depression (80.1%) and (hypo)mania (89.1%) using passive Fitbit data, with personalized modeling outperforming generalized approaches.	Modest sample size limits ML generalizability; no external validation; ground truth error (self-report scales); skew toward BD I; Fitbit metrics are less precise than clinical tools; high compliance bias; no clinical intervention tested.
35	Digital phenotypes derived from mobile device-captured speech can predict mood states effectively, demonstrating the utility of mobile technology for objective, continuous monitoring.	Small/specific sample limits generalizability; negative symptoms are complex (measures may not capture all aspects); dependence on self-reported measures (biases); ongoing technical challenges (need for algorithm validation).
36	Machine learning models were effective in classifying diagnostic status and specific negative symptom domains with high accuracy, and specific active and passive digital phenotyping variables were found to be highly predictive.	Small sample size/lack of diversity limits generalizability to broader populations/settings; analysis based only on engaged participants; restricted to individuals receiving community mental health services.
37	A higher average daily step count was associated with greater weight loss among participants with serious mental illness enrolled in a weight loss program.	No control group (limits impact evaluation); sample from specific clinical program (limits generalizability); technology bias (excluded non-amenable patients); app-based negative symptom assessment showed limitations (no correlation with clinician ratings).
38	The study demonstrated the feasibility and validity of using a smartphone application for symptom monitoring in early psychosis care, with high compliance and strong correlations between app and clinician-rated assessments.	Significant data gaps/compliance issues affected continuity; small sample size from a specific community setting limits generalizability; accuracy depended on mobile device performance.
39	Geographic movement patterns, as measured through mobile device data, are strongly linked to depressive states in individuals with bipolar disorder and achieved high classification accuracy.	Small sample size; minimal variability in depressive symptoms among participants limits generalizability to patients with more fluctuating symptoms.
40	Utilizing smartphones and wrist sensors to monitor patients with Major Depressive Disorder (MDD) after a clinician-rated HDRS assessment appears viable and could offer insights into fluctuations in depressive symptom severity.	High rate of data loss (e.g., lost phones) due to technical issues affected participant retention.
41	Behavioral indicators (passively collected digital trace data and vocal features) could predict clinically assessed symptoms of depression and PTSD, and adding self-reported predictors did not significantly increase model performance.	Lacked validation against definitive methods (e.g., polysomnography); significant missing accelerometer data; small sample size/observational design limits generalizability/power.
42	Smartphone-based monitoring is feasible and effective for assessing sleep in patients with schizophrenia, classifying sleep quality with high accuracy when compared to traditional in-clinic assessments (PSQI).	Small sample size limits representativeness; no Apple iOS data (potential selection bias); missing data due to hospitalization; flexible EMA times introduced potential recall bias.
43	Reduced mobility (increased time spent at home and lower location variance) was associated with worsening depressive symptoms, while greater mobility (location variance and locations visited) was linked to manic symptoms.	Limited to stable outpatients (limits generalizability); irregular/inconsistent app use affected reliability; technical issues with GPS data collection; statistical analysis method risked Type I errors.
44	The smartphone app provided valid measurements of mood and sleep which were significantly correlated with clinical scales, and positive correlations were observed between mood, sleep, and activity over time.	Very small sample size (*n* = 16) limits generalizability; variability in smartphone usage/hardware introduced confounds; issues with incomplete passive data collection (GPS/accelerometer coverage).
45	Data quality metrics (e.g., accelerometer coverage and survey timing) are significantly associated with future survey scores for various symptom domains in schizophrenia, suggesting metadata contains clinically relevant predictive information.	Limited generalizability (young, mildly ill outpatients); cross-sectional design (no causal inference); possible medication effects on HR/sleep; no multiple-testing correction (exploratory design).
46	Passive digital markers captured clinically relevant patterns, with negative symptoms showing the strongest associations, and HR during sleep standing out as the most consistent biomarker for illness severity.	Small sample size with specific characteristics (limits generalizability); use of study-issued phones restricts generalizability; less accurate prediction of mania scores due to fewer predictors/sporadic nature.
47	Changes in mobile phone usage seem to be associated with mood states in individuals with bipolar disorder, suggesting that models based on passively collected keyboard metadata could be a viable detection method.	Limited study population size constrains conclusions regarding the relationship between the digital phenotyping method and conventional clinical assessment.
	Preliminary findings indicate the effectiveness of trazodone OAD monotherapy in improving depressive symptoms and sleep quality in MDD, and that digital phenotyping has the potential to provide valuable insights into treatment response.	

For the purposes of this review, “implementation” was defined as the methodological components through which digital phenotyping systems are operationalised within empirical studies. Based on the extracted variables and the structure of the synthesis, these components included three broad domains (1): study context, including country, clinical population, sample characteristics, and clinical setting (2); the DP implementation pipeline, including digital devices and sensing modalities, data collection platforms, preprocessing approaches, feature extraction, and analytical techniques; and (3) study-level outcomes and limitations, including reported findings and methodological constraints. No assumptions were made for missing or unclear information. This framework guided both data extraction and the narrative synthesis of the included studies.

#### Quality assessment & certainty of evidence

NA, SS and TH assessed the quality of the studies using the Mixed Methods Appraisal Tool (MMAT) (38) as we included quantitative (categorised in the MMAT as ‘quantitative descriptive’, ‘quantitative randomised controlled trials’ and ‘quantitative non-randomised controlled trials’), and mixed methods studies. A fourth reviewer (SJ) independently appraised the records, and inter-rater agreement was calculated and reported.

No formal assessment of certainty or confidence in the body of evidence was undertaken, as this review focused on describing methodological implementation characteristics rather than estimating quantitative effects.

## Results

**Figure 1:**
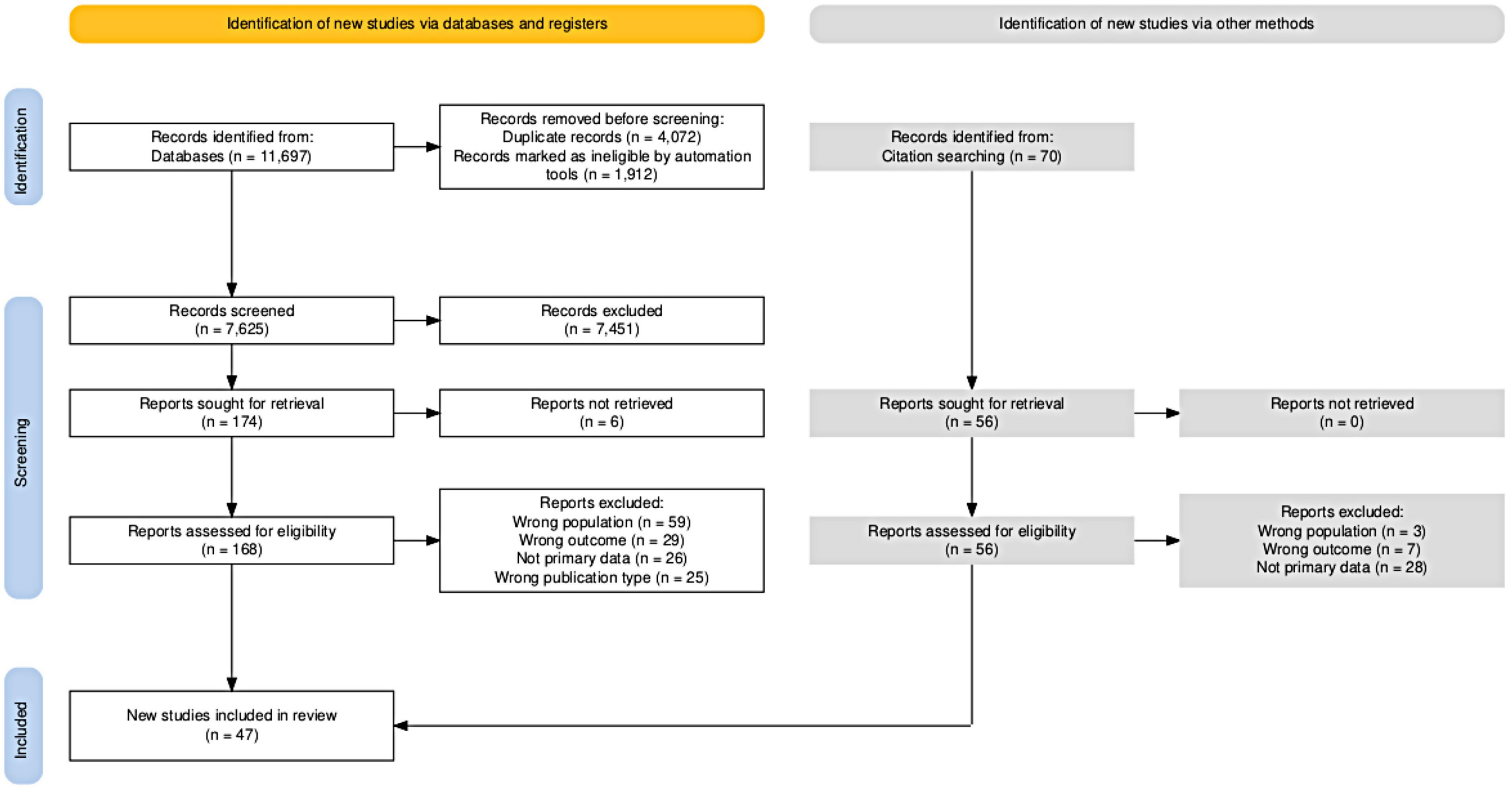
Flowchart of selection and inclusion process following the PRISMA statement (39).

We found a total of 11,697 studies of which 47 were selected for this review (Figure 1).

All records were screened independently by three reviewers at both the title/abstract and full-text stages, with disagreements resolved by a fourth reviewer. In total, 7,695 records (7,625 from database searches and 70 from citation search) were screened at the title and abstract stage. During this phase, 743 discrepancies occurred between reviewers, corresponding to an inter-rater agreement of 90.3%. At the full-text review stage, 224 articles were assessed (168 from database searches and 56 from other sources), with 31 conflicts identified, yielding an agreement rate of 86.2%. All discrepancies at both stages were resolved through consensus facilitated by the fourth reviewer. Ultimately, 47 studies met the inclusion criteria and were included in the final narrative synthesis.

### Quality assessment

Thirty-four percent (*n* = 16) of studies were quantitative descriptive (40–55) and generally had strong sampling strategies and appropriate statistical analysis methods. Fifty-nine percent (*n* = 28) studies were quantitative non-randomized control trials (QN NRCT) (26, 55–81) and 6% (*n* = 3) were quantitative randomized controlled trials (QN RCT) (82–84). Two percent (*n* = 1) of the studies were mixed methods study (28) and this study showed a strong rationale and effective integration of qualitative and quantitative components. Ten percent (*n* = 5) studies achieved a high-quality rating, effectively meeting all applicable MMAT criteria (28, 66, 72, 80, 83). Sixty-eight (*n* = 32) of the studies were rated as good quality, meeting most but not all the critical methodological criteria (26, 40, 41, 43–48, 50, 52–63, 65, 68–71, 73–79, 81, 82, 85, 86). Fourteen studies in the QN NRCT category demonstrated robust design and reporting standards (26, 56, 58, 60, 62, 65, 66, 69, 72, 74–77, 80, 81). Common strengths included clear definition of research questions and comprehensive data collection that aligns with the stated research aims. One QN RCT study demonstrated high adherence to MMAT standards, reflecting robust methodological rigor (83). Specifically, the study showed strong randomisation procedures and high levels of outcome data completeness. Twenty-one percent (*n* = 10) studies received a low-quality rating, reflecting limitations in study design, clarity of reporting, or methodological rigor (52, 55, 59, 67, 70, 71, 73, 78, 79, 84) (Table 5).

**Table 5:** MMAT appraisal tool results.

Abbreviations: Screening Questions: S1. Are there clear research questions? S2. Do the collected data allow to address the research questions? Type of Study and Questions: **QN D: Quantitative descriptive** Questions: 1. Is the sampling strategy relevant to address the research question? 2. Is the sample representative of the target population? 3. Are the measurements appropriate? 4. Is the risk of nonresponse bias low? 5. Is the statistical analysis appropriate to answer the research question? **QN RCT: Quantitative randomized controlled trials** Questions: 1. Is randomization appropriately performed? 2. Are the groups comparable at baseline? 3. Are there complete outcome data? 4. Are outcome assessors blinded to the intervention provided? 5 Did the participants adhere to the assigned intervention? **QN NRCT: Quantitative non-randomized controlled trials** Questions: 1. Are the participants representative of the target population? 2. Are measurements appropriate regarding both the outcome and intervention (or exposure)? 3. Are there complete outcome data? 4. Are the confounders accounted for in the design and analysis? 5. During the study period, is the intervention administered (or exposure occurred) as intended? **MM: Mixed methods** Questions: 1. Is there an adequate rationale for using a mixed methods design to address the research question? 2. Are the different components of the study effectively integrated to answer the research question? 3. Are the outputs of the integration of qualitative and quantitative components adequately interpreted? 4. Are divergences and inconsistencies between quantitative and qualitative results adequately addressed? 5. Do the different components of the study adhere to the quality criteria of each tradition of the methods involved?

Study ID	Title	Authors	S1	S2	Type of study	1	2	3	4	5	Results
1.	Automatic detection of social rhythms in bipolar disorder	Abdullah, Saeed; Matthews, Mark; Frank, Ellen; Doherty, Gavin; Gay, Geri; Choudhury, Tanzeem	Yes	Yes	QN NRCT	Yes	Yes	Yes	Can't tell	Yes	****
2.	Predicting Early Warning Signs of Psychotic Relapse From Passive Sensing Data: An Approach Using Encoder-Decoder Neural Networks	Adler, Daniel A; Ben-Zeev, Dror; Tseng, Vincent W-S; Kane, John M; Brian, Rachel; Campbell, Andrew T; Hauser, Marta; Scherer, Emily A; Choudhury, Tanzeem	Yes	Yes	QN NRCT	Yes	Yes	Can't tell	Can't tell	Yes	***
3.	SmartSense-D: A safety, feasibility, and acceptability pilot study of digital phenotyping in young people with major depressive disorder	Andres Camargo, Scott D Tagliaferri, Lianne Schmaal, Tianyi Zhang, Zamantha Munoz, Pemma Davies, Mario Alvarez-Jimenez, Niels van Berkel, Vassilis Kostakos, Lianne Schmaal	Yes	Yes	QN NRCT	Yes	Yes	Can't tell	Yes	Yes	****
4.	Relapse Prediction in Schizophrenia through Digital Phenotyping	Barnett, Ian; Torous, John; Staples, Patrick; Sandoval, Luis; Keshavan, Matcheri; Onnela, Jukka-Pekka	Yes	Yes	MM	Yes	Yes	Yes	Yes	Yes	*****
5.	The Positivity Offset Theory of Anhedonia in Schizophrenia: Evidence for a Deficit in Daily Life using Digital Phenotyping	Lisa A. Bartolomeo, Ian M. Raugh, Gregory P. Strauss	Yes	Yes	QN NRCT	Can't tell	Yes	No	Yes	Yes	***
6.	The Dynamic Association Between Physical Activity and Psychological Symptoms in Young People With Major Depressive Disorder: An Active and Passive Sensing Longitudinal Cohort Study	Rosalind Baynham, Andres Camargo, Simon D'Alfonso, Tianyi Zhang, Zamantha Munoz, Pemma Davies, Mario Alvarez-Jimenez, Niels van Berkel, Vassilis Kostakos, Lianne Schmaal, Scott D Tagliaferri	Yes	Yes	QN NRCT	Yes	Yes	Can't tell	Yes	Yes	****
7.	Use of Multimodal Technology to Identify Digital Correlates of Violence Among Inpatients With Serious Mental Illness: A Pilot Study	Ben-Zeev, Dror; Scherer, Emily A.; Brian, Rachel M.; Mistler, Lisa A.; Campbell, Andrew T.; Wang, Rui	Yes	Yes	QN NRCT	Yes	Yes	Can't tell	No	Yes	***
8.	Mobile Behavioral Sensing for Outpatients and Inpatients With Schizophrenia	Ben-Zeev, Dror; Wang, Rui; Abdullah, Saeed; Brian, Rachel; Scherer, Emily A; Mistler, Lisa A; Hauser, Marta; Kane, John M; Campbell, Andrew; Choudhury, Tanzeem	Yes	Yes	QN D	Yes	Yes	Yes	Can't tell	Can't tell	***
9.	Using wearable technology to detect the autonomic signature of illness severity in schizophrenia	Cella Matteo a, Okruszek Łukasz, Lawrence Megan a, Zarlenga Valerio a, He Zhimin a, Wykes Til	Yes	Yes	QN D	Yes	Can't tell	Yes	Can't tell	Yes	***
10.	Mood Prediction of Patients With Mood Disorders by Machine Learning Using Passive Digital Phenotypes Based on the Circadian Rhythm: Prospective Observational Cohort Study	Cho, Chul-Hyun; Lee, Taek; Kim, Min-Gwan; In, Hoh Peter; Kim, Leen; Lee, Heon-Jeong	Yes	Yes	QN NRCT	Yes	Yes	Yes	Can't tell	Yes	****
11.	Effectiveness of a Smartphone App With a Wearable Activity Tracker in Preventing the Recurrence of Mood Disorders: Prospective Case-Control Study	Cho, Chul-Hyun; Lee, Taek; Lee, Jung-Been; Seo, Ju Yeon; Jee, Hee-Jung; Son, Serhim; An, Hyonggin; Kim, Leen; Lee, Heon-Jeon	Yes	Yes	QN NRCT	Yes	Yes	Can't tell	Can't tell	Yes	***
12.	Relapse prediction in schizophrenia with smartphone digital phenotyping during COVID-19: a prospective, three-site, two-country, longitudinal study	Cohen, Asher; Naslund, John A.; Chang, Sarah; Nagendra, Srilakshmi; Bhan, Anant; Rozatkar, Abhijit; Thirthalli, Jagadisha; Bondre, Ameya; Tugnawat, Deepak; Reddy, Preethi V.; Dutt, Siddharth; Choudhary, Soumya; Chand, Prabhat Kumar; Patel, Vikram; Keshavan, Matcheri; Joshi, Devayani; Mehta, Urvakhsh Meherwan; Torous, John	Yes	Yes	QN D	Yes	Yes	Yes	Can't tell	Yes	****
13.	Wearable Technology for High-Frequency Cognitive and Mood Assessment in Major Depressive Disorder: Longitudinal Observational Study	Cormack, Francesca; McCue, Maggie; Taptiklis, Nick; Skirrow, Caroline; Glazer, Emilie; Panagopoulos, Elli; van Schaik, Tempest A; Fehnert, Ben; King, James; Barnett, Jennifer H	Yes	Yes	QN D	Yes	Can't tell	Yes	Can't tell	Yes	***
14.	Cognitive bias modification for threat interpretations: using passive Mobile Sensing to detect intervention effects in daily life	Katharine E. Daniel, Sanjana Mendu, Anna Baglione, Lihua Cai, Bethany A. Teachman, Laura E. Barnes, and Mehdi Boukhechba.	Yes	Yes	QN RCT	Yes	Yes	Can't tell	Can't tell	Yes	***
15.	Behavioral and Self-reported Data Collected From Smartphones for the Assessment of Depressive and Manic Symptoms in Patients With Bipolar Disorder: Prospective Observational Study	Dominiak, Monika; Kaczmarek-Majer, Katarzyna; Antosik-Wójcińska, Anna Z; Opara, Karol R; Olwert, Anna; Radziszewska, Weronika; Hryniewicz, Olgierd; Święcicki, Łukasz; Wojnar, Marcin; Mierzejewski, Paweł	Yes	Yes	QN D	Yes	Can't tell	Yes	Can't tell	Yes	***
16.	The relationship between wearable-derived sleep features and relapse in Major Depressive Disorder	F. Matcham, E. Carr, N. Meyer, K.M. White, C. Oetzmann, D. Leightley, F. Lamers, S. Siddi, N. Cummins, P. Annas, G. de Girolamo, J.M. Haro, G. Lavelle, Q. Li, F. Lombardini, D.C. Mohr, V.A. Narayan, B.W.H.J. Penninx, M. Coromina, G. Riquelme Alacid, S.K. Simblett, R. Nica, T. Wykes, J.C. Brasen, I. Myin-Germeys, R.J.B. Dobson, A.A. Folarin, Y. Ranjan, Z. Rashid, J. Dineley, S. Vairavan, M. Hotopf, on behalf of the RADAR-CNS consortium	Yes	Yes	QN NRCT	Can't tell	Yes	Can't tell	Yes	Yes	***
17.	Behavioral activities collected through smartphones and the association with illness activity in bipolar disorder	Faurholt-Jepsen, Maria; Vinberg, Maj; Frost, Mads; Debel, Sune; Margrethe Christensen, Ellen; Bardram, Jakob E.; Kessing, Lars Vedel	Yes	Yes	QN NRCT	Yes	Yes	Can't tell	Yes	Yes	****
18.	Daily electronic self-monitoring in bipolar disorder using smartphones – the MONARCA I trial: a randomized, placebo-controlled, single-blind, parallel group trial	Faurholt-Jepsen, M.; Frost, M.; Ritz, C.; Christensen, E. M.; Jacoby, A. S.; Mikkelsen, R. L.; Knorr, U.; Bardram, J. E.; Vinberg, M.; Kessing, L. V	Yes	Yes	QN RCT	Yes	Yes	Yes	Yes	Yes	*****
19.	Differences in mobility patterns according to machine learning models in patients with bipolar disorder and patients with unipolar disorder	Faurholt-Jepsen, Maria; Busk, Jonas; Rohani, Darius Adam; Frost, Mads; Tønning, Morten Lindberg; Bardram, Jakob Eyvind; Kessing, Lars Vede	Yes	Yes	QN RCT	Yes	Can't tell	No	Can't tell	Yes	**
20.	Smartphone data as objective measures of bipolar disorder symptoms	Faurholt-Jepsen, Maria; Frost, Mads; Vinberg, Maj; Christensen, Ellen Margrethe; Bardram, Jakob E.; Kessing, Lars Vedel	Yes	Yes	QN NRCT	Yes	Yes	Yes	Yes	Yes	*****
21.	Smartphone sensing of social interactions in people with and without schizophrenia	Fulford, Daniel; Mote, Jasmine; Gonzalez, Rachel; Abplanalp, Samuel; Zhang, Yuting; Luckenbaugh, Jarrod; Onnela, Jukka-Pekka; Busso, Carlos; Gard, David E.	Yes	Yes	QN D	Yes	Yes	Yes	Can't tell	Yes	****
22.	Digital Communication Biomarkers of Mood and Diagnosis in Borderline Personality Disorder, Bipolar Disorder, and Healthy Control Populations	Gillett, George; McGowan, Niall M.; Palmius, Niclas; Bilderbeck, Amy C.; Goodwin, Guy M.; Saunders, Kate E. A	Yes	Yes	QN NRCT	Can't tell	Yes	No	Can't tell	Can't tell	*
23.	Smartphone-Based Recognition of States and State Changes in Bipolar Disorder Patients	Grünerbl, Agnes; Muaremi, Amir; Osmani, Venet; Bahle, Gernot; Ohler, Stefan; Tröster, Gerhard; Mayora, Oscar; Haring, Christian; Lukowicz, Paul	Yes	Yes	QN D	Yes	Yes	Yes	Can't tell	Yes	****
24.	Testing Suicide Risk Prediction Algorithms Using Phone Measurements With Patients in Acute Mental Health Settings: Feasibility Study	Haines-Delmont, Alina; Chahal, Gurdit; Bruen, Ashley Jane; Wall, Abbie; Khan, Christina Tara; Sadashiv, Ramesh; Fearnley, David	Yes	Yes	QN NRCT	Yes	Yes	Can't tell	Can't tell	Yes	***
25.	Deriving symptom networks from digital phenotyping data in serious mental illness	Hays, Ryan; Keshavan, Matcheri; Wisniewski, Hannah; Torous, John	Yes	Yes	QN D	Yes	Yes	Yes	Can't tell	Yes	****
26.	Anomaly detection to predict relapse risk in schizophrenia	Henson, Philip; D'Mello, Ryan; Vaidyam, Aditya; Keshavan, Matcheri; Torous John	Yes	Yes	QN D	Yes	Yes	Yes	Can't tell	Yes	****
27.	Towards clinically actionable digital phenotyping targets in schizophrenia	Henson, Philip; Barnett, Ian; Keshavan, Matcheri; Torous, John	Yes	Yes	QN NRCT	Yes	Yes	Can't tell	Yes	Yes	****
28.	Investigating Associations Between Screen Time and Symptomatology in Individuals With Serious Mental Illness: Longitudinal Observational Study	Henson Philip; Rodriguez-Villa Elena; Torous John	Yes	Yes	QN D	Yes	Yes	Yes	No	Yes	****
29.	Digital Biomarkers of Social Anxiety Severity: Digital Phenotyping Using Passive Smartphone Sensors	Jacobson, Nicholas C.; Summers, Berta; Wilhelm, Sabine	Yes	Yes	QN D	Yes	Yes	Yes	Can't tell	Yes	****
30.	MoodSensing: A smartphone app for digital phenotyping and assessment of bipolar disorder	Jia-Hao Hsu, Chung-Hsien Wu, Esther Ching-Lan Lin, Po-See Chen	Yes	Yes	QN NRCT	Yes	Yes	Can't tell	No	Yes	***
31.	Smartwatch digital phenotypes predict positive and negative symptom variation in a longitudinal monitoring study of patients with psychotic disorders	Kalisperakis, Emmanouil; Karantinos, Thomas; Lazaridi, Marina; Garyfalli, Vasiliki; Filntisis, Panagiotis P.; Zlatintsi, Athanasia; Efthymiou, Niki; Mantas, Asimakis; Mantonakis, Leonidas; Mougiakos, Theodoros; Maglogiannis, Ilias; Tsanakas, Panayotis; Maragos, Petros; Smyrnis, Nikolaos	Yes	Yes	QN NRCT	Yes	Yes	Can't tell	Can't tell	Yes	***
32.	Smartphone digital phenotyping, surveys, and cognitive assessments for global mental health: Initial data and clinical correlations from an international first episode psychosis study	Lakhtakia, Tanvi; Bondre, Ameya; Chand, Prabhat Kumar; Chaturvedi, Nirmal; Choudhary, Soumya; Currey, Danielle; Dutt, Siddharth; Khan, Azaz; Kumar, Mohit; Gupta, Snehil; Nagendra, Srilakshmi; Reddy, Preethi V.; Rozatkar, Abhijit; Scheuer, Luke; Sen, Yogendra; Shrivastava, Ritu; Singh, Rahul; Thirthalli, Jagadisha; Tugnawat, Deepak Kumar; Bhan, Anant; Naslund, John A.; Patel, Vikram; Keshavan, Matcheri; Mehta, Urvakhsh Meherwan; Torous, John	Yes	Yes	QN D	Can't tell	Yes	Yes	Can't tell	Yes	***
33.	Prediction of impending mood episode recurrence using real-time digital phenotypes in major depression and bipolar disorders in South Korea: a prospective nationwide cohort study	Heon-Jeong Lee, Chul-Hyun Cho, Taek Lee, Jaegwon Jeong, Ji Won Yeom, Sojeong Kim, Sehyun Jeon, Ju Yeon Seo, Eunsoo Moon, Ji Hyun Baek, Dong Yeon Park, Se Joo Kim, Tae Hyon Ha, Boseok Cha, Hee-Ju Kang, Yong-Min Ahn, Yujin Lee, Jung-Been Lee, Leen Kim	Yes	Yes	QN NRCT	Yes	Yes	Yes	Yes	Yes	*****
34.	Digital phenotyping in bipolar disorder: Using longitudinal Fitbit data and personalized machine learning to predict mood symptomatology	Jessica M Lipschitz, Sidian Lin, Soroush Saghafian, Chelsea K Pike, Katherine E Burdick	Yes	Yes	QN NRCT	Can't tell	Yes	Yes	Can't tell	Yes	***
35.	Digital Phenotyping in Bipolar Disorder	Laura Orsolini, Michele Fiorani and Umberto Volpe	Yes	Yes	QN NRCT	Yes	Yes	Yes	No	Yes	****
36.	Machine Learning Identifies Digital Phenotyping Measures Most Relevant to Negative Symptoms in Psychotic Disorders: Implications for Clinical Trials	Narkhede, Sayli M.; Luther, Lauren; Raugh, Ian M.; Knippenberg, Anna R.; Esfahlani, Farnaz Zamani; Sayama, Hiroki; Cohen, Alex S.; Kirkpatrick, Brian; Strauss, Gregory P.	Yes	Yes	QN D	Yes	Yes	Yes	Can't tell	Yes	****
37.	Wearable devices and mobile technologies for supporting behavioral weight loss among people with serious mental illness	Naslund John A.; Aschbrenner Kelly A.; Scherer Emily A.; McHugo Gregory J.; Marsch Lisa A.; Bartels Stephen J.	Yes	Yes	QN NRCT	Yes	Yes	Yes	Can't tell	Yes	****
38.	Enhancing early psychosis treatment using smartphone technology: A longitudinal feasibility and validity study	Niendam, Tara A.; Tully, Laura M.; Iosif, Ana-Maria; Kumar, Divya; Nye, Kathleen E.; Denton, Jennifer C.; Zakskorn, Lauren N.; Fedechko, Taylor L.; Pierce, Katherine M.	Yes	Yes	QN D	Yes	No	Yes	No	Yes	***
39.	Detecting Bipolar Depression From Geographic Location Data	Palmius, N.; Tsanas, A.; Saunders, K. E. A.; Bilderbeck, A. C.; Geddes, J. R.; Goodwin, G. M.; De Vos, M.	Yes	Yes	QN NRCT	Yes	Yes	Can't tell	Yes	Yes	****
40.	Monitoring Changes in Depression Severity Using Wearable and Mobile Sensors	Pedrelli, Paola; Fedor, Szymon; Ghandeharioun, Asma; Howe, Esther; Ionescu, Dawn F; Bhathena, Darian; Fisher, Lauren B; Cusin, Christina; Nyer, Maren; Yeung, Albert; Sangermano, Lisa; Mischoulon, David; Alpert, Jonathan E; Picard, Rosaline W	Yes	Yes	QN NRCT	Yes	Yes	Yes	Yes	Can't tell	****
41.	Behavioral Indicators on a Mobile Sensing Platform Predict Clinically Validated Psychiatric Symptoms of Mood and Anxiety Disorders	Place, Skyler; Blanch-Hartigan, Danielle; Rubin, Channah; Gorrostieta, Cristina; Mead, Caroline; Kane, John; Marx, Brian P; Feast, Joshua; Deckersbach, Thilo; Alex “Sandy” Pentland; Nierenberg, Andrew; Azarbayejani, Ali	Yes	Yes	QN NRCT	Yes	Yes	Can't tell	Yes	Yes	****
42.	A comparison of passive and active estimates of sleep in a cohort with schizophrenia	Staples, Patrick; Torous, John; Barnett, Ian; Carlson, Kevin; Sandoval, Luis; Keshavan, Matcheri; Onnela, Jukka Pekka	Yes	Yes	QN D	Yes	Can't tell	Yes	Can't tell	Yes	***
43.	Dynamic Bidirectional Associations Between Global Positioning System Mobility and Ecological Momentary Assessment of Mood Symptoms in Mood Disorders: Prospective Cohort Study	Ting Yi Lee; Ching Hsuan Chen; I Ming Chen; Hsi Chung Chen; Chih Min Liu; Shu I Wu; Chuhsing Kate Hsiao; Po Hsiu Kuo	Yes	Yes	QN NRCT	Can't tell	Yes	Can't tell	Yes	Yes	***
44.	Associations among smartphone app-based measurements of mood, sleep and activity in bipolar disorder	Tseng, Yu-Ching; Lin, Esther Ching-Lan; Wu, Chung Hsien; Huang, Huei-Lin; Chen, Po See	Yes	Yes	QN NRCT	Yes	Yes	Can't tell	Can't tell	Yes	***
45.	Characterizing the clinical relevance of digital phenotyping data quality with applications to a cohort with schizophrenia	Torous, John; Staples, Patrick; Barnett, Ian; Sandoval, Luis R; Keshavan, Matcheri; Onnela, Jukka-Pekka	Yes	Yes	QN D	Yes	Can't tell	Yes	Can't tell	Yes	***
46.	Predicting Mood Disturbance Severity with Mobile Phone Keystroke Metadata: A BiAffect Digital Phenotyping Study	John Zulueta, Andrea Piscitello, Mladen Rasic, Rebecca Easter, Pallavi Babu, Scott A. Langenecker, Melvin McInnis, Olusola Ajilore, Peter C. Nelson, Kelly Ryan, Alex Leow	Yes	Yes	QN NRCT	Yes	Yes	Yes	Yes	Yes	*****
47.	An Observational Pilot Study using a Digital Phenotyping Approach in Patients with Major Depressive Disorder Treated with Trazodone	Čermák Jan, Pietrucha Slavomír, Nawka Alexander, Lipone Paola, Ruggieri Alessandro, Bonelli Annalisa, Comandini Alessandro, Cattaneo Agnese	Yes	Yes	QN NRCT	Yes	Yes	Yes	Can't tell	Yes	****
48.	Utility of Digital Phenotyping Based on Wrist Wearables and Smartphones in Psychosis: Observational Study	Yang Zixu, Heaukulani Creighton, Sim Amelia, Buddhika Thisum, Abdul Rashid Nur Amirah, Wang Xuancong, Zheng Shushan, Quek Yue Feng, Basu Sutapa, Lee Kok Wei, Tang Charmaine, Verma Swapna, Morris Robert J T, Lee Jimmy	Yes	Yes	QN D	Yes	Can't tell	Yes	Can't tell	Yes	***

### Study profile

#### Study setting

Twenty-three studies were conducted in the United States (26, 28, 40, 45, 47–50, 52–54, 56, 57, 59, 61, 69, 73–75, 77, 80, 82, 85, 86). Six studies were conducted in the United Kingdom (41, 43, 64, 67, 68, 76) and 4 studies were conducted in Denmark (65, 66, 83, 84). Three studies were conducted in South Korea (62, 63, 72), 3 studies were conducted in Taiwan (70, 78, 79) and one in Singapore (55). Two studies were conducted in Australia (58, 60). One study was conducted in Poland (44), one in Austria (46), one in Greece (71) and one in Czech Republic (81). Three studies were multi-country collaborations, with two being conducted in USA and India (51, 85) and one in UK, Netherlands, Spain (64) (Table 2).

#### Clinical profile

Studies focused on various disorders such as schizophrenia, bipolar disorder, and major depressive disorder (MDD) among others, and these were studied either independently or in conjunction with other disorders. Nineteen studies studied schizophrenia spectrum disorders, which included schizophrenia and schizoaffective disorder (28, 40, 41, 45, 47–49, 51–55, 57, 61, 69, 71, 75, 85). A multitude of symptoms were investigated in these studies such as early warning signs (EWS) of a relapse (28, 48, 57, 85), positive and negative symptoms (40, 41, 49, 51, 54, 55, 61, 69, 71), cognitive symptoms (45, 47) and general function (52, 75). Twelve studies focused on bipolar disorders (46, 56, 62, 63, 65, 66, 72–74, 76, 79, 83, 84), either independently or with other disorders. Various symptoms were investigated in these studies such as mood symptoms (mania, depression, mood fluctuations) (56, 62, 63, 66, 72–74, 83) and sleep/circadian rhythms (56, 79). Seven studies focus on MDD or depression (43, 44, 50, 55, 60, 68, 81) either as a primary diagnosis or within mixed clinical samples, and two of these studies examined MDD exclusively (60, 81). One study focused on anxiety disorders (50) and one on borderline personality disorders (67). Across the included studies, a diverse range of symptom domains were investigated. The most common areas of focus were mood symptoms such as depressive severity, manic episodes, mood fluctuations, and recurrence risk (56, 62, 63, 65, 66, 72–74, 79, 83). A smaller subset examined relapse and early warning signs (28, 48, 57, 85), sleep and circadian rhythm disturbances (53, 56, 79), and cognitive outcomes such as attention and executive functioning (52, 75). More specific symptoms included suicidality and self-harm risk (68), social anxiety symptoms (50), and functioning and health-related behaviours such as physical activity and weight management (52, 75). Six studies did not explicitly investigate clinical symptoms, instead focusing on feasibility, acceptability, or digital biomarkers of diagnostic groups (54, 58, 64, 67).

### Implementation of digital phenotypes

#### Data collection

Data were collected using either open-source platforms (28, 47, 52–54, 57, 64, 78, 85) or purposed built platforms (26, 40, 45, 56, 58, 61–63, 65, 66, 70, 74, 81, 83, 84). In sixteen studies, wearable devices were used (41, 43, 46, 48–50, 55, 60, 67, 71, 73, 75–77, 81, 86). Eleven studies mentioned the use of smartphone/wearable but did not mention an application that helped collect data (26, 40, 41, 61, 64, 71–73, 77, 86, 87) (Table 3).

#### Passive data

The most common types of passive data were geolocation data and accelerometer or physical activity data. Twenty-eight studies collected geolocation data (28, 40, 44–47, 49–60, 62, 69, 71–73, 76, 79, 80, 82, 85, 86), these data were captured through GPS signals, which were used to mobility patterns, circadian rhythms, and time spent at home or outside. Twenty-six studies collected accelerometer or physical activity data (28, 40, 41, 44–47, 49–51, 53, 55, 56, 58–62, 69, 72, 73, 76, 77, 79, 85, 86). Data were collected either through smartphone sensors (28, 40, 44–46, 49, 56–62, 85) or wearable devices (41, 43, 55, 71, 73, 75–77, 79, 81, 86) such as Fitbit and Empatica E4. Most studies collected basic activity measures such as step counts, movement intensity, and energy expenditure, while others analysed sedentary behaviour, sleep–wake cycles, and broader mobility patterns. In bipolar disorder, accelerometer data were often used to monitor psychomotor changes, circadian rhythm disruption, and relapse risk (62, 66, 73), whereas in schizophrenia spectrum disorders they were applied to examine negative symptoms, anhedonia, and illness severity (28, 41, 86). In MDD, activity patterns were associated with fluctuations in mood, cognitive performance, and functional outcomes (60, 77). Twenty-four studies collected communication metadata such as call and SMS logs (28, 40, 44–47, 49–53, 56–62, 67, 69, 72, 73, 85, 86). These data were typically used as proxies for social activity, isolation, and network stability. In schizophrenia spectrum disorders, reductions in call frequency and text messaging were linked to negative symptoms, relapse risk, and functional decline (28, 40, 69), while in bipolar disorder, changes in communication intensity often preceded mood episodes (62, 88). Studies in depression and mixed SMD samples similarly found that diminished outgoing communication was associated with increased symptom burden and lower functioning (44, 52, 60). Sleep and circadian rhythm indicators were passively monitored in 24 studies, most often derived from smartphone accelerometers, actigraphy, or wearable devices (28, 40, 41, 44–47, 49, 51, 53, 55–58, 60, 62, 69, 72, 73, 76, 77, 79, 85, 86). In bipolar disorder, studies demonstrated that reduced sleep duration, irregular circadian rhythms, and greater night-time activity predicted relapse or episode recurrence (56, 62, 73, 79). In MDD, accelerometer- and wearable-derived sleep metrics such as fragmentation, reduced total sleep time, and altered activity–rest cycles were associated with greater symptom severity and relapse risk (60, 77). Within schizophrenia spectrum disorders, passive sleep measurement highlighted increased sleep variability and rest–activity disruption as correlates of negative symptom severity and relapse (28, 41, 53). Multimodal approaches frequently paired sleep data with mobility and communication logs to capture a broader behavioural signature of illness (49, 85). Eight studies collected physiological signal data (41, 43, 55, 71, 77, 81, 86, 87), six studies collected keystroke dynamics (44, 47, 50, 58, 69, 80) and five studies collected app usage data (45, 67, 70, 73, 85). Physiological markers were investigated in schizophrenia and psychosis for autonomic dysregulation and symptom severity (41, 71, 86), in bipolar disorder to track mood fluctuations and sleep–wake disturbances (73, 81) and in depression to examine associations with cognitive and affective states (43, 77). Keystroke dynamics leveraged smartphone typing metadata such as latency, speed, and error rates to assess mood changes, psychomotor slowing, and cognitive performance; these were particularly relevant in bipolar disorder and depression (44, 50, 58, 80). App usage pattern data showed that reduced or altered engagement with digital platforms was linked to worsening mood and social withdrawal, particularly in schizophrenia and bipolar disorder (67, 70, 85).

#### Active data

Studies used several clinical data collected actively (see Table 2). The Brief Psychiatric Rating Scale (BPRS) was used in studies pertaining to psychotic disorders (28, 41, 57). Hamilton Depression Rating Scale (HDRS) (77, 83) and Young Mania Rating Scale (YMRS) (62, 66) were mainly used in studies that focused on bipolar disorder and depressive disorder. Patient Health Questionnaire-9 (PHQ-9) (41, 44, 58, 60) was used in 4 studies and Generalized Anxiety Disorder-7 (GAD-7) (44, 50, 58) were used in 3 studies of depression and mixed SMD populations. Four studies that focused on depression or depressive symptoms used Hamilton Depression Scale (HAMD) (63, 73, 77, 81). Other scales such as Montgomery-Åsberg Depression Rating Scale (MADRS) (43, 75), 6-Minute Walk Test (6-MWT) (75) and Social Interaction Anxiety Scale (SIAS) (50) also used.

#### Data preprocessing

Data pre-processing techniques varied across the studies (see Table 3). Seventeen studies did not describe any pre-processing steps (26, 28, 44–46, 51–55, 57, 60, 67, 70, 72, 74, 80, 82), whereas the remainder provided varying levels of detail. Studies focused on feature extraction from raw data streams, such as movement- and sleep-related metrics from accelerometers and wearables (62, 73, 76, 77), autonomic signals from physiological sensors (41), or circadian rhythm measures derived from smartphone use and location patterns (56). In some cases, features were application-specific; for example, one study extracted the average number of applications used per patient per day, filtering by screen-on activity and categorising apps by function (70). Other preprocessing efforts emphasised standardising formats and removing noise or outliers in physiological signals, such as heart rate variability and sleep data (55, 81, 86). Missing data were handled in different ways: some studies employed imputation or pairwise deletion to preserve sample size (47, 49, 58, 85), while others excluded incomplete or low-quality recordings (53). One study implemented a cleaning process that involved the exclusion of outliers in sleep and activity data, which indirectly pertains to removing incomplete or anomalous records (79). In another study, missing data was addressed through pairwise deletion, focusing on using available pairs of data without substituting or omitting all data related to a missing value when handling keyboard metadata, such as keyboard strokes and pressure of strokes (80). Finally, some studies aggregated data at the daily level to reduce noise and capture behavioural trends over time, including daily steps, heart rate, weight, and cognitive test performance (43, 79, 87). One study on schizophrenia aggregated data streams from multiple sources to create a comprehensive dataset for analysis, ensuring consistency and mitigating any gaps in individual data streams (85).

#### Data analysis

Data analysis approach through the studies are diverse and focused on various advanced techniques (see Table 3). A comprehensive description of the analytic procedures is presented in Supplementary File 2.

##### Neural network

Neural networks are ML models that learn complex patterns in data by processing inputs through interconnected computational layers. One study applied encoder-decoder neural network models to predict early warning signs of psychotic relapse from passive sensing data (57). The study centred on using encoder-decoder neural network models to analyse passive sensing data and predict EWS of psychotic relapse. The analysis involved two specific types of encoder-decoder models: Fully Connected Neural Network Autoencoder (FNN AD) and GRU Sequence-to-Sequence (GRU Seq2Seq). The models were trained on "days of relative health" data to establish normal behavioural patterns. Anomaly detection was then applied to identify deviations from these patterns, with high reconstruction errors signalling potential anomalies. Reconstruction errors were analysed to optimise sensitivity and specificity, and *post-hoc* analyses were conducted to compare behavioural features between anomalous and stable days to improve clinical interpretability.

##### Ensemble models

Ensemble models combine predictions from multiple ML algorithms to improve accuracy and robustness compared with using a single model. Four studies used ensemble models to enhance the accuracy and reliability of predictive models (50, 62, 68, 77). One study used ensemble models integrating circadian rhythm and passive sensing data to predict mood states in bipolar disorder (62). A study on suicide risk incorporated ensemble methods, including gradient boosting and random forests, to identify suicide risk from multimodal phone-based data (68). Ensemble methods were further used in monitoring changes in depression severity, leveraging multiple sensor inputs to robustly capture mood variations in a study focusing on depression (77). One study on social anxiety disorder utilized an ensemble of extreme gradient boosting models to identify digital biomarkers of social anxiety and focused on extracting subtle patterns from digital interactions that correlate with anxiety severity, demonstrating the strength of ensemble models in handling complex and subtle data patterns (50). Random forest algorithms were also used across studies to enhance prediction accuracy by combining multiple models (50, 62, 68, 85, 86). One employed the random forest algorithm to predict mood states from various biometric and environmental inputs (62). The model leveraged an ensemble of decision trees to handle high variability in mood disorder symptoms among patients, improving prediction reliability. Random forests were also used to classify anxiety severity from passive smartphone features, demonstrating the utility of ensemble techniques in detecting subtle symptom variations (50).

##### Support vector machines (SVMs)

Support Vector Machines (SVMs) are supervised ML algorithms used for classification or regression tasks by identifying optimal boundaries that separate different classes in high-dimensional data. SVMs were applied in three studies (46, 56, 68). One study that focused on bipolar disorder employed SVM for the classification of stability in bipolar disorder patients, analysing social rhythm metrics (56), while another study applied SVMs to distinguish between different illness states in bipolar disorder, demonstrating the algorithm's utility for high-dimensional behavioural data (46). Finally, SVMs were used alongside other machine learning methods to classify suicide risk, highlighting their potential clinical application in acute psychiatric settings (68). The SVM model was trained and validated using k-fold cross-validation to ensure robustness and prevent overfitting. This method divided the data into several subsets, with each subset being used once as a test set while the others formed the training set.

##### K-Means clustering

K-means clustering is an unsupervised machine-learning method that groups observations into clusters based on similarities in their characteristics without using predefined outcome labels. K-Means Clustering was employed in two studies that focused on schizophrenia (48, 69). K-Means Clustering was utilised to segment patient behavioural data into distinct clusters based on similarity in daily activity patterns, social interactions, and geographical movements collected through mobile devices. The clustering helped in identifying outlier patterns or anomalies that deviated significantly from typical behaviour patterns, which were predictive of potential relapse in schizophrenia patients (69). In another study, -Means Clustering was used to stratify patients into homogeneous groups based on digital phenotypes such as sleep patterns, online activity levels, and communication logs (48). This stratification enabled the researchers to tailor interventions and monitor the efficacy of treatments more effectively.

##### Correlation

Correlation analyses were applied in three studies to examine relationships between digital phenotypes and clinical outcomes (41, 58, 60). Correlations between passive sensing streams and self-reported measures were used to evaluate feasibility and acceptability in young people with depression (58). In a study that focused on MDD correlation analyses was used to link daily accelerometer-derived activity levels with fluctuations in depressive symptoms (60). Finally, correlations between wearable-derived physiological measures and symptom severity scores were explored (41). Specifically, heart rate variability, electrodermal activity, and skin temperature were correlated with clinical ratings (PANSS, BPRS).

##### Regression

Regression-based methods were applied across several studies (28, 44, 50, 64–66, 83–85). Two studies used logistic regression models to identify relapse predictors from multimodal passive and active data (85). One study used linear regression used to link behavioural data with self-reported psychological states (44). In studies focusing on bipolar disorder, mixed-effects regression models were used to account for repeated daily symptom ratings within individuals (66, 84). Linear and logistic regression were employed in subsequent studies to examine associations between smartphone-derived behavioural features and self-reported clinical symptoms (66). In another study, regression models were used to compare mobility patterns between individuals with bipolar disorder and those with unipolar depression (84). Another study multiple linear regression was utilized within a broader statistical framework to analyse the severity of mood disturbances based on keystroke metadata (80). Multiple linear regression models were used to analyse the relationship between various features derived from mobile phone use and accelerometer data, and the severity of mood disturbances as measured by the HDRS for depression and the YMRS for mania (80).

##### Cross-validation

Cross-validation was used to validate the robustness of predictive models in some studies (46, 56, 57, 68). One study employed leave-one-out cross-validation to evaluate the generalizability of the predictive models which ensured each participant's data was used for both training and testing, minimizing bias (56). Similarly, another study applied k-fold cross-validation to assess the stability of SVM models for detecting illness state transitions in bipolar disorder (46). In one study, *F*-tests and k-fold cross validation were used to compare models and validate the predictive significance (68). Cross-validation was incorporated in another study to evaluate neural network-based anomaly detection models, ensuring that predictions of relapse risk were generalisable across patients (57). In another study, 10-fold cross-validation was employed to train ensemble and random forest models, improving the reliability of mood state prediction in bipolar disorder (62). Finally, cross-validation was used on a study that focused on suicide risk when training ensemble and SVM models to confirm the stability of suicide risk prediction in an acute clinical sample (68).

### Study results

A comprehensive presentation of the results and findings is available in Supplementary File 3 (Table 4).

#### Phenotypes

Studies reported importance findings from phenotype data collected through passive sensing. One study reported that periods of depression were associated with reduced spatial activity and fewer visited locations, whereas manic episodes showed increased random movement patterns (65). Another study showed that GPS data could be used to recognise and predict mood state changes in bipolar disorder, with effective prediction achieved by identifying correlations between specific locations and mood stability or instability (46). Deviations from typical mobility patterns, when combined with other behavioural data, could signal relapse up to 72 h in advance was reported in another study (28).

Application usage data was also reported to be clinically informative. One study found that smartphone usage features, including app activity, correlated with symptom severity in social anxiety disorder (50). Another study observed that application use and activity types were associated with depressive and manic symptom fluctuations, supporting app engagement as a proxy for mood instability (44).

Sleep and circadian rhythms emerged as another key phenotype. One study demonstrated that reductions in sleep duration and efficiency predicted depressive relapse (64). Another study validated the feasibility of using digital tools to estimate sleep in schizophrenia, showing close correspondence between passive data and clinical measures (53). One study reported that disruptions in circadian rhythm metrics strongly correlated with mood fluctuations in bipolar disorder (56), while another study showed that circadian-based features improved the prediction of mood states when incorporated into machine learning models (62).

#### Disorder monitoring

Several studies reported how DP helped in monitoring of disorders (see Table 4). In schizophrenia, wearable-derived physiological measures such as heart rate variability, electrodermal activity, and skin temperature were significantly correlated with clinical severity ratings, supporting the feasibility of autonomic signatures as biomarkers of illness burden (41). Other schizophrenia studies highlighted the role of behavioural data (45, 47). One study reported that anxiety, psychotic symptoms, and poor sleep were central predictors in dynamic symptom networks, suggesting that smartphone-based monitoring could help identify which symptoms drive broader clinical deterioration (47). Another study showed that passively collected data on face-to-face social encounters reflected negative symptoms such as social withdrawal, further validating the use of mobile sensing for functional monitoring (45). One study in schizophrenia detected a 30% increase in symptom severity scores 48 h before a relapse based on passive data such as acceleration data, application usage data, call and message logs and location data along with BPRS, and where statistically significant increases predict relapse events, underscoring the potential of real-time monitoring in patient management (57).

In bipolar disorder, several studies used passive data to monitor mood states and circadian patterns (44, 56, 62, 65). One study found that social rhythmicity, inferred from phone activity, mobility, and sleep–wake cycles, was strongly correlated with self-reported mood states, underscoring the clinical value of circadian rhythm monitoring (56). This study showed an average correlation of 0.75 to their social rhythms as measured by the Social Rhythmic Metrics (SRM) and the strong correlation between social rhythms and mood states in bipolar disorder suggested targeted interventions could be beneficial. Studies also showed that reduced call and message frequency was associated with depressive states, whereas increased communication and mobility were often seen in manic or hypomanic states (65, 66). Finally, another study extended these findings by showing that smartphone-based behavioural metrics such as call activity and messaging were significantly related to depressive and manic symptom fluctuations (44).

For MDD, studies also highlighted the monitoring potential of DP (58, 60, 64). One study found that daily activity levels measured by accelerometers correlated with fluctuations in depressive symptoms, providing a dynamic view of illness burden (60). One study showed that changes in sleep duration and efficiency, collected via wearable devices, predicted depressive symptom changes, linking objective sleep patterns with clinical relapse (64). Another study further established feasibility by combining smartphone-based sensing with neuroimaging markers to monitor depression, demonstrating that multimodal monitoring is acceptable to patients (58). Finally, one study on MDD found that clinical assessment correlations with DP data showed a kappa coefficient of 0.61, which validated the effectiveness of digital tools in monitoring depressive symptoms, aligning closely with traditional clinical assessments (51).

Additionally, a study on anxiety disorders found that smartphone features, including app usage and communication logs, predicted social anxiety severity (50). One study demonstrated that wearables could monitor behavioural health outcomes such as physical activity and weight in individuals with SMD (75).

#### Relapse and recurrence prediction

Several studies across various disorders demonstrated the potential of DP for relapse and recurrence prediction. In schizophrenia, one study reported that anomalies in mobility, communication, and sleep predicted relapse up to 72 h in advance (28), while another study showed that neural network models could detect deviations from stable behaviour that preceded relapse (57). Additionally, it was found that integrating passive smartphone data improved relapse prediction accuracy to 73% among patients diagnosed with schizophrenia (85). In bipolar disorder, it was showed across studies that self-monitoring combined with call, text, and mobility data was strongly associated with illness activity and could predict relapse events (65, 66). In depression, it was found that that disrupted sleep predicted recurrence (64), while another study established the feasibility of combining smartphone data with neuroimaging to monitor relapse in young people (58).

#### Limitations of the studies

There were several limitations reported in most studies (see Table 4). The most common limitation was small sample size, which was reported in many studies (28, 44, 46, 56, 60, 65). For example, one study included only nine bipolar patients, with seven completing the study, limiting the generalisability of findings on social rhythms (56). In some cases, only a minority of participants experienced relapse, where 18 out of 60 contributed relapse data, potentially biasing results toward higher-risk subgroups (28). Many studies also reported short study duration as a limitation (28, 46, 56, 61, 64, 80) reducing the ability to capture long-term illness trajectories. One study noted that their brief follow-up restricted evaluation of longer-term relapse in MDD (64) and another study reported that the limited monitoring window constrained the assessment of keystroke-based markers over time (80). Finally, another study emphasised that a short observation period reduced the number of mood state transitions captured in bipolar disorder (46). Many studies noted dependence on smartphone or wearable technology, with missing data from device malfunctions, variable adherence, or battery issues influencing results (53, 54). One study mentioned the limitation of relying on passive data collection through smartphones, where any disruptions or changes in device usage patterns could influence the results (80). Studies also highlighted challenges with self-reported data, where recall bias or low completion rates in ecological momentary assessments could undermine reliability (56, 58, 65, 66). Self-reporting can introduce several biases, such as recall bias, social desirability bias, and reporting inaccuracies, which may not accurately represent the participants’ actual behaviours or states (9). A few studies described contextual confounders, such as environmental noise in inpatient wards influencing outcomes (68), or difficulties sustaining engagement in behavioural interventions (87). Some studies did not specify any limitations (53, 74, 85).

## Discussion

Consistent patterns in the broader DP literature help contextualise the findings of this review. Prior work has repeatedly highlighted substantial methodological variability across studies, including differences in device platforms, sampling frequencies, sensing modalities, feature definitions and preprocessing pipelines (10, 24). Commentaries and methodological analyses further highlight inconsistency in preprocessing, feature extraction and model-building practices as a core challenge limiting reproducibility and cross-study comparability (10, 11). The literature also shows that DP is frequently implemented in generalist mental health pathways rather than diagnosis-specific programmes, reflecting a growing interest in service-level monitoring and early-warning applications rather than narrow disorder-focused use cases (89). By situating our findings within this growing body of evidence, we demonstrate that the methodological inconsistency observed in our review is not incidental but reflects a system-level challenge that continues to limit comparability, generalisability, and translational readiness across the field.

A significant methodological decision in this review was to maintain a broad scope across device types, diagnostic groups, and study settings. This was intentional and essential to the purpose of the review. DP research is inherently diverse, with studies utilising a wide array of smartphones, commercial and research-grade wearables, and sensor platforms, and with implementation frequently occurring in generalist or mixed-diagnosis mental health services. Narrowing the review to a single disorder category or device class would have excluded a substantial proportion of existing evidence and artificially reduced the methodological heterogeneity that we aimed to characterise. By retaining broad inclusion criteria, this review was able to capture the full range of implementation approaches currently used in the field and to more accurately illustrate the lack of standardisation in data collection, preprocessing, feature extraction, and analytical workflows.

### Main findings

This review aimed classify the use of DP in mental health research by examining the clinical populations involved, the digital devices and data utilised, and the methodologies adopted across studies. A key observation from this review is the utility of DP for disorder monitoring and relapse prediction. Smartphones and wearable devices like the Apple Watch were commonly used to gather data, capitalising on their widespread availability and the non-invasive nature of data collection. A notable finding from this review was that eleven studies did not specify the data collection platform used to acquire DP data. These studies typically relied on in-house or study-specific software rather than publicly documented platforms such as Beiwe or AWARE-Light. Furthermore, these eleven studies were found to originate from only four recurring author groups (40, 61). However, insufficient reporting of platform characteristics including operating system, data security processes, and technical capabilities limits reproducibility, hampers comparison across studies, and creates uncertainty regarding scalability beyond controlled research settings.

The types of data collected varied widely, from simple metrics like step counts to more complex measures such as geolocation and app usage patterns. This diversity in data types underscores the multifaceted approach of DP, aiming to capture a comprehensive picture of an individual's behavioural and environmental context. The methodologies used in the analysed studies were highly varied, with techniques ranging from basic statistical models to more complex machine learning algorithms like neural networks and ensemble methods. This variety reflects the experimental nature of the field and the ongoing development of best practices in data analysis. However, the findings also highlight a key methodological challenge in the field: the substantial variability in how DP studies are implemented. Differences in device selection, sensing modalities, preprocessing pipelines and analytical approaches limit comparability across studies and complicate the interpretation of results. This methodological diversity reflects the exploratory stage of the field but also shows the need for clearer reporting standards and more consistent implementation practices.

While the studies included in this review demonstrate methodological heterogeneity, several broader patterns can nevertheless be identified. First, studies used DP primarily for behavioural monitoring and feasibility assessment, focusing on correlations between passive sensor data and clinical symptom measures. Second, several studies applied predictive modelling approaches, using ML or statistical models to identify symptom trajectories, relapse risk, or mood state changes from multimodal behavioural data. Third, some studies embedded DP within clinical monitoring or intervention contexts, where passive sensing was combined with active symptom reporting or clinical assessment to support ongoing patient management. These patterns suggest that methodological variation across studies often reflects differences in the intended purpose of DP, ranging from descriptive monitoring to predictive modelling and clinical application.

These patterns are broadly consistent with conceptual frameworks described in the wider DP literature. Foundational work has conceptualised DP as the continuous quantification of behaviour through passive and active data streams collected from personal digital devices, typically progressing through stages of data acquisition, preprocessing, feature extraction, and analytical modelling (4, 11). Our findings align with these frameworks in showing that most studies follow a similar methodological pipeline, although the specific implementation of each stage varies substantially across studies. In particular, the review highlights persistent variability in reporting data preprocessing procedures, feature definitions, and analytic pipelines (23). This divergence between conceptual methodological frameworks and their practical implementation underscores an ongoing challenge in the field and further supports calls for clearer reporting standards and methodological guidance for digital phenotyping research.

### Clinical & research applications of DP

Although this review focused on implementation methodologies, the findings also clarify how DP can be used across research and clinical contexts. Research applications primarily involve using passive and active sensing to explore feasibility, data quality, and associations between sensor-derived features and symptom patterns, as well as testing modelling approaches for prediction (28, 90). Clinically, the same methods point toward potential pathways for real-world intervention: features such as changes in mobility, sleep regularity, autonomic activity, or communication patterns, reported across the included studies could support early identification of symptom worsening, enabling automatic signposting to appropriate services, prompting timely clinical review, or triggering proactive outreach before crises emerge (23, 91). DP therefore sits at an early translational juncture, where research-focused implementations already demonstrate clear potential clinical utility, but routine use will require more consistent methodologies, transparent reporting, and standardised implementation frameworks.

#### Limitations & strengths

This systematic review, while comprehensive, has several limitations. The variability in study designs, populations, and methodologies across the included studies may introduce heterogeneity that could affect the comparability and generalizability of the findings. This heterogeneity makes it challenging to draw firm conclusions about the efficacy and utility of DP across different mental health conditions. Additionally, the scope of the review was restricted to studies published in English, potentially omitting relevant research conducted in other languages. This language restriction might limit the understanding of DP practices globally. The decision to focus on primary studies ensured direct comparison of implementation methods but necessarily excluded secondary analyses, which may have synthesised related evidence. Variation in terminology across DP research (“mobile sensing”, “passive monitoring”, “wearable data”) may also mean that some eligible studies were indexed under non-standardised keywords. As this review focused on implementation characteristics and employed a narrative synthesis, quantitative pooling and effect size estimation were not undertaken. While this approach limits direct comparison of clinical effect sizes across studies, it enabled a detailed examination of methodological heterogeneity that would not have been captured through meta-analytic techniques Finally, the rapid evolution of technology and methodologies in DP means that newer studies or advances may not have been included in this review. This field is advancing quickly, and keeping up to date with the latest research is essential for a comprehensive overview.

Several systematic and scoping reviews have examined DP in mental health, but most have focused primarily on clinical prediction, specific sensing modalities, or computational modelling approaches rather than the broader methodological pipeline through which DP studies are implemented. For example, previous reviews have summarised the use of passive smartphone sensing to infer behavioural markers of mental health or have evaluated the predictive performance of machine-learning models using sensor-derived data (23, 24, 29). Other reviews have focused on technical frameworks for multimodal sensing and data analytics or on ethical and conceptual considerations in DP (89, 92, 93). While these contributions have advanced understanding of the potential of DP, they provide limited synthesis of how studies operationalise DP across the full methodological pathway, including device selection, sensing modalities, preprocessing pipelines, feature extraction, and analytic strategies. By focusing specifically on the implementation characteristics of DP studies conducted in clinically diagnosed mental health populations, this review complements the existing literature by mapping the methodological decisions that shape how DP systems are deployed in practice. This implementation-focused perspective highlights the extent of methodological heterogeneity across studies and underscores the need for clearer reporting and greater standardisation to support reproducibility and translation into clinical settings.

#### Methodological heterogeneity

The marked methodological diversity observed in this review has direct implications for how DP findings can be interpreted and translated into practice. Differences in sensor configurations, sampling frequencies, data completeness thresholds, preprocessing pipelines, and modelling strategies mean that ostensibly similar phenotypes (e.g., “mobility”, “sleep disruption”, or “social activity”) are often operationalised in non-comparable ways across studies. Variation at each stage of the data lifecycle, device choice, on-device sensing, data transfer, cleaning, feature extraction, and labelling can substantially affect the reproducibility and external validity of reported associations (10). This heterogeneity limits the extent to which effect sizes or thresholds can be generalised across settings (4). While several studies point toward promising clinical applications of DP, the substantial methodological variation across devices, data streams, and analytic approaches means that disorder-specific recommendations should be interpreted cautiously. Rather than aiming to produce condition-specific guidance, our review maps how these methodological differences shape the evidence base and identifies the areas where greater standardisation is needed to support more confident clinical translation.

#### Ethical considerations

Ethical considerations are fundamental to the advancement of DP and are relevant across all global contexts, from high-income clinical settings to community-based care in low- and middle-income countries (10). Because DP relies on continuous, high-granularity behavioural and physiological data, concerns regarding privacy, autonomy, meaningful consent, and data governance arise universally (94). These issues affect all users regardless of geography, given the potential for re-identification, opaque algorithmic processing, and challenges in ensuring that individuals fully understand what is being collected and how it may be used (94). In parallel, broader questions of fairness, representativeness, and algorithmic bias require global attention, as models trained predominantly on Western, urban, or majority-population samples may perform poorly or inequitably when applied across diverse cultural, linguistic, or socioeconomic groups (95). When viewed through a global health lens, additional concerns emerge related to disparities in digital literacy, regulatory capacity, and structural vulnerability, which may increase risks of misuse or unequal benefit in under-resourced settings (10, 96). Addressing these ethical challenges, ranging from universal issues of privacy and autonomy to context-specific questions of equity and governance will be essential to ensure that DP is implemented responsibly and supports individuals across diverse global populations.

#### Implications and future directions

The review's findings suggest several implications for future research in DP. First, there is a clear need for standardised protocols in data collection, preprocessing, and analysis to ensure that results are robust, reproducible, and comparable across studies. Developing consensus guidelines and best practices could facilitate this standardisation process (10). The variability observed across studies also highlights specific components of the DP pipeline where clearer reporting and greater methodological consistency would substantially improve comparability and reproducibility. First, sensor data acquisition parameters are rarely described in detail. Several studies reported collecting GPS or accelerometer data but did not specify sampling frequency, duty cycles, or conditions under which sensors were activated, making it difficult to compare behavioural measures across studies or replicate sensing configurations. Second, data preprocessing procedures were inconsistently documented. As observed in this review, a substantial proportion of studies did not report preprocessing steps at all, while others applied filtering, outlier removal, aggregation, or imputation without clearly describing the criteria used. Because preprocessing decisions directly affect derived behavioural metrics, clearer documentation of cleaning procedures, missing-data handling, and temporal aggregation methods would improve transparency. Third, there was considerable variability in feature extraction and feature definitions, particularly for commonly used digital phenotypes such as mobility, sleep, and communication behaviour. Explicitly defining how such features are derived from raw data would facilitate cross-study comparison and interpretation. Fourth, data collection platforms and technical infrastructure were not consistently reported; several studies relied on smartphone or wearable devices without specifying the software platform or application used for data capture. Providing details on sensing platforms, operating system constraints, and device models would help clarify how technical configurations influence data availability and quality. Taken together, these findings suggest that future DP research would benefit from establishing minimum reporting elements for the DP pipeline, including sensing configurations, preprocessing workflows, feature definitions, and analytic procedures. In addition to having methodological framework, such reporting guidance could function as practical documentation standards that support transparency, reproducibility, and cumulative methodological development in the field.

The findings of this review also highlight several methodological practices that may improve the quality and reproducibility of DP research. Across the included studies, clearer reporting of sensing configurations, preprocessing workflows, feature extraction procedures, and analytical pipelines would substantially improve transparency and comparability. Studies that explicitly documented data collection platforms, device specifications, and missing-data handling procedures provided greater clarity regarding how behavioural features were derived and interpreted. Future work would benefit from adopting more consistent reporting practices across these stages of the digital phenotyping pipeline. Detailed methodological guidance for sensor-based data collection and reporting in DP research has recently been proposed elsewhere, including recommendations on documenting sensing configurations, preprocessing procedures, and feature generation workflows (10).

Additionally, while the focus has been on certain clinical populations, expanding DP efforts to other mental health conditions could provide insights into the generalizability and utility of these techniques across the broader spectrum of psychiatric disorders. Integrating DP data with traditional clinical assessment tools could enhance diagnostic accuracy and the personalization of treatment strategies. Furthermore, DP could help LMICs (10, 97, 98), where mental health services are often under-resourced and the burden of diseases like depression and bipolar disorder is substantial (10, 99), DP can provide a low-cost, scalable method of monitoring and managing health conditions in real-time. Furthermore, DP may also have important applications beyond clinical populations. Continuous behavioural monitoring has the potential to support early detection of symptom changes, identification of individuals at elevated risk, and population-level monitoring of behavioural indicators associated with mental health. For example, DP approaches could be applied to high-risk groups, such as individuals with a family history of SMDs or those experiencing early subclinical symptoms, to identify behavioural changes that precede illness onset. In addition, passive sensing approaches may support preventive mental health strategies by identifying behavioural patterns associated with stress, sleep disruption, or social withdrawal in community populations.
