# Supplementary material for: Digital phenotyping for mental health conditions: a systematic review of implementation and application
Source: Front Digit Health. 2026 Jul 9;8:1772744. doi: 10.3389/fdgth.2026.1772744 (PMC13391508; doi:10.3389/fdgth.2026.1772744)
Supplement: Supplementary file 1 [file Datasheet1.docx]

# Supplementary Material

## Methods

### Search Strategy

The search strategy included: "mental disorder*" OR "mental illness*" OR "psychiatric disorder*" OR "psychological disorder*" OR "schizophrenia" OR "bipolar disorder" OR "major depressive disorder" OR "depression" OR "psychosis"

AND

"digital phenotyping" OR "passive sensing" OR "mobile sensing" OR "smartphone sensing" OR "digital biomarkers" OR "sensor data" OR "wearable device*" OR "smartphone*" OR "smartwatch*" OR "GPS" OR "accelerometer" OR "mobile health" OR "mhealth" OR "mobile app*" OR "digital health" OR wearable electronic devices"

NOT

"telepsychiatry" OR "online counselling" OR "online therapy" OR "self-help app*" OR "virtual therapy" OR "digital journaling" OR "cancer" OR "weight loss" OR "nutrition"

**Example Search on PubMed:**
("mental disorders"[MeSH Terms] OR "schizophrenia"[MeSH Terms] OR "bipolar disorder"[MeSH Terms] OR "depression"[MeSH Terms] OR "psychotic disorders"[MeSH Terms]) AND ("digital phenotyping"[All Fields] OR "passive sensing"[All Fields] OR "mobile sensing"[All Fields] OR "smartphone"[All Fields] OR "wearable electronic devices"[MeSH Terms] OR "accelerometer"[All Fields] OR "digital biomarkers"[All Fields]) NOT ("telepsychiatry"[MeSH Terms] OR "online counseling"[All Fields] OR "virtual therapy"[All Fields] OR "weight loss"[MeSH Terms] OR "cancer"[MeSH Terms])
