# Supplementary material for: Digital phenotyping for mental health conditions: a systematic review of implementation and application
Source: Front Digit Health. 2026 Jul 9;8:1772744. doi: 10.3389/fdgth.2026.1772744 (PMC13391508; doi:10.3389/fdgth.2026.1772744)
Supplement: Supplementary file 2 [file Datasheet2.docx]

| **Study ID** | **Study Information** | | **Data Collected** | | **Data Collection Platform(s)** | **Data Storage** | **Data Pre-Processing** | **Data Analysis** |
| --- | --- | --- | --- | --- | --- | --- | --- | --- |
|  | **Title** | **Authors** | Clinical Data | Other Data |  | | | |
|  | Automatic detection of social rhythms in bipolar disorder | Abdullah, Saeed; Matthews, Mark; Frank, Ellen; Doherty, Gavin; Gay, Geri; Choudhury, Tanzeem | Social Rhythm Metric (SRM-5) | Self-reported mood and energy levels | MoodRhythm app | Local Storage on Smartphones: Initially, all the sensor data collected through the MoodRhythm app—such as accelerometer readings, ambient sound characteristics (without recording actual audio for privacy), location data, and communication patterns—were stored locally on the participants' smartphones. This local storage allowed the app to accumulate data throughout the day without requiring a constant internet connection.  Secure Transmission to Remote Server: Periodically, the data stored on the smartphones was securely transmitted to a remote study server. The secure server, managed by the research team, served as the central repository for all the collected data, where it could be further analyzed and processed. | Not mentioned | Feature Set Selection: Based on the pre-processed data, the study identified a set of features to use in the analysis, including location data (e.g., number of location clusters, distance traveled), conversation frequency (inferred from audio data), and duration of non-sedentary activity. These features were chosen because they are indicative of social and physical activity patterns relevant to the assessment of bipolar disorder symptoms.  Statistical Modeling for SRM Score Inference: The study used statistical learning techniques to model the relationship between the selected features and the SRM scores. Support Vector Regression (SVR) was employed to predict the continuous SRM scores based on the sensor data. This approach allowed the researchers to assess how closely the model's predictions matched the actual SRM scores reported by participants.  Model Validation and Performance Evaluation: The accuracy of the SVR model was evaluated using a 10-fold cross-validation technique. This involved dividing the data into ten parts, using nine parts for training the model and one part for testing, and repeating this process ten times. The performance of the model was quantified using the root mean square error (RMSE) between the predicted and actual SRM scores.  Classification of Stability: Beyond predicting SRM scores, the study also aimed to classify days as stable or unstable based on a threshold SRM score of 3.5. A Support Vector Machine (SVM) classifier was used for this binary classification task, utilizing the same feature set as the regression analysis. The classifier's performance was assessed in terms of precision and recall.  Feature Importance and Model Robustness: The study conducted feature ranking to identify which features were most predictive of SRM stability. Additionally, the robustness of the classification model was evaluated by examining the confidence scores associated with its predictions, indicating the model's certainty in its classifications.  Correlation Analysis: The study also explored the correlation between the sensor-derived features and self-reported mood and energy levels, providing further insight into the relationship between participants' daily activities, social interactions, and their mental health states. |
|  | Predicting Early Warning Signs of Psychotic Relapse From Passive Sensing Data: An Approach Using Encoder-Decoder Neural Networks | Adler, Daniel A; Ben-Zeev, Dror; Tseng, Vincent W-S; Kane, John M; Brian, Rachel; Campbell, Andrew T; Hauser, Marta; Scherer, Emily A; Choudhury, Tanzeem; | Brief Psychiatric Rating Scale (BPRS) | Ecological Momentary Assessment (EMA) Survey Frequency and Timing: Participants completed EMA surveys three times daily over seven consecutive days. | CrossCheck | Not mentioned | Feature Creation: Hourly features were derived from raw sensor data, with additional features for the day of the week and hour of the day. Missing Data Handling: Type 1 missing data (where some but not all features are missing) was imputed with a "0", and type 2 missing data (where all features are missing for a given hour) was filled using the mean value of the feature for that hour. Location data was assumed to remain the same as the last recorded location if missing​​. | Encoder-Decoder Neural Network Models The core of the data analysis was based on encoder-decoder neural network models. These models are designed to handle sequential data by first encoding the input data sequence into a fixed-dimensional context vector (encoding phase) and then decoding this context vector to reconstruct the input sequence or predict future data points (decoding phase). The study employed two types of encoder-decoder models:  Fully Connected Neural Network Autoencoder (FNN AD): This model architecture utilizes fully connected layers for both the encoding and decoding phases. It's suitable for capturing the linear relationships and interactions within the data. GRU Sequence-to-Sequence (GRU Seq2Seq): This model uses Gated Recurrent Units (GRUs) in both the encoder and decoder phases. GRU Seq2Seq models are particularly effective for handling temporal dependencies in time-series data, making them ideal for analyzing the sequential passive sensing data collected in the study. Anomaly Detection System The anomaly detection component of the study aimed to identify deviations from normal patterns that could indicate early warning signs of a psychotic relapse. The encoder-decoder models were trained on data labeled as "days of relative health" (DRH) to learn the normal behavioral patterns of participants. The trained models were then used to reconstruct the input sequences, and the reconstruction error was calculated as the difference between the actual data points and their reconstructed counterparts. High reconstruction errors were indicative of anomalies.  Model Training and Validation Data Splitting and Cross-validation: The data for each participant was divided into equal-length nonoverlapping subsequences. These subsequences were further split into different datasets for training, cross-validation, and testing purposes. A notable aspect was the division of data based on the proximity to relapse events, with separate datasets for "near relapse" periods and DRH. Model Optimization: The study optimized the models based on the True Positive Rate (TPR) to False Positive Rate (FPR) ratio, focusing on achieving a balance between sensitivity (identifying potential relapses) and specificity (avoiding false alarms). Post-hoc Analysis for Clinical Interpretation After identifying anomalies, a post-hoc analysis was conducted to provide clinical interpretation of the detected anomalies. This involved comparing features on anomalous days to those on days of relative health, using statistical measures like Cohen's d to assess the effect size of continuous features. |
|  | SmartSense-D: A safety, feasibility, and acceptability pilot study of digital phenotyping in young people with major depressive disorder | [Andres Camargo](https://journals.sagepub.com/doi/10.1177/20552076251330509?url_ver=Z39.88-2003&rfr_id=ori:rid:crossref.org&rfr_dat=cr_pub%20%200pubmed#con1),  [Scott D Tagliaferri](https://journals.sagepub.com/doi/10.1177/20552076251330509?url_ver=Z39.88-2003&rfr_id=ori:rid:crossref.org&rfr_dat=cr_pub%20%200pubmed#con2), [Lianne Schmaal](https://journals.sagepub.com/doi/10.1177/20552076251330509?url_ver=Z39.88-2003&rfr_id=ori:rid:crossref.org&rfr_dat=cr_pub%20%200pubmed#con10), [Tianyi Zhang](https://journals.sagepub.com/doi/10.1177/20552076251330509?url_ver=Z39.88-2003&rfr_id=ori:rid:crossref.org&rfr_dat=cr_pub%20%200pubmed#con4), [Zamantha Munoz](https://journals.sagepub.com/doi/10.1177/20552076251330509?url_ver=Z39.88-2003&rfr_id=ori:rid:crossref.org&rfr_dat=cr_pub%20%200pubmed#con5), [Pemma Davies](https://journals.sagepub.com/doi/10.1177/20552076251330509?url_ver=Z39.88-2003&rfr_id=ori:rid:crossref.org&rfr_dat=cr_pub%20%200pubmed#con6), [Mario Alvarez-Jimenez](https://journals.sagepub.com/doi/10.1177/20552076251330509?url_ver=Z39.88-2003&rfr_id=ori:rid:crossref.org&rfr_dat=cr_pub%20%200pubmed#con7), [Niels van Berkel](https://journals.sagepub.com/doi/10.1177/20552076251330509?url_ver=Z39.88-2003&rfr_id=ori:rid:crossref.org&rfr_dat=cr_pub%20%200pubmed#con8), [Vassilis Kostakos](https://journals.sagepub.com/doi/10.1177/20552076251330509?url_ver=Z39.88-2003&rfr_id=ori:rid:crossref.org&rfr_dat=cr_pub%20%200pubmed#con9), [Lianne Schmaal](https://journals.sagepub.com/doi/10.1177/20552076251330509?url_ver=Z39.88-2003&rfr_id=ori:rid:crossref.org&rfr_dat=cr_pub%20%200pubmed#con10) | MINI diagnostic interview (DSM-V criteria for MDD), QIDS-16 at baseline and follow-up | Demographics (age, sex assigned at birth, gender identity, education, employment, living situation); debriefing questionnaire on usability, comfort, privacy concerns | AWARE-Light smartphone sensing app (Android only); GENEActiv wrist-worn actigraphy device; EMA surveys via AWARE-Light app | Not mentioned | No imputation applied; missing data retained to avoid bias.  Sensor-level validity assessed independently; day-level validity defined by location sensor completeness.  Actigraphy features extracted using GGIR R package.  EMA mood scores averaged; variability calculated as SD. | Descriptive Statistics: For feasibility, safety, and acceptability metrics.  Pearson Correlations: To assess relationships between sleep, physical activity, mood, and smartphone sensor data.  Linear Mixed Models (LMMs):   - Participants as random effects - Fixed effects: age, sex, predictor (digital measure), time (baseline vs follow-up), and predictor × time interaction - Implemented using lmer from the lme4 R package - FDR correction applied for multiple comparisons |
|  | Relapse Prediction in Schizophrenia through Digital Phenotyping | Barnett, Ian; Torous, John; Staples, Patrick; Sandoval, Luis; Keshavan, Matcheri; Onnela, Jukka-Pekka | Warning Symptoms Scale | Survey questions for: depression, sleep quality, psychosis,anxiety, taking medication | Beiwe app | Not mentioned | GPS Data Pre-processing:  The latitude-longitude data collected from GPS were projected onto a 2D plane for analysis. This data were then converted into mobility trajectories, consisting of "flights" (periods of movement at a constant speed in a constant direction) and "pauses" (periods of non-movement). Imputing Missing GPS Data:  To handle gaps in the mobility trajectories due to missing GPS data, a statistical approach based on weighted resampling of observed trajectories was used to impute the missing parts, effectively filling in gaps with a mixture of flights and pauses. Feature Extraction from Mobility Data:  From the processed mobility trajectories, daily mobility features were summarized, including daily distance traveled, daily time spent at home, and the number of daily significant locations visited. Feature Extraction from Sociability Data:  Using anonymized call and text message logs, daily sociability features were estimated, such as the total duration of calls, number of missed calls, and number of text messages sent. Handling Missing Observations:  For both active and passive data streams, methods were implemented to accommodate missing observations, ensuring that the analysis could proceed even with incomplete data. | Time Series Decomposition: The researchers first decomposed the multivariate time series data for each collected feature (e.g., mobility and sociability metrics) into its constituent parts, separating the overall trend from the residual behaviors or errors. This included incorporating a component to account for weekly patterns, acknowledging the cyclical nature of human behavior over the course of a week.  Standardization of Errors: To facilitate anomaly detection, errors from the trend decomposition were standardized. The team ranked these errors across all days for which data were available for each feature. They then transformed these ranks to the standard normal distribution using the probability integral transform, enabling the application of statistical tests that assume normally distributed data.  Anomaly Detection via Hotelling’s T2 Test: Utilizing the standardized errors, the researchers applied Hotelling’s T2 test, a multivariate statistical test, to identify significant deviations from expected behavior on a daily basis. This test allowed for the simultaneous consideration of multiple features to determine if the behavior on a given day was anomalously different from the norm.  Adjustment for Multiple Comparisons: Given the extensive number of comparisons made due to daily testing over several months, the researchers employed a bootstrapping technique to correct for the possibility of false positives. This involved generating a null distribution for the largest test statistic across all days by resampling the error components of the time series. The threshold for declaring a test result statistically significant was then established based on this null distribution, ensuring a controlled rate of false discoveries.  Comparative Analysis of Anomaly Rates: The team compared the rate of detected anomalies during different periods, specifically examining whether the frequency of anomalies increased within the 2 weeks leading up to a clinical relapse compared to other periods. This comparison aimed to validate the hypothesis that behavioral changes detectable through digital phenotyping data could serve as early indicators of relapse.  Development of a Predictive Model: The culmination of the analysis involved leveraging the identified behavioral anomalies and their temporal distribution to build a statistical model capable of predicting schizophrenia relapse. This model sought to harness the patterns found in the passive and active digital phenotyping data to forecast impending relapses, potentially allowing for timely interventions. |
|  | The Positivity Offset Theory of Anhedonia in Schizophrenia: Evidence for a Deficit in Daily Life using Digital Phenotyping | Lisa A. Bartolomeo, Ian M. Raugh, Gregory P. Strauss | Ecological momentary assessment, self-reports of positive and negative affect and arousal, PANSS scores, and clinical diagnostic interviews | cognitive functioning measures | Beiwe | N/A | N/A | All analyses were conducted using SPSS v.27, except for multi-level supplemental analyses performed in R. The positivity offset and negativity bias were calculated using regression parameters from the equation E = Ax + b, where E is either unipolar positivity or negativity ratings and A is mean arousal rating. Positivity offset scores were derived as the difference between positive and negative intercepts, and negativity bias scores as the difference between negative and positive slopes. Intercept and slope values were averaged across days and summarized weekly. Within-group paired t-tests compared positivity and negativity intercepts/slopes, and one-way ANOVAs assessed group differences in these parameters. Pearson correlations examined relationships between positivity/negativity parameters and passive digital phenotyping variables (accelerometry, geolocation) as well as clinical measures (BNSS, PANSS). Additional preliminary and exploratory analyses included mixed-model ANOVAs and univariate ANOVAs for group, sex, and diagnosis effects, with LSD post-hoc tests where appropriate. |
|  | The Dynamic Association Between Physical Activity and Psychological Symptoms in Young People With Major Depressive Disorder: An Active and Passive Sensing Longitudinal Cohort Study | [Rosalind Baynham](https://pubmed.ncbi.nlm.nih.gov/?term=%22Baynham%20R%22%5BAuthor%5D), [Andres Camargo](https://pubmed.ncbi.nlm.nih.gov/?term=%22Camargo%20A%22%5BAuthor%5D), [Simon D'Alfonso](https://pubmed.ncbi.nlm.nih.gov/?term=%22D%27Alfonso%20S%22%5BAuthor%5D), [Tianyi Zhang](https://pubmed.ncbi.nlm.nih.gov/?term=%22Zhang%20T%22%5BAuthor%5D), [Zamantha Munoz](https://pubmed.ncbi.nlm.nih.gov/?term=%22Munoz%20Z%22%5BAuthor%5D), [Pemma Davies](https://pubmed.ncbi.nlm.nih.gov/?term=%22Davies%20P%22%5BAuthor%5D), [Mario Alvarez‐Jimenez](https://pubmed.ncbi.nlm.nih.gov/?term=%22Alvarez%E2%80%90Jimenez%20M%22%5BAuthor%5D), [Niels van Berkel](https://pubmed.ncbi.nlm.nih.gov/?term=%22van%20Berkel%20N%22%5BAuthor%5D), [Vassilis Kostakos](https://pubmed.ncbi.nlm.nih.gov/?term=%22Kostakos%20V%22%5BAuthor%5D), [Lianne Schmaal](https://pubmed.ncbi.nlm.nih.gov/?term=%22Schmaal%20L%22%5BAuthor%5D), [Scott D Tagliaferri](https://pubmed.ncbi.nlm.nih.gov/?term=%22Tagliaferri%20SD%22%5BAuthor%5D) | MINI diagnostic interview (DSM-V criteria for MDD); QIDS-16 at baseline and follow-up.  Demographic data (age, self- identified sex and self- identified gender) | COVID lockdown status during data collection. | AWARE-Light smartphone app (Android only); GENEActiv wrist-worn actigraphy device; EMA surveys via AWARE-Light app | EMA responses sent to secure study database; actigraphy data processed locally using GGIR R package | Actigraphy: processed using GGIR (v2.9–0); ENMO thresholds applied (light ≥30, moderate ≥100, vigorous ≥400); daily and typical activity levels derived via person-mean and grand-mean centering.  EMA: positive/negative affect averaged across two daily timepoints; stress and anxiety scored once daily. | Software & Significance Threshold   - Analyses were conducted using IBM SPSS Statistics v29.0. - A significance level of α = 0.05 was used to control Type I error.   Stepwise Analytical Strategy  Step 1: Pearson Correlations   - - Assessed relationships between covariates (age, sex, baseline depression, COVID-19 lockdown status) and physical activity intensities (light, moderate, vigorous, sedentary).   - Also tested associations with EMA-derived psychological outcomes: stress, anxiety, positive affect, and negative affect.   - Purpose: To check for multicollinearity before model building.   Step 2: Multi-Level Linear Mixed Models (MLMMs)   - Used random intercepts for participants to account for repeated measures. - Fixed effects included:   - Level 1 (within-person): Daily fluctuations in physical activity (person-mean centered).   - Level 2 (between-person): Typical physical activity levels (grand-mean centered).   - Covariates: Age, sex, baseline depression. - Models were run separately for each psychological outcome:   - Model 1: Stress   - Model 2: Anxiety   -  Model 3: Positive affect   -  Model 4: Negative affect   Step 3: Multicollinearity Adjustment   - Due to high correlation between light and moderate activity, models were run twice:   - One with light activity included, moderate excluded.   - One with moderate included, light excluded.   Step 4: Sensitivity Analysis   - COVID-19 lockdown status was added as a covariate to test robustness of associations. |
|  | Use of Multimodal Technology to Identify Digital Correlates of Violence Among Inpatients With Serious Mental Illness: A Pilot Study | Ben-Zeev, Dror; Scherer, Emily A.; Brian, Rachel M.; Mistler, Lisa A.; Campbell, Andrew T.; Wang, Rui | Psychotic Symptom Rating Scales, Beck Depression Inventory–Second Edition (BDI-II), Green-Paranoid Thoughts Scale (G-PTS) | ecological momentary assessment (EMA: affect, environmental conditions, delusions, substance cravings, withdrawal symptoms, suicidal ideation, violent ideation and behavior | Adapted smartphones with specialized data collection software, utilizing sensors embedded in the devices | Not mentioned | Not mentioned | Nonlinear mixed-effects models were fit for each violence outcome and for each sensor and EMA predictor, accommodating the dichotomous outcome and the nonindependence of observations from the same individual. Multivariate models included features found to be significant in bivariate models, focusing on the exploratory nature of the analyses to identify all correlates of violence without adjustment for multiple comparisons. |
|  | Mobile Behavioral Sensing for Outpatients and Inpatients With Schizophrenia | Ben-Zeev, Dror; Wang, Rui; Abdullah, Saeed; Brian, Rachel; Scherer, Emily A; Mistler, Lisa A; Hauser, Marta; Kane, John M; Campbell, Andrew; Choudhury, Tanzeem |  | Questionnaire aimed to evaluate the feasibility and acceptability of the smartphone-based sensing approach | Smartphones installed with study-specific software developed by the research group | The study's storage plan for the data collected through mobile behavioral sensing involved securely storing the data on the smartphones until it could be transmitted to a study server. This process occurred when internet connectivity was available. Specifically, for outpatients, the data were transmitted to the server nightly, reflecting their likely regular access to internet connections. In contrast, inpatients had their data transmitted at the end of the week. | Ambient Sound Capture (Pre-processing for Human Speech Detection):  The microphone was activated every 2 minutes to capture ambient sound. Real-time data processing was employed to identify features indicative of human speech, such as energy levels, relative spectral entropy, and autocorrelation peak values. To protect privacy, the system did not record or transmit audio recordings. Instead, it processed the data in real-time and stored features useful for inferring the presence of human speech but insufficient to reconstruct speech content. Movement Detection (Feature Extraction from Accelerometer Data):  The smartphone accelerometers detected movement, and the system generated and stored an activity rating every 2 seconds. The activity ratings categorized movement as active or sedentary, allowing for the analysis of participants' activity levels without needing detailed movement data. Location Data Handling:  For outpatients, location data were captured using Android location services, which combined information from GPS, WiFi, and cellular networks for optimized location estimates. This process required minimal pre-processing, focusing on fusing different location data sources. Inpatients' location tracking involved a more granular approach using Bluetooth beacons installed throughout the unit. The smartphone's Bluetooth sensor received signals from the beacons, and the study software recorded participants’ locations based on proximity to these beacons. | Not specified |
|  | Using wearable technology to detect the autonomic signature of illness severity in schizophrenia | Cella Matteo a, Okruszek Łukasz, Lawrence Megan a, Zarlenga Valerio a, He Zhimin a, Wykes Til | ositive and Negative Syndrome Scale (PANSS), Time Use Survey |  | mHealth, Empatica E4 | The data collected by the wearable devices were stored directly on the device memory during the data collection period. After the six-day data collection period, the information was downloaded from the device memory to the study server during face-to-face meetings with the participants. | Exclusion of Short Recordings: Initially, recordings shorter than 60 minutes were excluded. This decision was based on the notion that some heart rate variability (HRV) parameters, like SDNN (standard deviation of NN intervals), may be less informative over shorter recording periods. This step aimed to focus on longer, more representative data segments that likely included a range of daily activities. Selection of Longer Recordings: For analysis, only data segments that included at least eight continuous hours of recording were considered. This threshold was chosen because all participants had several such segments, and these longer durations were deemed more likely to encompass a diverse array of daily activities, providing a more comprehensive view of each participant's autonomic function. Data Handling for Different Sensors: Electrodermal Activity (EDA): The EDA data, which measures skin conductivity and assesses sympathetic nervous system arousal, was collected at a frequency of 4 Hz. The data was processed using Ledalab for MATLAB, a software tool specifically designed for analyzing EDA. This processing included artifact removal to ensure that the EDA measurements reflected true physiological responses rather than noise or errors. Heart Rate Variability (HRV): The heart rate data were derived from the blood volume pulse recorded by a photoplethysmography sensor, and inter-beat intervals (IBI) were extracted. HRV parameters such as SDNN and RMSSD (root mean square of successive differences) were calculated using these IBI values. The Empatica device's algorithms were used for initial HRV calculation, followed by further processing with Kubios HRV, a software package known for its robust HRV analysis capabilities. Movement Data: Movement was monitored using a 3-axis accelerometer, which measures acceleration in three dimensions. An index of overall movement was derived using a standard Euclidean metric, which summed the accelerometer readings across all axes for the total recording sample length, thus producing an indicator of the overall extent of movement during the recording period. | Correlation Analysis: The first step involved examining the relationship between various physiological variables. This was done using Spearman rho correlation coefficients to assess the reliability and consistency of the parameters extracted from the devices. For example, correlations between different heart rate variability (HRV) measures such as mean RR intervals, SDNN, and RMSSD were calculated to validate the data's internal consistency. Group Differences: To explore differences between the schizophrenia and control groups in terms of HRV, movement, and other physiological measures, the researchers used a set of t-tests and univariate ANOVAs. Non-parametric tests were also employed for data that did not meet normal distribution assumptions, such as EDA and ACC (accelerometer data) values. Specific physiological measures such as SDNN and RMSSD showed significant differences between groups, indicating lower autonomic regulation in participants with schizophrenia. These results were further explored using post-hoc analyses controlling for potential confounders like age, time spent in structured activities, and movement levels. Correlations with Clinical and Functional Measures: In the final stage, the relationship between physiological measures and clinical outcomes (such as symptoms and functioning) was assessed. Again, Spearman rank correlations were utilized, focusing on associations between HRV measures, movement data, and clinical scores on the PANSS scale and functioning assessments. Multiple correlation adjustments were made to control for potential false discoveries, setting a more conservative significance threshold to ensure the robustness of the findings. For example, a p-value threshold was adjusted to 0.008 to account for multiple testing. |
|  | Mood Prediction of Patients With Mood Disorders by Machine Learning Using Passive Digital Phenotypes Based on the Circadian Rhythm: Prospective Observational Cohort Study | Cho, Chul-Hyun; Lee, Taek; Kim, Min-Gwan; In, Hoh Peter; Kim, Leen; Lee, Heon-Jeong | Interviews | Daily Mood Scores: Participants used a smartphone app (eMoodchart) to record their daily mood states on a simple scale. This represents an active form of data collection where participants directly input their mood assessments. | eMoodchart | Secure servers for storing the collected data. Specifically, data collected from wearable devices and the eMoodchart smartphone app were transmitted and stored on these servers. | Feature Extraction from Passive Data:  Basic features were derived from four main data categories: light exposure, steps, sleep, and heart rate. These features were designed to capture aspects of circadian rhythms and physical activity that could influence mood states. Light exposure was analyzed based on average values during specific time slots, particularly focusing on bedtime and daytime, adjusted for seasonal variations in day length. Activity levels were quantified through total steps recorded within specified periods (bedtime and daytime). Sleep data included metrics such as sleep duration, quality, and patterns of sleep onset and offset. Heart rate data underwent a cosinor analysis to extract parameters like amplitude, acrophase (peak), mesor (mean), and the fit of the data to a cosine curve (r-squared value), reflecting the circadian rhythm of heart rate. Extended Feature Generation:  Beyond daily snapshot features, the study extended these features to include data across multiple days (previous 3, 6, and 12 days), aiming to capture trends and changes over time. This method involved calculating mean values, standard deviations, and gradient coefficients for these extended periods. Data Processing and Cleaning:  The study involved handling missing data, which occasionally occurred due to participants not completing the eMoodchart or not wearing the activity tracker continuously. The analysis used only complete datasets, where no single variable was missing. In total, 130 features were processed each day, including both the basic daily features and the extended features derived from them. | Random Forest Machine Learning Algorithm:  The study employed the random forest algorithm to develop the mood prediction model. This approach involves constructing numerous decision trees during the training phase and outputs the class (mood state or episode) that is the mode of the classes output by the individual trees. Feature Engineering and Selection:  The analysis utilized 130 features processed from passive digital phenotypes, including daily and extended features across multiple days to capture trends and changes over time. Feature selection was crucial to identify the most predictive features for mood states and episodes. Training and Testing of the Prediction Model:  A supervised learning approach was taken, with the data divided into training and testing sets. The model was trained using historical data to learn the patterns and tested on future data to evaluate its predictive accuracy. The training and testing process was designed to respect the temporal nature of the data, ensuring that the model’s performance was evaluated on data not seen during training. Evaluation Metrics:  The performance of the mood prediction model was assessed using standard metrics like accuracy, sensitivity, specificity, and the Area Under the Curve (AUC) values. These metrics helped determine the model's effectiveness in predicting mood states and episodes accurately. Temporal Validation:  To account for the temporal aspect of mood prediction, the study implemented a validation strategy where the model was trained on a portion of the timeline and tested on a subsequent portion. This approach aimed to mimic real-world application and ensure the reliability of the model's predictions over time. Development of Personalized Models:  The study also explored the creation of personalized models for individual participants versus a generalized model for all participants. This comparison aimed to assess whether personalized models, trained on data from a single individual, could offer improved prediction accuracy for that individual. Analysis of Feature Importance:  An analysis was conducted to identify which features were most influential in predicting mood states and episodes, providing insights into the relationships between various digital phenotypes and mood variations. |
|  | Effectiveness of a Smartphone App With a Wearable Activity Tracker in Preventing the Recurrence of Mood Disorders: Prospective Case-Control Study | Cho, Chul-Hyun; Lee, Taek; Lee, Jung-Been; Seo, Ju Yeon; Jee, Hee-Jung; Son, Serhim; An, Hyonggin; Kim, Leen; Lee, Heon-Jeon | eMoodChart |  | FitBit, eMoodChart and CRM app (for intervention group) | Not mentioned | For analyzing circadian rhythm influences through digital phenotyping, a total of 13 fundamental features were extracted. Additionally, for each of these features, further analysis was conducted over three specified durations (with n values of 3, 6, and 12 days), focusing on extracting three key statistical measures: the mean, standard deviation, and the slope of the trend line, across these periods. This approach expanded the feature set to 130 for the purpose of model development, comprising the original 13 features along with 117 derived features (calculated as 13 basic features times 3 period types times 3 statistical measures per period). | To compare the demographic and disease-related information at the start of the study between two groups, the researchers used chi-square tests, t-tests, or Fisher's exact tests, depending on what was most suitable. To examine differences in the frequency and length of mood episodes between the CRM (Continuous Remote Monitoring) and non-CRM groups during the study, they applied a generalized linear model (GLM) analysis. This approach was chosen because the two groups were not directly comparable from the start. The GLM analysis took into account any initial differences by adjusting for variables that significantly differed at baseline, treating these as confounding factors. This analysis was conducted using SAS 9.4 software.  Additionally, to assess if the changes in daily routine compliance (DGC) values between the two groups were significantly different, the researchers used the Kolmogorov-Smirnov and Mann-Whitney U tests through the Python SciPy tool. The Kolmogorov-Smirnov test checked if the DGC data from both groups came from different distributions, while the Mann-Whitney U test determined if the median of the DGC values significantly differed between the two groups. |
|  | Relapse prediction in schizophrenia with smartphone digital phenotyping during COVID-19: a prospective, three-site, two-country, longitudinal study | Cohen, Asher; Naslund, John A.; Chang, Sarah; Nagendra, Srilakshmi; Bhan, Anant; Rozatkar, Abhijit; Thirthalli, Jagadisha; Bondre, Ameya; Tugnawat, Deepak; Reddy, Preethi V.; Dutt, Siddharth; Choudhary, Soumya; Chand, Prabhat Kumar; Patel, Vikram; Keshavan, Matcheri; Joshi, Devayani; Mehta, Urvakhsh Meherwan; Torous, John | Positive and Negative Syndrome Scale (PANSS), PHQ-9, GAD-7, SF-36, SFS, PSQI, WSS and BASIS-24 |  | mindLAMP | Not mentioned | Aggregation of Data Streams: All data streams were aggregated to a daily timescale to standardize the analysis period across different types of data, making it easier to identify patterns and anomalies over time.  Anomaly Detection Calculations: Multivariate anomaly detection was used to analyze all data streams simultaneously, allowing for the longitudinal analysis of both actively collected data (such as survey responses) and passively collected data (such as accelerometer and GPS data). Each day of data collection was associated with a p-value quantifying how anomalous that day’s data points were compared to temporally nearby data points.  Classification of Events: The study employed a specific threshold (p-value less than or equal to 0.005) to classify events as anomalies. This threshold was chosen to balance between identifying genuine anomalies and minimizing false positives, given the rarity of relapse events.  Grouping of Data Streams for Clinical Significance: Data streams were grouped by their method of collection (active, passive, or data quality) and then further by their clinical significance (symptoms, sociability, medication, sleep, home time & screen duration, and engagement). This grouping helped in analyzing the data in a clinically meaningful way.  Changepoint Detection: As supplementary experimental analysis, changepoint detection was utilized to explore whether passive data could predict symptom change. This involved using the PELT changepoint detection algorithm with an L2 cost function to identify statistical changepoints in each participant’s passive data stream. | This study utilized multivariate anomaly detection to analyze all data streams simultaneously, aiming to detect signs of relapse over time. Data streams were aggregated into daily intervals, with each day assigned a p-value indicating the level of anomaly in its data points.  Events with a p-value ≤ 0.005 were deemed formal anomalies, striking a balance between the conventional 0.05 cutoff and Bonferroni correction. Anomalies occurring within 30 days of a relapse were considered true positives.  Initially, data streams were categorized by collection method—active, passive, or data quality—and subsequently by clinical significance, encompassing symptomatology, sociability, medication, sleep, hometime & screen duration, and engagement.  The anomaly detection model was compared to a logistic regression model trained solely on active data, using demographic information, medication adherence survey scores, and psychosis symptom scores to predict relapse on a monthly basis. Summary statistics like root mean squared error, sensitivity, and specificity were computed for both models, evaluating their performance in predicting relapses within the 30-day window surrounding an event.  Permutation testing was applied post-application of the anomaly detection model to each site, aiming to ascertain if the increase in detected anomalies was statistically consistent across the three sites. This involved calculating the area under ROC curves, with false positive rates exceeding 25% disregarded due to clinical tolerance considerations. Permuting control participants allowed for testing the exchangeability of site labels and the uniformity of effect magnitude across sites.  Changepoint detection served as an additional experimental analysis, investigating the potential of passive data in predicting symptom changes. The PELT changepoint detection algorithm was employed independently for each participant's passive data, identifying statistical changepoints.  Finally, Pearson correlation coefficients were computed using the pandas DataFrame.corr function to examine the correlation between survey scores and the presence of passive data changepoints within 10 days of the respective questionnaire. Associated p-values were calculated using the scipy.stats.pearsonr method. |
|  | Wearable Technology for High-Frequency Cognitive and Mood Assessment in Major Depressive Disorder: Longitudinal Observational Study | Cormack, Francesca; McCue, Maggie; Taptiklis, Nick; Skirrow, Caroline; Glazer, Emilie; Panagopoulos, Elli; van Schaik, Tempest A; Fehnert, Ben; King, James; Barnett, Jennifer H | Spatial working memory (SWM), CANTAB rapid visual information processing (RVP), PHQ-9,PHQ0D, The University of California Los Angeles Loneliness Scale (UCLA-LS) |  | Apple Watch app, paired with participants' iPhones | the data storage solution employed involved a secure and structured approach leveraging modern cloud services. Specifically, the study utilized Amazon's Web service as the platform for data storage.  The process worked as follows:  Data Collection: Data were collected through an app installed on an Apple Watch, paired with participants' iPhones. This data included mood and cognitive assessments conducted through the app, as well as physical activity metrics such as step counts and heart rates monitored by the Apple Watch. Data Transfer: The collected data were transferred automatically from the Apple Watch and iPhone to the cloud-based storage via Wi-Fi or data roaming. This transfer process was designed to occur seamlessly, ensuring that data were uploaded continuously as long as the devices had internet connectivity. Security and Privacy: To maintain the security and privacy of the participants' data, the cloud-based system provided by Amazon's Web service implemented strict access controls. These controls ensured that participants had write access to upload their data, while study managers and researchers only had read access. This level of access control is critical in clinical and research settings to protect participant confidentiality and comply with data protection regulations. | Preparation and Cleaning of High-Frequency Data Adherence were evaluated separately for cognitive function, mood reports, and activity. Adherence to mood and cognitive assessments was defined according to the methods outlined in the clinical trials registration [31], where each day was categorized as adherent (participants completing at least one full assessment) or nonadherent (days with no data). Regarding Apple Watch activity and heart rate measures, days where participants recorded <100 steps [42,43] (n=19 observations) or where heart rate data was missing (n=6 additional observations) were excluded from analyses. There was no specified minimum adherence for participants to be included in the analyses.  The percentage of adherent days was examined separately for mood, cognitive function, and activity over the study duration (defined as the percentage of 42 days completed) and calculated for individual study weeks (weeks 1-6). Additionally, for cognitive assessments with three daily prompts, the percentage of responses to all possible assessments was examined.  Daily dprime performance was determined as the mean of all available n-back assessments within each day. Total daily mood was computed as the sum of responses across the three questions presented during each assessment. The total step count from the iPhone and Apple Watch was recorded for each day. Minimum, maximum, and mean daily heart rates were obtained from the Apple Watch.  Summary measures for daily assessments included total daily mood, daily dprime, average heart rate, and total step count. The means of all available daily assessments were calculated across the entire assessment period (6 weeks) and for individual weeks (1-6) to observe changes over time. No adjustments were made for missing data, and normality of all summary measures was assessed visually and with the Shapiro-Wilk test before further analysis.  Preparation and Cleaning of Web-Based Full-Length Assessments Data Absolute scores from validated self-report questionnaires were computed by summing responses within scales, providing summed scores for PHQ-9, PDQ-D, and UCLA-LS at each time point. To reduce multiple comparisons, overall scores from self-report questionnaires and CANTAB cognitive testing were calculated as the mean of outcome measures obtained at weeks 1, 3, and 6. This yielded overall means for SWM between errors, SWM strategy, RVP A′, and RVP median latency, as well as for self-report questionnaires (PHQ-9, PDQ-D, and UCLA-LS). Data normality was assessed visually and with the Shapiro-Wilk test before further analysis. | Multilevel Reliability Analysis: Examined using the multilevel.reliability command in the Psych package of R, considering missing data and using components of variance derived from multilevel mixed modeling. Longitudinal Mixed-Effects Modeling: Used for cognitive performance and mood assessment, examining daily performance and mood with fixed effects of study day and random effects of participants. Correlation Analysis: Performed to explore relationships between overall adherence with cognitive measures, and correlations between daily mood assessments with full-length questionnaires and CANTAB assessments to investigate concurrent validit |
|  | Cognitive bias modification for threat interpretations: using passive Mobile Sensing to detect intervention effects in daily life | Katharine E. Daniel, Sanjana Mendu, Anna Baglione, Lihua Cai, Bethany A. Teachman, Laura E. Barnes, and Mehdi Boukhechba. | Social Interaction Anxiety Scale (SIAS) | Ecological Momentary Assessment (EMA) surveys | MetricWire | Not mentioned | In the study, participants' raw GPS data were initially segmented into distinct clusters of locations using a spatiotemporal clustering algorithm. This algorithm organizes GPS points based on spatial and temporal criteria into clusters that represent where participants spent significant amounts of time. The process works as follows:  Initial Clustering: Each new GPS data point is compared to previous points. If a new point is more than 100 meters away from prior points, it is categorized into a new cluster.  Time Threshold for Significance: Clusters are only considered significant if their duration exceeds 300 seconds. Clusters not meeting this time threshold are disregarded.  Handling Data Anomalies: To mitigate errors, such as those caused by a dead phone battery where movement isn't recorded, clusters exceeding the 99th percentile duration within the dataset (24.8 hours) are excluded.  Consolidation of Clusters: Despite the initial clustering, the algorithm doesn't account for distance between significant places that may actually be the same location visited at different times. To refine this, the density-based spatial clustering algorithm (DBSCAN) is applied with a parameter (e) of 40 meters, efficiently grouping nearby significant places without losing natural variations.  Semantic Labeling: Each significant GPS cluster is then labeled with a meaningful category using the OpenStreetMap geodatabase. Clusters are matched to geographic entities within a 150m radius and categorized into broader classes like Education, Food, Health, Leisure, and more, based on their characteristics.  Manual Coding: For about 6.1% of the clusters where no matching semantic data could be found within the required distance from OpenStreetMap, manual coding is done using Google Maps to assign appropriate semantic labels. | Statistical Analysis:  Descriptive Statistics: Basic statistical measures were used to describe the data distributions and central tendencies. Linear Mixed-Effects Models: These models were employed to analyze the data, considering fixed effects of time (before and after the intervention), study conditions (intervention vs. control), and their interactions, with participants treated as random effects. This approach helped in understanding the impact of the cognitive bias modification intervention on various mobility-related outcomes. Outcome Measures Analysis:  Different outcome measures were considered, such as length of homestay, time spent at others' houses, location entropy, and circadian movement patterns. The analysis particularly focused on changes in these metrics from pre- to post-intervention to determine if the intervention had a statistically significant impact on participants' behavior as reflected through their mobility patterns.The analysis was conducted using R statistical software, employing packages such as 'lme4' for mixed-effects modeling and 'lmerTest' for computing p-values. |
|  | Behavioral and Self-reported Data Collected From Smartphones for the Assessment of Depressive and Manic Symptoms in Patients With Bipolar Disorder: Prospective Observational Study | Dominiak, Monika; Kaczmarek-Majer, Katarzyna; Antosik-Wójcińska, Anna Z; Opara, Karol R; Olwert, Anna; Radziszewska, Weronika; Hryniewicz, Olgierd; Święcicki, Łukasz; Wojnar, Marcin; Mierzejewski, Paweł | Hamilton Depression Rating Scale and the Young Mania Rating Scale |  | BDmon | Not mentioned | Data Aggregation: Statistics from phone calls and text messages were transformed into daily aggregates. This included data on the number of calls or messages, the duration of calls, and other related metrics. Aggregation helped in simplifying the raw data into more manageable and analyzable forms.  Feature Extraction from Speech: Acoustic features of the patient’s speech were extracted from daily phone calls. These features included various physical parameters of the speech, which were likely used to analyze patterns and changes potentially indicative of mood states.  Handling Missing Data: The study likely involved methods to handle missing data, especially since the completeness of the data varied. Techniques such as imputation or exclusion of incomplete records might have been used, depending on the extent and nature of the missing data.  Standardization or Normalization: Before analysis, data normalization or standardization might have been applied to ensure that the statistical analyses were not skewed by variables on different scales. This is common in studies dealing with variables like call duration and frequency.  Data Cleaning: This step would involve removing or correcting erroneous data entries that could affect the analysis. Temporal Alignment: Since the clinical assessments were less frequent than the data collection through the app, the study required aligning these two data sources. This involved extrapolating the psychiatric assessment to fit within the period for which behavioral data was available (e.g., 7 days before and 2 days after a visit).  Statistical Modeling Preparation: For the statistical analysis, the data was structured to fit into generalized linear mixed-effects models, requiring the organization of data into a format suitable for this type of analysis, including defining fixed and random effects based on the collected data. | To assess the relationship between behavioral markers and affective symptoms in bipolar disorder states, generalized linear mixed-effects models were employed, applying methods similar to those found in established research. The analysis began with mixed-effects linear regression using scores from clinical depression or mania rating scales as response variables. This approach quantified the correlation between behavioral markers, such as phone usage data, and the severity of affective symptoms. Additionally, mixed-effects logistic regression was used for binary classification to distinguish between normal mood days and days when patients experienced affective symptoms.  The generalized linear mixed-effects model incorporated fixed effects related to objective smartphone data (e.g., number of incoming calls) and random effects that accounted for variations across patients. Errors in the model were assumed to be normally distributed.  Further, logistic mixed-effects regression models evaluated the relationship between normal mood states and days with affective symptoms, coding days without symptoms as '0' and days with symptoms as '1'. This method adjusted the model to reflect the probability of an affective state on a given day based on the behavioral data.  Model assumptions were verified through residual analysis and visual inspections with quantile-quantile plots. The analyses were conducted in the R programming language using specific packages designed for mixed-effects models, which facilitated the estimation and provided diagnostics for the model's accuracy. It was important to interpret the results cautiously, especially for variables such as the daily number of calls, where the normal distribution assumption might not be valid. Log transformations were applied to address this issue and improve the interpretability of the results. The significance level for the analysis was set at 0.05. |
|  | The relationship between wearable-derived sleep features and relapse in Major Depressive Disorder | F. Matcham, E. Carr, N. Meyer, K.M. White, C. Oetzmann, D. Leightley, F. Lamers, S. Siddi, N. Cummins, P. Annas, G. de Girolamo, J.M. Haro, G. Lavelle, Q. Li, F. Lombardini, D.C. Mohr, V.A. Narayan, B.W.H.J. Penninx, M. Coromina, G. Riquelme Alacid, S.K. Simblett, R. Nica, T. Wykes, J.C. Brasen, I. Myin-Germeys, R.J.B. Dobson, A.A. Folarin, Y. Ranjan, Z. Rashid, J. Dineley, S. Vairavan, M. Hotopf, on behalf of the RADAR-CNS consortium | Clinical history, Composite International Diagnostic Interview – Short Form (CIDI-SF), Inventory of Depressive Symptomatology – Self-Report (IDS-SR) | Seasonal daylight exposure | Fitbit Charge 2 or Fitbit Charge 3 wearable devices, integrated into the RADAR-MDD | N/A | Sleep data from Fitbit devices were aggregated into four-week windows prior to each clinical assessment, requiring at least eight days of valid data per window. Features were centered and standardized, and polynomial terms were generated where appropriate to capture potential non-linear relationships. | Bayesian multivariable regression models were used—logistic regression for relapse outcomes and linear regression for symptom severity. Models included both within-person and between-person effects, adjusted for demographic, clinical, and seasonal covariates. Random intercepts accounted for repeated measures, and polynomial terms were included where non-linear effects were hypothesized. Population attributable fractions (PAFs) were calculated to estimate the potential preventive impact of modifying significant sleep features. |
|  | Behavioral activities collected through smartphones and the association with illness activity in bipolar disorder | Faurholt-Jepsen, Maria; Vinberg, Maj; Frost, Mads; Debel, Sune; Margrethe Christensen, Ellen; Bardram, Jakob E.; Kessing, Lars Vedel | Hamilton Depression Rating Scale (HDRS) and the Young Mania Rating Scale (YMRS) | mood, sleep length, activity levels | Bdmon | Not mentioned | Not mentioned | Generalized Linear Mixed-Effects Models (GLMM):  The core of the data analysis involved generalized linear mixed-effects models. These models are suitable for data that includes measurements taken from the same subjects over time, as they can account for both fixed effects and random effects. Fixed effects might include predictors like phone usage or movement data, while random effects account for variations across different patients. Specific Analytical Steps:  Linear Regression Models: Initially, mixed-effects linear regression was applied with scores on either the Hamilton Depression Rating Scale (HDRS) or the Young Mania Rating Scale (YMRS) as response variables. This helped quantify the relationships between the behavioral markers (like phone call duration, number of texts, etc.) and the severity of affective symptoms. Logistic Regression Models: For binary outcomes, such as distinguishing between an affective state and euthymia (a non-depressed, non-mania state), mixed-effects logistic regression models were used. These models are beneficial for predicting the probability of binary outcomes and handling data where responses are categorical. Handling of Covariates:  Both models incorporated various covariates, including the direct measures of smartphone use and patient movement. These variables served as predictors for the severity of depressive and manic episodes as assessed by HDRS and YMRS. Statistical Assumptions and Model Validation:  The assumptions of the models were checked with residual analysis and visually using quantile-quantile plots to ensure that the data met the necessary prerequisites for the analysis (e.g., normal distribution of residuals). Software and Tools:  All analyses were conducted using the R programming language, which is commonly used for statistical computing and graphics. Specific R packages utilized included those for mixed-effects models, likely lme4 for fitting the models and lmerTest for calculating p-values and assessing model fit. |
|  | Daily electronic self-monitoring in bipolar disorder using smartphones – the MONARCA I trial: a randomized, placebo-controlled, single-blind, parallel group trial | Faurholt-Jepsen, M.; Frost, M.; Ritz, C.; Christensen, E. M.; Jacoby, A. S.; Mikkelsen, R. L.; Knorr, U.; Bardram, J. E.; Vinberg, M.; Kessing, L. V | Hamilton Depression Rating Scale (HAMD-17) and the Young Mania Rating Scale (YMRS), Perceived Stress Scale (PSS) for subjective stress levels. Functioning Assessment Short Test (FAST) for psychosocial functioning. WHO Quality of Life - short version (WHOQOL-BREF) for quality of life assessments. Coping Inventory for Stressful Situations (CISS) for coping strategies. Major Depression Inventory (MDI) for self-rated severity of depressive symptoms. Altman Self-Rating Mania Scale (ASRM) for self-rated severity of manic symptoms. Massachusetts General Hospital Cognitive and Physical Functioning Questionnaire (MASS) for self-rated cognitive and physical functioning​​. | mood (scored from depressive to manic), sleep duration, medication adherence, activity levels, irritability, mixed mood, cognitive problems, alcohol consumption, stress levels, menstruation (for women), and individualized early warning signs | MONARCA system | Electronic data collected from smartphones were stored securely on a server at Concern IT, Capital Region, Denmark. The server was designated under I-suite number RHP-2011-03, ensuring that the data handling complied with local data protection regulations. | The data pre-processing included preparing and organizing the data for analysis, ensuring consistency and accuracy in the data entered. For feature extraction, specific indicators related to the patients' mood, sleep patterns, medication adherence, and other behaviors were compiled from the daily entries. | Intention-to-Treat Analysis: This approach included all patients who were randomized, adhering to the principle that every participant initially randomized into each group is included in the analysis, regardless of whether they completed the intervention according to the initial protocol.  Linear Mixed Models: These models were utilized to handle the primary outcomes of depressive and manic symptoms measured using the Hamilton Depression Rating Scale (HAMD-17) and Young Mania Rating Scale (YMRS). Linear mixed models are particularly useful for data that involve repeated measurements over time as they can accommodate both fixed and random effects, providing a robust framework for analyzing data with multiple observations per subject.  Logarithmic Transformation: The YMRS scores were logarithmically transformed to ensure that the data met the necessary assumptions for linear mixed modeling, such as normality of residuals. This transformation stabilizes variance and normalizes the distribution, making the data more suitable for analysis.  Back-Transformation: After the analysis, the transformed YMRS data were back-transformed to their original scale to interpret the effects in terms of the original units of measurement, which are more understandable to clinicians and researchers.  Exploratory Subgroup Analyses: Subgroup analyses were performed to investigate the effects of the intervention on specific groups of patients, such as those with or without mixed symptoms or those exhibiting specific baseline symptom severity. These analyses help in understanding how different groups might respond differently to the intervention. |
|  | Differences in mobility patterns according to machine learning models in patients with bipolar disorder and patients with unipolar disorder | Faurholt-Jepsen, Maria; Busk, Jonas; Rohani, Darius Adam; Frost, Mads; Tønning, Morten Lindberg; Bardram, Jakob Eyvind; Kessing, Lars Vede |  | Smartphone-based mood assessments: Patients actively entered their mood ratings using a smartphone application. This allowed for daily monitoring and assessment of mood states, which were important for correlating mood variations with mobility patterns and other behavioral data. | platform installed on Android smartphones | In the study, the data collected from the smartphones was securely stored at Concern IT, located in the Capital Region of Denmark. This facility provided a secure environment for handling and storing the data under a specific suite number (RHP-2011-03), ensuring compliance with data protection regulations. | Not mentioned | Mixed effects regression models were employed, incorporating both random intercepts and slopes for individual participants. The analysis encompassed 17 patients who underwent biweekly evaluations, resulting in a total of 102 clinical assessments. Average values derived from smartphone-collected data were used for the specific days that corresponded to the clinical ratings using the Hamilton Depression Rating Scale (HDRS-17) and the Young Mania Rating Scale (YMRS). The data were inputted using Epidata software, and analysis was performed with STATA (StataCorp LP, College Station, TX, USA) version 12.1. Statistical significance was established at p-values less than 0.05 using a two-tailed test. |
|  | Smartphone data as objective measures of bipolar disorder symptoms | Faurholt-Jepsen, Maria; Frost, Mads; Vinberg, Maj; Christensen, Ellen Margrethe; Bardram, Jakob E.; Kessing, Lars Vedel | Hamilton Depression Rating Scale-17 items (HDRS-17) and the Young Mania Rating Scale (YMRS) | Mood Scores: Patients self-reported their mood daily on a scale ranging from very depressive (-3) to very manic (+3). Sleep Duration: Tracked in hours each night to monitor sleep patterns. Medication Intake: Patients recorded their daily medication usage. Other self-monitored data: This included daily logging of irritability, mixed mood states, cognitive problems, alcohol consumption, stress levels, and other individual warning signs. | MONARCA system | In the study, the smartphone data collected through the MONARCA system was stored at a secure server managed by Concern IT, Capital Region, Denmark. The data storage facility was identified with an I-suite number, RHP-2011-03​​. | The study involving the MONARCA system conducted preprocessing of data mainly through the averaging of smartphone data that corresponded to the days on which clinical ratings such as the Hamilton Depression Rating Scale (HDRS-17) and Young Mania Rating Scale (YMRS) were made. These averages were then utilized for statistical analysis to examine correlations with clinical ratings. | Mixed-effect regression models with random intercepts and slopes for each participant were applied. The analysis was based on 17 patients who were evaluated every two weeks, resulting in a total of 102 clinical assessments. Average smartphone data corresponding to the days of the clinical assessments (HDRS-17 and YMRS) were used. The data were inputted using Epidata and analyzed with STATA (StataCorp LP, College Station, TX, USA) version 12.1. Statistical significance was determined by p-values less than 0.05 (two-tailed). |
|  | Smartphone sensing of social interactions in people with and without schizophrenia | Fulford, Daniel; Mote, Jasmine; Gonzalez, Rachel; Abplanalp, Samuel; Zhang, Yuting; Luckenbaugh, Jarrod; Onnela, Jukka-Pekka; Busso, Carlos; Gard, David E. | e Quality of Life Scale – Interpersonal Relations subscale (QLSIR) and UCLA Loneliness Scale (UCLA-LS | Ecological Momentary Assessment (EMA): Participants were asked to complete brief surveys multiple times a day through a smartphone application. These surveys included questions about their current social context, such as "Who are you with?" and "How many times did you talk or communicate with someone since the last prompt?" This method captures self-reported data on social interactions and the participant’s immediate social environment. | Ethica Data application | Not mentioned | Mobility: For the analysis of mobility using GPS data, a method was utilized that transforms location data into sequences of movements (flights) and stationary periods (pauses). This method includes a resampling approach to fill in missing trajectories based on the time and location of the gaps and then computes daily mobility metrics from these trajectories. Due to the high resource demands of the smartphone GPS sensor, such as memory and battery usage, continuous data collection isn't feasible, leading to significant amounts of missing data. This issue was addressed with an imputation method, which significantly reduces errors in daily mobility metrics. The GPS data were collected semi-continuously, providing sufficient data to calculate metrics for each participant daily. The presentation focused on mobility metrics that offered unique information due to high correlations among some metrics.  Speech Activity: An unsupervised system known as robust VAD (rVAD) was employed for voice activity detection (VAD) to recognize human speech in noisy environments. This was necessary as the recordings were made under uncontrolled conditions, which typical VAD models trained on clean recordings might not handle effectively. The ambient audio was processed in segments of 25 milliseconds, with a 15-millisecond overlap between segments. Speech activity was measured by calculating the proportion of frames identified as containing speech by the rVAD system relative to the total number of audio frames. To verify the accuracy of the rVAD system, a sample of the recordings was manually annotated. This involved identifying all discernible speech segments in the audio, except those from external sources like televisions. The annotations distinguished whether the speech was from the study participant or other people. A total of 122 five-minute segments from six participants were analyzed for this validation. The performance of the rVAD was assessed based on precision rate, recall rate, F1-score, and overall accuracy, focusing on the system's ability to correctly identify and classify speech frames.Ecological Momentary Assessment (EMA) Data:The responses from the EMA surveys were compiled and standardized to ensure consistency across all entries. | In the study, bivariate correlations were examined between the number of interactions reported via Ecological Momentary Assessment (EMA) and the proportion of EMA signals during which participants were alone, correlating these with mobility metrics and voice activity, analyzed separately for each group. Given the exploratory nature of this analysis, Pearson correlation magnitudes of 0.30 or greater were deemed as indicators of a moderate effect and were thus highlighted as significant findings. Additionally, correlations with p-values less than 0.05 were noted, adhering to conventional statistical practices.  A secondary aspect of the investigation focused on how standard assessments of social functioning and perceived social connection, specifically loneliness, correlated with the gathered passive metrics such as mobility and ambient audio data. These correlations were assessed separately for each group and aggregated across the study duration. The differences in the magnitudes of these correlations between the groups were further analyzed using Fisher r to Z transformations, with a two-tailed p-value of 0.05 used to determine statistically significant differences.  To ensure adherence to established norms for EMA surveys, data from participants who responded to at least 25% of EMA prompts were included in the analyses. This threshold was met by all participants involved in the study, ensuring a consistent data set for examination. |
|  | Digital Communication Biomarkers of Mood and Diagnosis in Borderline Personality Disorder, Bipolar Disorder, and Healthy Control Populations | Gillett, George; McGowan, Niall M.; Palmius, Niclas; Bilderbeck, Amy C.; Goodwin, Guy M.; Saunders, Kate E. A | Quick Inventory of Depressive Symptomatology (QIDS) & Altman Self-Rating Mania Scale (ASRM) | Remote mood assessments | True Colors system | Not mentioned | Not mentioned | The researchers conducted linear mixed-effects regression models with various communication variables as dependent variables. Participant ID was treated as a random intercept, while age, diagnosis, mood state, mood symptoms, and trait impulsivity were included as fixed effects. Interaction terms were added where necessary. Due to the predominantly female sample in the BPD group, gender couldn't be included as a fixed effect; instead, separate models were run for BD and HC cohorts with gender as a fixed effect. Euthymic state and HC were used as reference levels for mood state and diagnosis, respectively. They used the lmerTest package in R for analysis, considering p-values < 0.05 as significant. Unstandardized coefficients (B) were reported to indicate the change in communication variables for a one-unit change in the independent variable, while keeping others constant. When diagnosis was included, it was dummy-coded (0 or 1), with the coefficient representing the difference between diagnostic groups with other variables held constant. |
|  | Smartphone-Based Recognition of States and State Changes in Bipolar Disorder Patients | Grünerbl, Agnes; Muaremi, Amir; Osmani, Venet; Bahle, Gernot; Ohler, Stefan; Tröster, Gerhard; Mayora, Oscar; Haring, Christian; Lukowicz, Paul | Hamilton Depression Scale (HAMD) and the Young Mania Rating Scale (YMRS) |  | Android smartphones equipped with a custom-developed logging application | Data was stored on the smartphone's SD card. At the end of each day, participants were asked if they were comfortable with storing the day's data. If they agreed, the data was saved; otherwise, it was deleted. The saved data was then periodically copied during hospital visits and anonymized to protect patient privacy. | In the study, the initial phase involved extracting key features from phone call data and sound recordings, critical for any standard pattern recognition application. Here's how these features were processed:  Phone Call Features: The behaviors analyzed included total and individual call durations, whether the calls were incoming or outgoing, and the involvement of specific caller IDs (anonymized for privacy). Daily extracted features comprised:  Total number of phone calls made and received. Combined duration of all calls per day. Mean duration of phone calls. Variability (standard deviation) in call durations. Count of distinct caller IDs engaged. Sound Features: These were split into two categories—speech features and voice features:  Speech Features: These were focused on analyzing the dynamics of conversations, such as the length of speaking turns, the occurrence of short response utterances like "okay," "hm," and the silent intervals within the conversation. Specific daily measures included:  Average length of speaking turns and their duration. Mean count of speaking turns and short verbal responses. Variability in the duration of speaking turns. Rate of speaking and response utterances relative to total conversation length. Proportion of time spent speaking during conversations. Voice Features: Utilizing the 'openSmile' toolkit, an array of acoustic properties were extracted from the speech signals:  Energy distribution (kurtosis) of the speech. Average values of the second and third mel-frequency cepstral coefficients (MFCC). Mean value of the fourth derivative of MFCC (delta MFCC). Maximum zero-crossing rate (ZCR) and average harmonic-to-noise ratio (HNR). Standard deviation and range of fundamental frequency (F0). | Fusion of Sensor Modalities: The study initially employed separate classifiers for different sensor modalities such as phone usage data, sound data, GPS, and accelerometer data. These individual classifiers were then combined using a fusion method to leverage the strengths of each data type. The fusion process aimed to enhance the overall accuracy and reliability of state recognition by integrating insights from various behavioral indicators.  2. Classifier Development: For each sensor modality, classifiers were developed to distinguish between different states: depressive, manic, and normal. The features extracted from each modality were used to train these classifiers. Machine learning techniques, such as Support Vector Machines (SVM) or Naive Bayes, could have been employed here, though the exact methods were not specified in the document.  3. Fusion Techniques for Improved Accuracy: Different fusion techniques were experimented with to find the most effective combination of modalities:  Weighted Fusion: This method involved weighting the outputs of individual classifiers based on their confidence or the reliability of the sensor data, combining them into a unified prediction. AND and OR Fusion: These logical fusion methods combined classifier outputs where AND required all sensors to agree on a state change, and OR accepted any sensor indicating a change. These methods helped to manage the trade-off between sensitivity (recall) and specificity (precision). 4. State Change Detection: The study also focused on detecting changes in the patient's state, which is crucial for timely intervention. A separate model was developed for this purpose:  Default State Model: A baseline or "default" state model was created using data presumed to represent the patient's normal state. Change Detection Algorithm: This algorithm compared current data against the default state model. Significant deviations, measured by statistical distances like the Mahalanobis distance, were flagged as potential state changes. 5. Validation: The performance of the system in recognizing states and detecting changes was validated using standard metrics such as accuracy, precision, and recall. This was crucial to assess the effectiveness of the system and its potential clinical applicability.  6. Cross-Validation: To ensure the robustness and generalizability of the findings, cross-validation techniques were likely used. This involves partitioning the data into subsets, training the model on one subset, and validating it on another to test the model’s performance across different samples of data.  7. Performance Evaluation: The results from these analyses were then evaluated to determine how well the system recognized different mental states and detected transitions between them. High precision and recall in change detection, as mentioned, indicate that the system could reliably identify significant changes in patient states, providing a strong tool for clinical monitoring. |
|  | Testing Suicide Risk Prediction Algorithms Using Phone Measurements With Patients in Acute Mental Health Settings: Feasibility Study | Haines-Delmont, Alina; Chahal, Gurdit; Bruen, Ashley Jane; Wall, Abbie; Khan, Christina Tara; Sadashiv, Ramesh; Fearnley, David | interviews assessing mood and sleep and Columbia-Suicide Severity Rating Scale (C-SSRS) | mood logs, safety plans, and other interactions with the mobile app | Custom smartphone app, integrated with wearable technology like Fitbit and health data platforms like Apple Health kit | Not mentioned | Features were extracted from the raw data, including step counts, sleep patterns, and engagement with the app. Advanced techniques like Principal Component Analysis (PCA) were used to reduce the dimensionality of the data and select the most significant features for modeling. | Machine Learning Models: Various machine learning algorithms were tested, including K-nearest neighbors (KNN), support vector machines (SVM), and random forests. The models were trained to predict suicide risk based on the features extracted from the data. Model Validation and Testing: The models were validated and tested using techniques like k-fold cross-validation to assess their accuracy and generalizability. This method involves dividing the data into k subsets and using each subset in turn for testing the model while training on the remaining subsets. Statistical Analysis: Statistical tests, such as the F-test, were applied to compare the performance of different models to determine the most effective algorithm for predicting suicide risk. |
|  | Deriving symptom networks from digital phenotyping data in serious mental illness | Hays, Ryan; Keshavan, Matcheri; Wisniewski, Hannah; Torous, John | Patient Health Questionnaire (PHQ-9) Generalized Anxiety Disorder (GAD-7) Social Functioning Scale Pittsburgh Sleep Quality Index (PSQI) Brief Assessment of Cognition in Schizophrenia (BACS) Positive and Negative Syndrome Scale (PANSS)​​. | Trail Making A and B tests | mindLAMP | Not mentioned | Data normalization and discretization were key pre-processing steps: Normalization: Data in each domain was normalized to zero mean and unit variance across the patient cohort. This normalization was also applied using the patient group's derived means and variances to the control and validation cohorts. Discretization: The normalized daily survey results were grouped into 3-day bins. Multiple entries per day were averaged, and missing data were imputed using the mean of adjacent bins if no data was available. Survey results were categorized into "elevated" or "stable" based on whether they were equal to or greater than one standard deviation above the domain mean​​. | The analysis involved calculating transition probabilities for each domain and between various symptom states. The transitions were defined based on the movement from one state (elevated/stable) to another in subsequent time steps (3-day intervals). Statistical methods included chi-squared tests to validate the significance of the transition probabilities. These probabilities were key to understanding how different symptom states influenced each other over time, forming a dynamic network of symptom interactions​​. |
|  | Anomaly detection to predict relapse risk in schizophrenia | Henson, Philip; D'Mello, Ryan; Vaidyam, Aditya; Keshavan, Matcheri; Torous John | Patient Health Questionnaire-9 (PHQ-9), the Generalized Anxiety Disorder-7 (GAD-7), the Positive and Negative Syndrome Scale (PANSS) and Clinical Global Impressions (CGI) | Ecological Momentary Assessment (EMA) data which involved self-reported symptoms and cognition assessments | mindLAMP and Beiwe | Not mentioned | Normalization: All collected data, whether from surveys or sensor data (like GPS or accelerometer readings), was normalized for each participant. This normalization process helps in reducing bias due to individual differences in usage patterns and baseline data. Feature Creation: Features were processed and extracted from both passive and active data sources. For example, passive data included GPS locations, call/text logs, and accelerometer data, which were used to compute features like mobility metrics (e.g., time spent at home, number of places visited) and social interactions (e.g., number of calls or texts). Active data involved responses from ecological momentary assessments (EMAs), assessing various symptoms and states such as mood or anxiety. Handling Missing Data: The approach to handling missing data includes methods to ensure that the analysis remains robust despite potential gaps in data collection, which is common in long-term studies involving real-world data collection. | Statistical Analysis: The study employed statistical methods to analyze the relationships between extracted features and clinical outcomes. This included the use of machine learning techniques such as anomaly detection algorithms to identify deviations from normal patterns that might indicate a risk of relapse. Anomaly Detection: Specifically, anomaly detection was applied to normalized features to identify significant deviations from each participant’s baseline, which could indicate potential health issues or changes in condition. Clustering and Correlation Analysis: Techniques like k-means clustering were used to categorize participants based on the variance and mean of their active and passive data, offering insights into different behavioral patterns associated with schizophrenia. |
|  | Towards clinically actionable digital phenotyping targets in schizophrenia | Henson, Philip; Barnett, Ian; Keshavan, Matcheri; Torous, John | Patient Health Questionnaire-9 (PHQ-9) & Generalized Anxiety Disorder-7 (GAD-7) | self-reported data collected through ecological momentary assessments (EMAs). Participants actively provided information on their mood, anxiety levels, psychosis symptoms, sleep quality, and sociability. | mindLAMP and Beiwe | Not mentioned | Normalization and Baseline Establishment: Data was normalized to establish a baseline for each participant, which is crucial for anomaly detection and comparative analysis.  Feature Extraction: Features were extracted from both active and passive data sources. Active data included survey responses and cognitive task results. Passive data, collected with minimal user interaction, included GPS, accelerometer readings, screen on/off times, and call/text logs.  Data Cleaning: This involved handling missing data, likely through methods such as imputation or excluding periods of data loss. The document mentions assessing missingness based on sampling rates, which is critical in ensuring the quality and reliability of the dataset.  Feature Categorization and Clustering: Features from both data types were categorized and analyzed using statistical methods like k-means clustering. The clustering helped categorize subjects based on the variance and mean of the collected data, aiding in identifying patterns and correlations within the data.  Correlation Analysis: A correlation matrix was created to investigate associations between various data features using the Spearman correlation method, adjusted for multiple comparisons using False Discovery Rate (FDR) corrections. | Active data were examined in terms of average and variability, while passive data were converted into understandable metrics like time spent at home and incoming text counts, with means and variances calculated for each individual. A statistical clustering technique, commonly applied in psychiatric studies, was used to classify subjects based on the variability in both active and passive data. The study utilized k-means clustering to organize participants with schizophrenia based on the average and variance of active and passive data, followed by a comparison of these clusters. The optimal number of clusters,  k, was chosen using the Silhouette Method, employing R packages "factoextra" and "Nbclust." Furthermore, relationships between active and passive data features were explored through a correlation matrix for all data streams. These correlations were performed using the Spearman method and adjusted for multiple comparisons using the False Discovery Rate (FDR) correction. |
|  | Investigating Associations Between Screen Time and Symptomatology in Individuals With Serious Mental Illness: Longitudinal Observational Study | Henson Philip; Rodriguez-Villa Elena; Torous John | Patient Health Questionnaire-9 (PHQ-9), the Generalized Anxiety Disorder-7 | Cognitive tests like the Jewels A/B tasks adapted from classic Trails-A and Trails-B tasks, | mindLAMP and Beiwe | The collected data included both passive data (like GPS, accelerometer readings, and screen on/off times) and active data from participant responses. This data was uploaded to a Health Insurance Portability and Accountability Act (HIPAA)-compliant server every hour, ensuring that the data handling adhered to privacy and security regulations. | Data Aggregation: Raw screen time data were aggregated by day. This process involved calculating the total amount of screen time each day by summing up the intervals between screen on and screen off events.  Session and Checks Calculation: The study differentiated between overall screen time and specific user interactions, termed as "sessions" and "checks." A session time was calculated by dividing the total screen time by the number of sessions, with checks defined as quick interactions with the phone (lasting less than 15 seconds), indicative of habitual checking behavior.  Survey Data Aggregation: Surveys filled out by participants were also aggregated by day. If multiple surveys of the same type were completed on the same day, the scores were averaged to provide a daily score. This helped in simplifying the analysis and reducing the data to manageable levels for daily trend analysis.  Cognitive Test Scores: For cognitive tests such as the Jewels A and B tasks (adapted from classic Trails-A and Trails-B tasks), a single score, or “beta value,” was calculated for each session, which took into account both accuracy and error rates. This provided a comprehensive measure of cognitive performance per interaction.  Correlation Analysis Preparation: Before conducting any correlation analysis or regression modeling, the first month of screen time data was used along with baseline cognition data. The Spearman rank correlation coefficient was used for preliminary analyses, and P values were adjusted using the false discovery rate method to account for multiple comparisons. | Multivariate Multiple Linear Regression:  The study conducted multivariate multiple linear regression to explore the relationships between screen time metrics (such as number of sessions, number of checks, total screen time, and session time) and cognitive performance as measured by the Jewels B cognitive task. This analysis was conducted separately for participants with schizophrenia and healthy controls. For participants with schizophrenia, the regression revealed a significant association, indicating that variations in screen time metrics could predict changes in cognitive performance. Specification Curve Analysis (SCA):  A comprehensive Specification Curve Analysis (SCA) was performed using over 600 different specifications to examine the breadth of potential associations between screen time and various mental health symptoms (e.g., mood, anxiety, sleep, sociability). This method allowed the researchers to identify how different analytical choices (such as different regression models, covariates, and diagnostic groups) influenced the observed relationships between screen time and symptomatology. The results were presented in a way that highlighted the heterogeneity in these associations, ranging from very negative to very positive. Individual Participant Regression Analysis:  The study also included a detailed individual-level analysis, where linear model regression was performed to assess the impact of screen time on symptoms among individual participants. This analysis highlighted the variability in the effects of screen time across individuals, showcasing that the influence of screen time is highly personalized and differs significantly even among people with the same diagnosis. Handling of Data Variance and Corrections:  The Spearman rank correlation coefficient was used for preliminary analyses of the relationship between screen time and baseline cognition. To account for the multiple testing scenario, P values were adjusted using the false discovery rate method, ensuring that the findings were robust against Type I error inflation. |
|  | Digital Biomarkers of Social Anxiety Severity: Digital Phenotyping Using Passive Smartphone Sensors | Jacobson, Nicholas C.; Summers, Berta; Wilhelm, Sabine | Social Interaction Anxiety Scale (SIAS),Depression, Anxiety, and Stress Scale—Depression Scale (DASS-21),Positive Affect Negative Affect Schedule (PANAS) |  | Sensus mobile app | Not mentioned | 1. Accelerometer Data Preprocessing and Cleaning: Data were processed consistently following a previously published procedure, which likely involved removing noise, correcting for sensor misalignment, and handling missing data through techniques such as interpolation or exclusion of incomplete records. Feature Extraction: A comprehensive set of features was extracted: Distribution metrics: mean, median, mode, minimum, maximum, skewness, kurtosis, and standard deviation. Temporal dynamics: root mean square of successive differences and quantiles (from 1st to 99th). Autoregressive features: modeled using a differential time-varying effect model within a generalized additive framework, capturing complex temporal relationships. Normalization: It's typical in accelerometer data to normalize features to a standard scale, such as zero mean and unit variance, to reduce the impact of varying signal amplitudes across different devices or users. 2. Text Message Data Preprocessing and Cleaning: Given the sparser nature of text data, preprocessing likely involved structuring timestamp data and perhaps handling outliers in the timing of messages. Feature Extraction: Distribution of time differences between messages to capture interaction delays. Measures of variability in response times among different contacts, which may indicate social anxiety traits. Normalization: Features could be normalized to account for differences in messaging habits across individuals, ensuring comparability in the statistical modeling process. 3. Call Data Preprocessing and Cleaning: Similar to text data, this would involve organizing call timestamps and possibly identifying and handling missed or ignored calls appropriately. Feature Extraction: Features similar to those of text messages were extracted, focusing on call frequency, missed calls, and response times to calls. Normalization: Normalizing these features would also be crucial, especially for comparative purposes in the machine learning models. 4. General Data Handling Missing Data: It is noted that there was no missing data for the biomarkers from accelerometers, text messages, and calls. However, where missing data existed in related variables (like depression scores), multiple imputation was used. | Machine Learning Models Ensemble of Extreme Gradient Boosting Machines (XGBoost): The study used an ensemble of extreme gradient boosting machines, which are advanced machine learning algorithms composed of multiple weak tree-based learners. These models are known for their effectiveness in handling various types of data and for their robust performance in predictive accuracy. Different models within the ensemble were responsible for predicting social anxiety from different sets of features extracted from the accelerometer data, text message logs, and call logs. Cross-Validation Technique Leave-One-Out Cross-Validation (LOOCV): To ensure the models' predictive accuracy and generalizability, the study utilized leave-one-out cross-validation. This method involves using each data point in the dataset as a validation set while the rest serve as the training set. This approach is particularly useful in situations where the dataset is not very large, as it maximizes the amount of data used for training. This technique helps to prevent overfitting and provides a clear insight into how the model would perform on unseen data. Correlation and Predictive Accuracy Correlation Analysis: The effectiveness of the models was primarily evaluated by the correlation coefficient between the predicted and observed SAD symptom severity. A high correlation indicated that the models were successful in capturing the nuances of social anxiety as reflected by the passive smartphone data. Additional analyses were conducted to assess discriminant validity, comparing the strength of the correlation of predicted SAD symptom severity with other psychological states such as depression and affective states. Statistical Testing The significance of the discriminant validity was also tested to ensure that the models specifically predicted social anxiety and not just general psychological distress or other emotional states. Visualization and Interpretation Variable Importance and t-Distributed Stochastic Neighbor Embedding (t-SNE): To further analyze the predictive models, the study examined the variable importance from the model ensemble, identifying the most significant features contributing to the predictions. t-SNE plots were used to visually represent the separation and clustering of data points based on their predicted social anxiety levels, providing a visual confirmation of the model's ability to distinguish between different severity levels of social anxiety. |
|  | MoodSensing: A smartphone app for digital phenotyping and assessment of bipolar disorder | Jia-Hao Hsu, Chung-Hsien Wu, Esther Ching-Lan Lin, Po-See Chen | Weekly medical staff-administered HAM-D and YMRS interviews.  Chinese version of the MINI diagnostic interview at baseline. | Demographics: age, gender, education, employment, marital status, age of onset of illness, illness years.  App usage metrics.  Missing data thresholds.  Engagement statistics over 3 years. | MoodSensing smartphone app. | Not mentioned. | Data types categorized by frequency: - Anytime: emotional scores, multimedia entries. - Daily: mood score, wake/sleep time. - Weekly: ASRM-5 and DASS-21 self-scales.  Thresholds applied for inclusion (e.g., GPS ≥2000 pts/week, mood/sleep ≥3 days/week)  Missing data handled via zero-padding  Multimodal features extracted using pretrained models (RoBERTa, wav2vec, ResNet18)  Daily features averaged; weekly features structured into 7-day sequences | Scale score prediction using: Regression models: linear, ridge, lasso, elastic net, polynomial.  Deep learning: MLP, GRU. GRU achieved best performance: MAE = 0.84 (HAM-D), 0.22 (YMRS).  Feature importance assessed via Lasso weights.  5-fold cross-validation used for evaluation.  Multimedia and self-scale features contributed most to prediction accuracy. |
|  | Smartwatch digital phenotypes predict positive and negative symptom variation in a longitudinal monitoring study of patients with psychotic disorders | Kalisperakis, Emmanouil; Karantinos, Thomas; Lazaridi, Marina; Garyfalli, Vasiliki; Filntisis, Panagiotis P.; Zlatintsi, Athanasia; Efthymiou, Niki; Mantas, Asimakis; Mantonakis, Leonidas; Mougiakos, Theodoros; Maglogiannis, Ilias; Tsanakas, Panayotis; Maragos, Petros; Smyrnis, Nikolaos | International Physical Activity Questionnaire (IPAQ),Positive and Negative Syndrome Scale (PANSS) |  | Samsung Gear S3 smartwatch (runs on Tizen OS) | The raw data collected from the Samsung Gear S3 smartwatch were saved in comma-separated format. During charging times, the data were compressed and transferred via WiFi to a cloud server hosted on the “∼okeanos” national public infrastructure-as-a-service. | Data Pre-processing and Cleaning:  Heart Rate Data: Preprocessing involved calculating the real heart rate sequence, dropping identical consecutive values, and replacing outlying PP intervals (pulse-to-pulse intervals) using linear interpolation. For consistency, intervals containing at least 90% valid heart rate data were retained​​. Accelerometer and Gyroscope Data: Missing values in these datasets were replaced using nearest interpolation. Intervals with no more than 50 missing values were considered for feature extraction, ensuring data completeness and accuracy​​. Noise Reduction: Wavelet de-noising was applied to accelerometer and gyroscope sensor data to reduce the inherent noise and improve the reliability of the measurements​​. Feature Extraction:  Motor Activity: Total motor activity (TMA) was derived from both accelerometer and gyroscope data, calculated as the Short Time Energy of the Euclidean norm over each 5-minute window. This measure reflects the intensity of physical activity​​. Heart Rate Features: The average heart rate (HRA) and the standard deviation of PP intervals (a measure of heart rate variability, HRV) were calculated for each 5-minute interval​​. Walking and Sleep/Wake Data: Walking activity was quantified by the number of steps per minute, and sleep/wake ratios were derived daily, using data provided by the smartwatch's built-in algorithms​​. | Derivation of Smartwatch Phenotypes:  The analysis utilized smartwatch data to derive several digital phenotypes such as heart rate average (HRA), heart rate variability (HRV), total motor activity (TMA), sleep/wake ratio (SWR), and walking activity (WA). These phenotypes were quantified both as mean values and standard deviations to capture both the central tendency and variability across monthly intervals​​. Statistical Analysis:  Linear Mixed-Effects Model: The study employed linear mixed-effects models to estimate the relationship between each smartwatch phenotype (independent predictor) and psychopathology dimensions (dependent variable) as measured by the PANSS score. This approach allowed the inclusion of both fixed effects (predictors of interest) and random effects (to account for inter-subject variability and repeated measures within subjects)​​. FDR Correction: To adjust for multiple comparisons, a false discovery rate (FDR) correction was applied to the p-values, ensuring the robustness and reliability of the statistical inferences made from multiple tests​​. Modeling Confounding Factors:  Additional analyses examined the effects of various demographic, clinical, and pharmacological factors on the relationships derived from the primary models. This involved testing for interactions between these factors and the digital phenotypes in predicting the psychopathology dimensions​​. These secondary analyses helped to refine the understanding of how external variables might influence or moderate the primary relationships observed. Validation and Interpretation:  The results from these models were interpreted to determine the significance and strength of the relationships between smartwatch-derived phenotypes and changes in psychopathology. Significant predictors were highlighted, providing insights into which digital phenotypes were most strongly associated with changes in positive and negative symptoms among the participants​​. |
|  | Smartphone digital phenotyping, surveys, and cognitive assessments for global mental health: Initial data and clinical correlations from an international first episode psychosis study | Lakhtakia, Tanvi; Bondre, Ameya; Chand, Prabhat Kumar; Chaturvedi, Nirmal; Choudhary, Soumya; Currey, Danielle; Dutt, Siddharth; Khan, Azaz; Kumar, Mohit; Gupta, Snehil; Nagendra, Srilakshmi; Reddy, Preethi V.; Rozatkar, Abhijit; Scheuer, Luke; Sen, Yogendra; Shrivastava, Ritu; Singh, Rahul; Thirthalli, Jagadisha; Tugnawat, Deepak Kumar; Bhan, Anant; Naslund, John A.; Patel, Vikram; Keshavan, Matcheri; Mehta, Urvakhsh Meherwan; Torous, John | Positive and Negative Syndrome Scale (PANSS) Brief Assessment of Cognition Scale (BACS) Patient Health Questionnaire (PHQ-9) Generalized Anxiety Disorder (GAD-7) Social Functioning Scale Pittsburgh Sleep Quality Index (PSQI) |  | mindLAMP | Data Transmission: All collected data were transmitted securely from the participant's device to the study's servers. | Data Cleaning and Preprocessing:  For the participants from the Beth Israel Deaconess Medical Center (BIDMC), all preprocessing and cleaning were performed in-house at the Division of Digital Psychiatry. This included handling mindLAMP app data and other collected assessments. For participants from the Indian sites, preprocessing was conducted locally to comply with local data use agreements. This ensured that only de-identified data were shared for further analysis. Feature Extraction:  Passive Data: Features were extracted from the passive data collected, such as GPS data points. This involved assessing the quality of GPS data and generating features like "home time" and "entropy" to describe participants' daily movements. Home time calculated the hours spent at a home location per day, while entropy measured the variability in location per day. Active Data: Surveys and cognitive assessments conducted through the app were preprocessed to ensure consistency and reliability in the responses collected | Statistical Analysis:  The study employed various statistical tests to analyze the data collected through the mindLAMP app and clinical assessments. Key methods included the Kruskal-Wallis rank-sum test to assess differences across the three study sites and between-site comparisons using the Wilcoxon rank-sum test for non-parametric data. Fisher’s exact test was used for categorical demographic variables to evaluate differences in participant characteristics across sites. Correlation and Regression Analysis:  Linear regressions were utilized to explore relationships between clinical measures (like symptom severity assessed by scales such as PANSS) and data derived from the mindLAMP app (like engagement metrics and GPS data quality). The study sought to understand how app-reported symptoms and engagement correlated with clinically assessed symptoms and cognitive scores. Between-Site Comparisons:  Comparisons were made between participant demographics, symptom severity, mindLAMP engagement, and passive data quality across the sites in Boston, USA, and in India (Bhopal and Bangalore). This helped to assess the consistency and reliability of app engagement and data collection across diverse settings. Quality of Passive Data:  The analysis also focused on the quality and quantity of passive data collected (like GPS data points), comparing it across the three sites. This was crucial for evaluating the feasibility of using digital phenotyping methods globally. Domain-Specific Analysis:  For more detailed insights, domain-specific relationships were analyzed, such as how clinical assessments at baseline and follow-up compared to mindLAMP-derived EMA survey responses over 30 days. Use of R Packages:  The analysis and visualization of the data were performed using various R packages, such as “gt”, “gtsummary”, “ggpubr”, “ggpmisc”, and “flextable”. These tools were used to generate comprehensive tables and graphics that illustrated the study findings clearly. Handling Outliers:  Special attention was given to handling outliers in the data, such as participants who completed an unusually high number of activities or had unusually long tap times in cognitive games, to ensure the analysis was robust and reflective of typical participant behavior. |
|  | Prediction of impending mood episode recurrence using real-time digital phenotypes in major depression and bipolar disorders in South Korea: a prospective nationwide cohort study | Lee, Heon-Jeong; Cho, Chul-Hyun; Lee, Taek; Jeong, Jaegwon; Yeom, Ji Won; Kim, Sojeong; Jeon, Sehyun; Seo, Ju Yeon; Moon, Eunsoo; Baek, Ji Hyun; Park, Dong Yeon; Kim, Se Joo; Ha, Tae Hyon; Cha, Boseok; Kang, Hee-Ju; Ahn, Yong-Min; Lee, Yujin; Lee, Jung-Been; Kim, Leen | Face-to-face clinical interviews | Ecological momentary assessments | Fitbit Charge HR & eMoodChart | Not mentioned | Data Pre-processing and Cleaning: Missing Data Handling: Data occasionally had missing values for various reasons, such as the participant not wearing the device or technical issues like battery discharge. These missing fields were replaced with other similar complete records, which suggests the use of imputation techniques to handle incomplete data. Data Imputation: Specific details on the imputation method used were not mentioned, but typically this would involve statistical methods to estimate missing values based on available data, ensuring the dataset used for model training and testing was complete. Feature Extraction: Feature Construction: The study developed features from the raw data collected from wearable devices and smartphones. These features were likely derived from the physiological and behavioral data captured, such as steps, heart rate, and sleep patterns. Cosinor Analysis: For circadian rhythm analysis, the study applied cosinor fitting to heart rate data collected over two consecutive days. This method fits a cosine curve to time-series data to extract features related to circadian rhythms, such as the acrophase (the time of the peak of the rhythm) and the goodness of fit. Algorithmic Feature Selection: A feature selection process was utilized to identify the most relevant features that contribute to the prediction of mood episodes. This process involved selecting the top features (from an initial set of 140 features) that were most informative for the prediction models. | Model Construction: Random Forest Algorithm: The study used the Random Forest algorithm, a popular choice for classification tasks because of its robustness and ability to handle overfitting. This ensemble learning method combines multiple decision trees to improve prediction accuracy and generalizability. Model Training and Testing: Data Splitting: The dataset was likely split into training and testing sets, although explicit details about the split ratio were not provided. The training set would be used to train the prediction models, and the testing set to evaluate their performance. Cross-Validation: To ensure that the models were robust and to avoid overfitting, cross-validation might have been used, although it's not explicitly mentioned. This involves dividing the data into subsets and rotating which subset is used for testing versus training. Feature Importance and Selection: Shapley Values: The study used Shapley values to assess the importance of individual features in the prediction models. This approach helps in understanding the contribution of each feature to the prediction outcomes, providing insights into which data points are most critical for predicting mood episodes. Feature Optimization: The study highlighted the use of a feature selection process to refine the model by identifying and retaining the most predictive features. This likely involved techniques such as recursive feature elimination combined with cross-validation (ShapRFECV) to find the optimal subset of features that improves model performance. Performance Evaluation: Accuracy Metrics: The study assessed the performance of the models by calculating several metrics, including accuracy, sensitivity (true positive rate), specificity (true negative rate), and the area under the curve (AUC). These metrics are crucial for understanding how well the models can predict mood episodes, distinguishing between actual episodes and non-episodes. Model Comparison: The prediction results from the models were compared with the outcomes from face-to-face clinical interviews and assessments, providing a real-world evaluation of the model's predictive power. |
|  | Digital phenotyping in bipolar disorder: Using longitudinal Fitbit data and personalized machine learning to predict mood symptomatology | Jessica M. Lipschitz, Sidian Lin, Soroush Saghafian, Chelsea K. Pike, Katherine E. Burdick | Via REDCap:  Patient Health Questionnaire-9 (PHQ-9)  Altman Self-Rating Mania Scale (ASRM) |  | Fitbit Inspire and REDCap | Not mentioned | Participants were included if they had completed at least 24 weeks of monitoring. Exclusions: dropped out (n=2), <10 self-report assessments (n=5), no Fitbit data on >65% of days (n=4). For the 54 included participants, missing heart rate data (>1hr gaps) were imputed using random forest imputation at the minute level. Imputation was applied only to Fitbit features used for prediction, not to self-report data.  A total of 620 study days (4.3% of Fitbit data days) were imputed, ranging from 0% to 34% per participant. | BiMM forest, a longitudinal model combining random forest and Bayesian GLMM, was hypothesized to outperform others. Hence, to test this the results generated using BiMM forest were compared with six commonly used machine learning algorithms: logistic regression, regularized logistic regression, support vector machine (SVM), eXtreme Gradient Boosting (XGBoost), ran dom forest, and regularized random forest.  Classification models were implemented in R (v4.1.0) using packages including tidyverse, lubridate, glmnet, randomForest, RRF, e1071, xgboost, BiMM, pROC, and iml.  Data were split temporally: the earliest 80% of observations per participant were used for training, and the remaining for testing. Within training data (84% of total), time series split cross-validation was performed at 70%, 80%, and 90% thresholds. Hyperparameter tuning was conducted via grid search (excluding logistic regression). The best model (BiMM forest) was retrained on the full training set and evaluated on the untouched 16% test set to separate model selection from final performance testing. |
|  | Digital Phenotyping in Bipolar Disorder | Laura Orsolini , Michele Fiorani and Umberto Volpe | Hamilton Depression Rating Scale (HamD) and Young Mania Rating Scale (YMRS) |  | PRIORI application | The encrypted calls were transferred to a central secure server for processing. | Pre-processing: Included the use of a de-clipping algorithm to address the issue of audio clipping, particularly noted with the Samsung Galaxy S3 device.Feature Extraction: The audio was segmented, and 217 acoustic features related to rhythm were extracted. | Data Analysis: Utilized support vector machines (SVMs) to classify the speech into different mood states based on the extracted features. The analysis focused on detecting episodes of depression and mania using the acoustic features derived from the speech data. Evaluation Metric: The effectiveness of the classification was measured using the Area Under the Curve (AUC) metric. This metric helped assess the accuracy of the predictive model in distinguishing between different mood states (depression and mania), with separate analyses for each condition and improvements noted with specific preprocessing techniques like de-clipping and segmentation. |
|  | Machine Learning Identifies Digital Phenotyping Measures Most Relevant to Negative Symptoms in Psychotic Disorders: Implications for Clinical Trials | Narkhede, Sayli M.; Luther, Lauren; Raugh, Ian M.; Knippenberg, Anna R.; Esfahlani, Farnaz Zamani; Sayama, Hiroki; Cohen, Alex S.; Kirkpatrick, Brian; Strauss, Gregory P. | Brief Negative Symptom Scale (BNSS) & Positive and Negative Syndrome Scale (PANSS) | ecological momentary assessment (EMA) surveys to assess current psychological states | Embrace smartband and the mEMA app | Not mentioned | To determine which features were most consistently important, the study employed a comprehensive feature selection strategy involving six machine learning methods: Boruta, Recursive Feature Elimination with Cross Validation (RFECV), Logistic Regression using Statsmodels, Random Forest, H2O, and L1 Regularization. Additionally, three statistical tests were used: Chi Square, Kendall’s Rank Coefficient Method, and Select Percentile with ANOVA F-value. Feature importance within the H2O AutoML environment was assessed using H2O XGBoost and H2O GBM, which prioritized features based on their impact on cross-validation accuracy and AUC.  The study categorized features into three tiers based on their significance and frequency of selection across these methods. Top-tier features were those ranked highest across most selection methods and were statistically significant in at least one test. Second-tier features were frequently selected but less consistently than top-tier features. Third-tier features, while statistically significant, were selected less frequently in the machine learning process. This tiered system helped to prioritize features based on their relevance and consistency across different analytical methods. | Machine Learning Models:  Multiple machine learning algorithms were employed to analyze the digital phenotyping data. These included Boruta, Recursive Feature Elimination with Cross Validation (RFECV), Logistic Regression using Statsmodels, Random Forest, H2O, and L1 Regularization. For algorithms like H2O AutoML, XGBoost (eXtreme Gradient Boosting) and GBM (Gradient Boosting Machine) were used to determine the importance of features based on observed cross-validation accuracy and the area under the receiver operating characteristic curve (AUC). Statistical Analysis:  The selected features were then analyzed to measure their ability to classify diagnostic status and the presence or absence of clinically significant negative symptoms. This involved evaluating the machine learning models' accuracy, precision, recall, ROC AUC, and other relevant metrics. Validation and Testing:  The models were validated and tested using a split-sample approach, where the data was divided into a training and cross-validation dataset, and a separate test dataset to evaluate the models' performance. The performance of the models was further analyzed by comparing different classifiers, including Random Forest, K-Nearest Neighbors, and Logistic Regression, across the selected features to determine the best fitting models. |
|  | Wearable devices and mobile technologies for supporting behavioral weight loss among people with serious mental illness | Naslund John A.; Aschbrenner Kelly A.; Scherer Emily A.; McHugo Gregory J.; Marsch Lisa A.; Bartels Stephen J. | 6-Minute Walk Test (6-MWT) | weight of participants | Fitbit wearable devices and synchronized with a companion smartphone application | Export daily step count data from participants' personal Fitbit accounts into an Excel spreadsheet. | The study described a data cleaning process where days where participants recorded no steps were coded as 'missing'. This approach was taken under the assumption that if the Fitbit device was worn and functioning properly, it would be highly unlikely for it to record zero steps in a day. Missing step data typically occurred due to technical malfunctions with the Fitbit device, a dead battery, or cases where participants forgot to wear the device or temporarily lost it​​. | Linear Regression Models: The researchers employed linear regression models to analyze the association between average daily step count and changes in weight and fitness over the 6-month study duration. This analysis aimed to test the hypothesis that higher average daily step counts would be associated with greater weight loss and improved fitness.  Penalized Functional Regression Models: To explore the time-varying relationship between daily step count and weight loss or fitness improvement, the researchers used penalized functional regression models. This approach allowed them to observe and evaluate how changes in step count at different times within the study period were associated with changes in the outcome measures (weight and fitness). |
|  | Enhancing early psychosis treatment using smartphone technology: A longitudinal feasibility and validity study | Niendam, Tara A.; Tully, Laura M.; Iosif, Ana-Maria; Kumar, Divya; Nye, Kathleen E.; Denton, Jennifer C.; Zakskorn, Lauren N.; Fedechko, Taylor L.; Pierce, Katherine M. | Brief Psychiatric Rating Scale (BPRS)​​. | surveys examining mood, symptoms, medication adherence, and social behavior | Ginger.io | Stored the data collected through Ginger.io on a secure server. The participants' data, collected via the smartphone application, were anonymized to ensure privacy and confidentiality. Additionally, only aggregate data was used for analyses to further protect individual identities​​. | Cleaning of Passive Data: Passive data collected from the smartphone application included metrics like call duration and frequency, SMS frequency, and GPS-based movement data. The study ensured that the content of calls and SMS messages was not recorded, focusing only on the interaction metadata to respect privacy concerns.  Handling Missing Data: The study detailed handling missing data by annotating non-compliance or technical issues that prevented data collection. This could include periods where the device was not functioning or when the participant did not carry the phone.  Data Anonymization: To protect participant confidentiality, all data collected was anonymized before analysis. This step was crucial to maintain ethical standards and participant trust.  Feature Extraction for Analysis: For the analysis, features like daily step count, interaction frequencies, and location data were extracted and summarized for further statistical evaluation. This included calculating averages and trends over the study period. | Descriptive Statistics: Initial analyses involved using descriptive statistics to understand basic measures such as mean, median, and standard deviation of the collected data, which included survey completion rates, passive data summaries, and symptomatic assessments.  Correlation Analysis: Spearman rank correlation coefficients were used to examine relationships between baseline symptom severity, study retention, and survey completion rates. This helped in identifying any significant predictors of engagement and symptom reporting reliability.  Longitudinal Analysis: The study employed mixed-effects linear models to analyze the validity of smartphone-collected symptom data over time. This included assessing the relationship between self-reported symptoms on the smartphone app and the clinician-rated Brief Psychiatric Rating Scale (BPRS) scores. The models adjusted for time effects and participant-specific random effects, which accounted for repeated measurements on the same individuals.  Validity Testing: Specific analyses tested the validity of mobile health technology for symptom assessment by comparing smartphone-derived symptom scores with those obtained from traditional clinician-based evaluations. This was crucial to establish the reliability of mobile assessments in a clinical setting. |
|  | Detecting Bipolar Depression From Geographic Location Data | Palmius, N.; Tsanas, A.; Saunders, K. E. A.; Bilderbeck, A. C.; Geddes, J. R.; Goodwin, G. M.; De Vos, M. | Quick Inventory of Depressive Symptomatology Self-Report (QIDS-SR16) |  | smartphone application | Data was stored on the phone memory and transmitted periodically to a dedicated server. The geographic location data was anonymized relative to a random location on Earth to ensure the privacy of the participants. | Filtering Inaccurate Data: The recorded location data often contained inaccurate values, which were identified by their higher sample rate and abnormal locations. The study filtered out these inaccuracies by detecting data points that deviated significantly from a plausible trajectory or had unusually high velocities.  Data Down-Sampling: The filtered data were down-sampled to a consistent rate to standardize the analysis. The down-sampling involved using a median filter to smooth the data and reduce noise, ensuring that only the most representative data points were retained.  Data Imputation: Missing data sections were imputed under specific conditions, such as if the participant was recorded at a location close to the missing data section. This helped to fill gaps where data were likely missing due to device issues like being turned off or losing GPS signal.  Extracting Location Clusters: The study used a method similar to K-means clustering to extract distinct stationary locations from the location data. This helped to identify and categorize the significant places visited by the participants during the study period.  Feature Extraction: The study extracted various features from the preprocessed location data, such as:  Entropy and Normalized Entropy: Calculated to measure the variability in the time spent at different locations. Location Variance: Indicated the degree of movement between different locations. Home Stay: Measured the proportion of time the participant spent at their home location. Transition Time and Total Distance: Captured the dynamics of movement between locations. Number of Clusters: Represented the number of distinct locations identified. | Modeling and Prediction:  Regression Models: The study utilized regression models to estimate depressive symptom scores based on the geolocation-derived features. Specifically, linear regression models and quadratic generalized linear models with logistic link functions were applied. These models aimed to map the relationship between the location features and the weekly scores from the QIDS-SR16 questionnaire, quantifying the depressive symptomatology. Classification Analysis:  Quadratic Discriminant Analysis (QDA): This method was used to classify whether participants were experiencing depressive episodes based on their questionnaire responses. QDA allowed for modeling the data distributions of each class (depressed vs. non-depressed) with different covariance matrices, providing a quadratic decision boundary. Classification Metrics: The effectiveness of the classification was measured using various metrics, including F1 scores, classification accuracy, sensitivity, and specificity. These metrics helped to evaluate the performance of the models in accurately detecting depressive episodes from the geolocation features. Cross-Validation Techniques:  Leave-One-Participant-Out Cross-Validation: This technique was used to assess the model performance, ensuring that each participant's data was used exactly once as the test set while the remaining data formed the training set. This method helped to mitigate overfitting and provided a robust estimate of model accuracy. K-Fold Cross-Validation: Additional cross-validation schemes, such as 10-fold and 5-fold, were employed to further validate the stability and reliability of the classification models. Statistical Testing:  Model Validation: The study validated the statistical significance of the models by comparing them with a baseline model using tests like the F-test. This helped to assess whether the predictive models provided a significant improvement over simpler models. Error Metrics:  Mean Absolute Error (MAE): For regression models, MAE was calculated to quantify the average magnitude of errors between the predicted depressive scores and the actual scores reported by participants. This metric provided a clear measure of prediction accuracy. |
|  | Monitoring Changes in Depression Severity Using Wearable and Mobile Sensors | Pedrelli, Paola; Fedor, Szymon; Ghandeharioun, Asma; Howe, Esther; Ionescu, Dawn F; Bhathena, Darian; Fisher, Lauren B; Cusin, Christina; Nyer, Maren; Yeung, Albert; Sangermano, Lisa; Mischoulon, David; Alpert, Jonathan E; Picard, Rosaline W | Mini International Neuropsychiatric Interview (MINI),Hamilton Depression Rating Scale (HDRS-28) |  | E4 Empatica wristband and MovisensXS | Not mentioned | Pre-processing included improving the handling of EDA, motion, and sleep data by adding EDA features calculated during motionless intervals and normalized EDA features. Location data was down-sampled to one recording every 5 minutes, with missing latitude and longitude values extrapolated. Semantic features based on location, such as time spent at home and total distance traveled, were included. The dataset underwent dimensionality reduction and feature transformation to address potential overfitting and improve interpretability​​. | The study employed machine learning techniques, specifically average ensemble boosting and random forest, to estimate the Hamilton Depression Rating Scale (HDRS) scores from collected features. To avoid overfitting, multiple dimensionality reduction and feature transformation techniques were applied. The model's performance was evaluated under two scenarios: a user-split scenario where a subset of participants formed a test set, and a time-split scenario where early HDRS scores formed a training set, and later scores formed a test set. The Boruta algorithm was used to identify the most informative features for estimating HDRS scores​​​​. |
|  | Behavioral Indicators on a Mobile Sensing Platform Predict Clinically Validated Psychiatric Symptoms of Mood and Anxiety Disorders | Place, Skyler; Blanch-Hartigan, Danielle; Rubin, Channah; Gorrostieta, Cristina; Mead, Caroline; Kane, John; Marx, Brian P; Feast, Joshua; Deckersbach, Thilo; Alex “Sandy” Pentland; Nierenberg, Andrew; Azarbayejani, Ali | clinical interviews using the Structured Clinical Interview for DSM-IV (SCID) | audio diary entries | mobile sensing application | Secure Storage: Data collected from the mobile sensing platform were stored securely.  De-identification: The data were de-identified before storage. This involved encrypting, hashing, and using de-identified descriptors to label all collected data, which included both the identity of the participants and the identity of individuals in contact with them through the study devices. | he features derived from mobile and audio data were based on the most recent week of data before the SCID (Structured Clinical Interview for DSM Disorders) symptom assessment. This approach helped to ensure that the features used in the models were temporally close to the clinical assessments.  Data Exploration and Hypothesis-Driven Feature Creation:  A combination of hypothesis-driven and data-driven approaches was used to derive key features for symptom modeling. Descriptive statistics were applied over a week of digital trace data to create a set of features, including means, counts, and standard deviations of social interactions (like the number of texts received) and location data (such as distance traveled). Feature Reduction and Selection:  To enhance interpretability and improve prediction performance, cluster analysis was used to reduce the number of features. This involved identifying clusters of features with high within-group correlation and low between-group correlation. From each cluster, a single feature was chosen that conceptually matched a digital trace data source, such as location, social calls, or social texts. The reduced set of features aimed to prevent over-fitting and reduce inter-feature correlation, allowing for more robust modeling. Audio Data Processing:  Audio data were sampled, compressed, and converted into a waveform audio file format before computing features related to voice quality, prosody, and intonation. Audio features were aggregated over time as averages and standard deviations per recording, focusing particularly on features that could infer emotional states from vocal cues. | Model Development:  Logistic regression models were created for each symptom based on the features derived from the digital trace and audio data. These models aimed to predict the likelihood of each symptom's presence based on the extracted features. Statistical Tests and Model Validation:  Least Absolute Shrinkage and Selection Operator (LASSO): For the audio data, the LASSO technique was used to reduce the number of features. This method helps in selecting the most informative features while preventing overfitting by penalizing the absolute size of the regression coefficients. 10-Fold Cross-Validation: Both the digital trace data models and the audio data model underwent 10-fold cross-validation. This method involves splitting the data into ten parts, using nine for training and one for testing, and rotating the testing set until each part has been used. This process helps to ensure that the model performs well on unseen data and is not just fitting to the idiosyncrasies of the training set. Performance Metrics:  Area Under the Receiver Operating Characteristic Curve (AUC): The effectiveness of each model was primarily measured using the AUC. This metric provides an aggregate measure of performance across all possible classification thresholds. The AUC is particularly useful for evaluating binary classification models, as it reflects the model's ability to discriminate between the two classes (presence or absence of a symptom). Statistical Analysis of Model Performance:  The results from the cross-validation were used to select the best model for each symptom, choosing the model with the highest AUC. This approach allowed the researchers to assess not just the accuracy but also the robustness of each model. |
|  | A comparison of passive and active estimates of sleep in a cohort with schizophrenia | Staples, Patrick; Torous, John; Barnett, Ian; Carlson, Kevin; Sandoval, Luis; Keshavan, Matcheri; Onnela, Jukka Pekka | Pittsburgh Sleep Questionnaire Inventory (PSQI) and MINI (psychosis section only), PHQ-9, GAD-7, and Warning Symptoms Scale | Sleep Ecological Momentary Assessments (EMAs) | Beiwe | The data was automatically collected and transmitted securely to ensure privacy and compliance with ethical standards.  Data Retention: The collected data was stored as per local Institutional Review Board (IRB) regulations. | Features such as total sleep time, sleep onset, and waking time would be derived from patterns in the accelerometer data indicating periods when the phone was stationary was extracted. | Statistical Models:  The study utilized several statistical models to determine relationships between smartphone data and in-clinic PSQI scores. This included simple linear regression and multiple linear regression models. Cross-Validation:  Cross-validated simple linear regression was used to compare mean total PSQI scores with mean phone-based Ecological Momentary Assessment (EMA) scores. This method was crucial for testing the robustness of the predictive models and ensuring that the findings were not overfitted to the sample data. Predictive Modeling:  Linear mixed models were constructed to predict future PSQI scores using a combination of past PSQI scores, mean phone EMA scores, mean duration of sleep reported, and passive data estimates of sleep from the smartphone. This predictive modeling took into account within-subject correlation, allowing for more personalized predictions. Correlation Analysis:  The study analyzed the correlation between passive estimates of sleep duration from smartphones and self-reported sleep duration through EMAs. This was important for validating the passive data against more subjective, self-reported measures. Error Measurement:  The mean average error (MAE) was calculated to assess the accuracy of the predictions made by the smartphone data against the clinical PSQI scores. This provided a quantitative measure of how close the smartphone-based predictions were to the actual clinical assessments. Leave-One-Out Cross-Validation (LOOCV):  LOOCV was used particularly in the predictive models to estimate how well the model would perform in practice when applied to new, unseen data. This involved training the model on all but one subject and testing it on the left-out subject, repeated such that each subject was used as a test case once. |
|  | Dynamic Bidirectional Associations Between Global Positioning System Mobility and Ecological Momentary Assessment of Mood Symptoms in Mood Disorders: Prospective Cohort Study | Ting‑Yi Lee; Ching‑Hsuan Chen; I‑Ming Chen; Hsi‑Chung Chen; Chih‑Min Liu; Shu‑I Wu; Chuhsing Kate Hsiao; Po‑Hsiu Kuo | Clinical history, Ecological momentary assessment of mood symptoms, including daily self-reported depressive symptoms (Patient Health Questionnaire–9, PHQ-9) and manic symptoms (Altman Self-Rating Mania Scale, ASRM). | N/A | Beiwe | Beiwe platform uploaded an Excel file every hour, set to record 1 minute of data every 10 minutes (with upload frequency varying by smartphone type). Data were encrypted at the moment of collection and stored in a non-original latitude and longitude format, inaccessible on the phone itself. Encryption protected the transmission process, and only the research team could decrypt the data after they reached the secure server. | GPS data were processed using the Onnela Lab protocol. Data points with an accuracy of <51 were retained, and points were collapsed into 10-second intervals, calculating longitude and latitude values for each interval to reduce GPS drift effects. Distance and time between points were checked to classify movement status as moving, pause, undefined, or missing. Pauses were defined as any 2 locations within 300 seconds and <60 m apart, calculated using the Mercator projection. GPS tracking imputation was performed to address missing data and better approximate real-world distances. | ANOVA was used to compare demographic, clinical, and data collection characteristics among healthy controls, bipolar disorder and major depressive disorder groups. Polyserial correlation was computed to examine the concurrent relationships between GPS-derived mobility features and EMA mood scores across the full sample, identifying features significantly associated with mood variations. Sample size adequacy for feature selection was determined based on prior literature, confirming sufficient power. Generalized estimating equation models, with a first-order autoregressive working correlation structure, were then applied to capture dynamic associations and time-lagged relationships between mobility features and EMA mood states within the patient group over the 6-month period. A two-sided P value of <.05 was considered statistically significant for all analyses. |
|  | Associations among smartphone app-based measurements of mood, sleep and activity in bipolar disorder | Tseng, Yu-Ching; Lin, Esther Ching-Lan; Wu, Chung Hsien; Huang, Huei-Lin; Chen, Po See | Hamilton Depression Rating Scale (HAMD), Young Mania Rating Scale (YMRS), Altman Self-Rating Mania Scale (ASRM), and Depression, Anxiety, and Stress Scale (DASS-21),Pittsburgh Sleep Quality Index (PSQI)​ | self-reported data on daily mood, waking and sleeping | specifically developed smartphone app | Not mentioned | Data Cleaning:  Outliers in the sleep and activity data were identified and excluded. These outliers were defined as data points that were either 1.5 standard deviations lower than the first quartile or 1.5 standard deviations higher than the third quartile. This step was crucial for maintaining the quality and reliability of the dataset. Data Sufficiency Check:  Data points were deemed sufficient for analysis if more than half of the data was available in the analyzed time period. For example, at least 4 out of 7 days for weekly data, or 16 out of 30 days for monthly data. This criterion ensured that the data used in the analysis was representative and substantial enough to provide valid insights. Feature Extraction:  For sleep, the feature extracted was the duration between falling asleep and waking up, derived from the participants' reports on the app. For activity, the GPS data were converted to total daily distance of movement. This transformation allowed the study to quantify physical activity levels in a measurable and consistent manner. Averaging Data:  The existing data for each analyzed time period (daily, weekly, monthly) were averaged to provide consistent measures across different time frames. This averaging process was used to smooth out daily variations and focus on longer-term trends and patterns. | Correlation Analysis:  Simple correlation analyses were conducted to test associations between the app-derived data (daily mood, sleep duration, and total daily distance of movement) and the clinical measurements from scales such as HAMD, YMRS, ASRM, DASS-21, and PSQI. This step was crucial for validating the app data against established clinical measures. Repeated-Measures Correlation Analysis:  To assess the temporal associations among mood, sleep, and activity over different time frames (daily, weekly, and monthly), repeated-measures correlation analyses were performed. This statistical technique evaluates within-individual variable effects for multiple individuals over time. It helped in understanding how mood, sleep, and activity levels on one day influenced the same variables on subsequent days or longer periods. Rolling Averages for Time-Series Data:  Data were analyzed on a rolling basis where averaged data for a specific period (e.g., a week or a month) were correlated with averaged data from the subsequent period. This method allowed the study to capture and analyze trends over time, providing insights into how changes in one period affected subsequent periods. Statistical Software:  The statistical analyses were performed using the R statistical software. This included managing the data sets, conducting statistical tests, and interpreting the results to ensure rigorous and reliable findings. Significance Testing:  Significance levels were set at P < 0.05 to determine the statistical significance of the findings. This threshold helped to identify meaningful correlations and associations that were not likely to be due to random chance. |
|  | Characterizing the clinical relevance of digital phenotyping data quality with applications to a cohort with schizophrenia | Torous, John; Staples, Patrick; Barnett, Ian; Sandoval, Luis R; Keshavan, Matcheri; Onnela, Jukka-Pekka | N/A | Encompassed symptom surveys related to mood, anxiety, sleep, psychosis, and medication adherence | Beiwe | Data Retention and Security: The data collected in the study is kept on file in accordance with local Institutional Review Board (IRB) regulations.   Restricted Access: Access to the raw data is restricted as per the study protocol, primarily due to concerns about subject identifiability. | Defining Bursts and Pings Bursts: A burst was defined as a period of on-cycle time during which data was expected to be gathered according to a fixed sampling schedule. This concept was applied to manage and organize the data collection process, especially for passive data such as accelerometer and GPS data. Pings: Within each burst, specific data measurements or observations were referred to as pings. This terminology helped to distinguish individual data points collected during a burst of data collection activity. Estimating Measures of Data Quality The study emphasized the importance of estimating data quality to understand the relationship between the collected digital phenotyping data and the clinical outcomes of interest. To estimate data quality, the following measures were considered:  Daily Number of Bursts: The count of data collection periods per day provided insights into how consistently the data were being gathered. Duration of Each Burst: The length of each data collection period was analyzed to assess the completeness of the data collection efforts. Frequency of Pings: The rate at which data points were collected within each burst, measured in Hertz (Hz), helped to evaluate the granularity and detail of the collected data. | Linear Mixed Models Objective: To analyze the relationship between measures of data quality (independent variables) and future symptom-related survey responses (dependent variables). Implementation: Linear mixed models were created, incorporating data quality measures as predictors. These predictors were lagged by a fixed number of weeks to examine their influence on future clinical survey measures. Covariates: Included total accelerometer coverage, GPS coverage, the time elapsed from survey offering to viewing, the time between survey viewing and completion, and the total number of surveys completed. Outcome Variables: The average response to survey questions for each participant over time, measured on a Likert-type scale. Model Specification: The models were specified to account for within-subject variations over time, acknowledging that the same participant could have different data quality and symptom severity levels across different periods. Estimation of Data Quality Measures Bursts and Pings: The analysis quantified data quality in terms of daily number of bursts (continuous segments when data was collected), duration of each burst, and the frequency of data collection pings within each burst. Operational Definition of Data Quality: Data quality was operationally defined based on the empirical coverage of the collected data, differing by each subject and over time since enrollment in the study. Significance Testing P-values and Valence: The study reported the significance of the relationship between data quality metrics and survey outcomes both with and without correction for multiple testing. The estimated valence of each covariate was presented, indicating the direction of the relationship. Correction for Multiple Testing: The Benjamini–Hochberg–Yekutieli procedure was used to correct for multiple testing, controlling the false discovery rate and ensuring that the reported findings were robust against Type I errors. Supplementary Analysis Additional Insights: Besides the primary analysis, the study also explored the estimated number of bursts per day, average frequency per burst, average duration per burst, and average duration between bursts for accelerometer and GPS data. This supplementary analysis provided further insights into the data collection patterns within the schizophrenia cohort. |
|  | Predicting Mood Disturbance Severity with Mobile Phone Keystroke Metadata: A BiAffect Digital Phenotyping Study | Zulueta, John; Piscitello, Andrea; Rasic, Mladen; Easter, Rebecca; Babu, Pallavi; Langenecker, Scott A; McInnis, Melvin; Ajilore, Olusola; Nelson, Peter C; Ryan, Kelly; Leow, Alex | Hamilton Depression Rating Scale (HDRS) and Young Mania Rating Scale (YMRS) |  | BiAffect application | Not mentioned | Missing data were addressed through pairwise deletion, where features derived from metadata were treated as fixed effects in the modeling process. Each observation was grouped by subject, with each subject assigned their own random intercept for mood ratings. | Statistical Analysis Methods: Likelihood Ratio Testing: This method was employed to compare the fit of two statistical models—one that includes the variable of interest and one that does not—to determine if the inclusion of the variable significantly improves the model fit. In this context, it would be used to assess whether features derived from mobile phone use significantly contribute to predicting mood disturbance severity.  Multiple Linear Regression Models: These models were used to identify relationships between multiple independent variables (features collected from mobile phone use and accelerometer data) and a dependent variable (severity of mood disturbances as measured by HDRS for depression and YMRS for mania). This approach allows for the assessment of the predictive power of each feature while controlling for the influence of others.  Calculation of Marginal and Conditional R-squared Values: Marginal R-squared values give an idea of the variance in the dependent variable explained by the fixed factors alone, while conditional R-squared values consider both fixed and random factors. These metrics were likely used to evaluate the overall explanatory power of the models developed to predict mood disturbance severity. |
|  | An Observational Pilot Study using a Digital Phenotyping Approach in Patients with Major Depressive Disorder Treated with Trazodone | Čermák, Jan; Pietrucha, Slavomír; Nawka, Alexander; Lipone, Paola; Ruggieri, Alessandro; Bonelli, Annalisa; Comandini, Alessandro; Cattaneo, Agnese | PANSS (positive, negative, cognitive/disorganization, depression/anxiety, hostility);  BNSS ( asociality, anhedonia, avolition, blunted affect, and alogia);  CDSS (Depressive symptoms);  SOFAS (to evaluate the patients’ social and occupational functioning.);  BACS (cognitive battery) | Demographics (age,sex, marital status, ethnicity, education), illness duration, current antipsychotic medication status and dose | Fitbit charge 3 or 4 and android smartphone with HOPES app | Data auto-synced to HOPES app and sent to research premises for storage and analysis. | Observation window: Data collected during the 7 days following the baseline visit (excluding the baseline day). This 1-week window accounts for day-of-week effects.  Digital marker aggregation: Each digital measure was averaged across the 7-day period, except for mode_intertap_dist, which used the last valid (nonmissing) value due to its rolling window nature.  Validity threshold: A minimum of 4 valid observations within the 7-day window was required to compute a nonnull marker.  Wearable data collection rate: Calculated as the fraction of hour-long windows (out of 168 hours) with ≥1 successfully collected heart rate sample.  Smartphone data collection rate: Similarly calculated using the ambient light sensor, sampled continuously.  Data collection was passive and continuous, contingent on participant compliance: wearable correctly worn and smartphone powered on with the app running in the background. | Descriptive statistics computed for clinical measures, digital data measures, and successful data collection rates.  Mann-Whitney U test used to compare successful data collection rates between participants using study-provided phones vs personal phones.  Multiple linear regressions performed independently for each digital marker (independent variable) and clinical measure (dependent variable).  Age included as a covariate with an interaction term with the digital measure due to its known association with relapse, psychosocial functioning, and psychotic symptoms  Phone type adjustment: Binary variable indicating phone type (study vs personal) included in smartphone-based models, along with its interaction with the digital marker.  Standardization: All digital markers and clinical scales standardized across the population. Intercept term omitted.  Log(1+x) transformation applied to skewed features: distance_travelled and total_msg_sent, prior to standardization.  Multiple testing: No correction applied due to exploratory nature of the analysis.  Visualization: Heat map used to display effect sizes and visualize trends across digital markers and clinical scales. |
|  | Utility of Digital Phenotyping Based on Wrist Wearables and Smartphones in Psychosis: Observational Study | [Zixu Yang](https://pubmed.ncbi.nlm.nih.gov/?term=%22Yang%20Z%22%5BAuthor%5D), [Creighton Heaukulani](https://pubmed.ncbi.nlm.nih.gov/?term=%22Heaukulani%20C%22%5BAuthor%5D), [Amelia Sim](https://pubmed.ncbi.nlm.nih.gov/?term=%22Sim%20A%22%5BAuthor%5D), [Thisum Buddhika](https://pubmed.ncbi.nlm.nih.gov/?term=%22Buddhika%20T%22%5BAuthor%5D), [Nur Amirah Abdul Rashid](https://pubmed.ncbi.nlm.nih.gov/?term=%22Abdul%20Rashid%20NA%22%5BAuthor%5D), [Xuancong Wang](https://pubmed.ncbi.nlm.nih.gov/?term=%22Wang%20X%22%5BAuthor%5D), [Shushan Zheng](https://pubmed.ncbi.nlm.nih.gov/?term=%22Zheng%20S%22%5BAuthor%5D), [Yue Feng Quek](https://pubmed.ncbi.nlm.nih.gov/?term=%22Quek%20YF%22%5BAuthor%5D), [Sutapa Basu](https://pubmed.ncbi.nlm.nih.gov/?term=%22Basu%20S%22%5BAuthor%5D), [Kok Wei Lee](https://pubmed.ncbi.nlm.nih.gov/?term=%22Lee%20KW%22%5BAuthor%5D), [Charmaine Tang](https://pubmed.ncbi.nlm.nih.gov/?term=%22Tang%20C%22%5BAuthor%5D), [Swapna Verma](https://pubmed.ncbi.nlm.nih.gov/?term=%22Verma%20S%22%5BAuthor%5D), [Robert J T Morris](https://pubmed.ncbi.nlm.nih.gov/?term=%22Morris%20RJT%22%5BAuthor%5D), [Jimmy Lee](https://pubmed.ncbi.nlm.nih.gov/?term=%22Lee%20J%22%5BAuthor%5D) | Montgomery-Åsberg Depression Rating Scale (MADRS) | Physical examination focusing on existing signs and symptoms. Current episode of MDD (i.e., start date and recurrence). Current antidepressant therapy (i.e., start date of trazodone and dosage). Previous (only drugs used for treatment of depression) and concomitant treatments | Digital wearable device,Health Mate application, MyHealth web-based platform | Not mentioned | Not mentioned | To analyze the primary endpoint, the trend of passive parameters, scatter plots were utilized to visually represent each parameter's value per patient and study day. These plots included the mean value of all patients at each study day along with a loess regression line. Summary statistics, including the 95% confidence interval (CI) of the mean of each parameter per study day, were also presented. This analysis was conducted for the modified intention-to-treat (mITT) population. A sensitivity analysis was performed for the per-protocol (PP) population, repeating the previous analysis for both tables and plots. Additionally, all passive parameters were summarized by the average of study week, with changes from Week 1, and a similar graphical representation for the primary endpoint was used for the mITT and PP populations.  For the secondary endpoint regarding the trend of sleep score over time, scatter plots were again employed to illustrate the sleep score per patient and study day. Summary statistics per study day were also tabulated. Similar analyses were conducted by averaging the study week's data. These analyses were performed within the mITT population. The sleep score, derived directly from the tracking device, was based on four key inputs: duration, depth, regularity, and interruptions, providing a score ranging from 1 to 100, with higher scores indicating better sleep quality.  Regarding the trend of active parameters over time, scatter plots were used to visually represent each parameter per patient and study week, including all patient means and a regression line. Summary statistics per study week for each parameter were tabulated, and individual items were categorically described. A decrease in score for each active parameter corresponded to an improvement in clinical outcome. These analyses were conducted within the mITT population.  To explore the relationship between Montgomery-Åsberg Depression Rating Scale (MADRS) score and passive data measurements, Spearman's correlations were calculated between MADRS score at Baseline and each passive parameter at Week 1, and similarly for MADRS at the Follow-up visit with each passive parameter at Week 8. Scatter plots were generated to display the values of passive data and MADRS score per analyzed time point, along with the corresponding correlation value. A color panel representing the level of correlation by time point and parameter was produced. These analyses were performed within the mITT population.  A similar analysis was conducted for the relationship between MADRS score and active data measurements, including Spearman's correlations, scatter plots, and color panels between MADRS score and each active parameter per analyzed time point (Week 1/Baseline and Week 8/Follow-up).  Lastly, Spearman's correlations between each active and passive parameter per study week were provided, along with a panel of scatter plots for each combination of active and passive parameters per study week. These analyses were also performed within the mITT population. |
