# Supplementary material for: Digital phenotyping for mental health conditions: a systematic review of implementation and application
Source: Front Digit Health. 2026 Jul 9;8:1772744. doi: 10.3389/fdgth.2026.1772744 (PMC13391508; doi:10.3389/fdgth.2026.1772744)
Supplement: Supplementary file 3 [file Datasheet3.docx]

| **Study ID** | **Title** | **Authors** | **Results** | **Findings** | **Limitations** |
| --- | --- | --- | --- | --- | --- |
|  | Automatic detection of social rhythms in bipolar disorder | Abdullah, Saeed; Matthews, Mark; Frank, Ellen; Doherty, Gavin; Gay, Geri; Choudhury, Tanzeem | Average Records: Participants recorded an average of 36.5 energy instances, 46.12 mood instances, and 144.43 SRM (social rhythm metric) event entries. Distance Traveled: The average daily distance traveled was 8.34 km per participant. Sedentary vs Active Time: On average, the ratio of sedentary to active time was 2.09 per day. Human Speech Exposure: Participants were exposed to human speech for an average of 3.25 hours per day. Model Performance: The generalized model for inferring SRM scores had a root mean square error (RMSE) of 1.40, indicating reasonable performance Personalized models further reduced the RMSE to 0.92 across users. Classification Accuracy: Stable (SRM score ≥3.5) and unstable (SRM score <3.5) states were predicted with high accuracy, achieving a precision of 0.85 and recall of 0.86. | Automated Sensing Feasibility: The feasibility of using automated smartphone sensing to infer the Social Rhythm Metric (SRM) score, indicating that sensor data can effectively measure stability and rhythmicity in daily life for individuals with bipolar disorder.  Model Performance: The generalized model achieved a root-mean-square-error (RMSE) of 1.40, with personalized models improving performance to a mean RMSE of 0.92 across users, demonstrating the model's reasonable accuracy in predicting SRM scores based on smartphone data.  Classification Accuracy: The study was able to classify stable and unstable states (based on SRM scores) with high accuracy, achieving precision of 0.85 and recall of 0.86, suggesting that smartphone-based monitoring can effectively distinguish between periods of stability and instability in individuals with bipolar disorder.  High Confidence in Predictions: The confidence score associated with the model's predictions indicated that for the majority of correctly classified labels, the model had high confidence, underscoring the robustness of the predictive model against noise in sensor data. | Small Sample Size: The study was conducted with a limited number of participants (nine participants initially, with seven ultimately included in the analysis), which may not fully represent the broader population of individuals with bipolar disorder.  Short Duration: Data were collected over a relatively short period of 4 weeks A longer study period might provide more comprehensive insights into the participants' behavior and the efficacy of smartphone sensing.  Dependence on Phone Carriage: The accuracy of sensor data collection was contingent on participants carrying their phones consistently Any deviations from carrying the phone would result in gaps in the data, potentially affecting the study's outcomes.  Use of Provided Phones: Participants used smartphones provided for the study rather than their personal devices, which might influence their usual behavior and interaction with the device, potentially affecting the data collected.  State of Participants: All participants were in a euthymic (stable) mood state at the onset of the study The effectiveness of SRM tracking and sensor data accuracy during mood episodes (depressive or manic) was not assessed, which is crucial since adherence to SRM tracking can be particularly challenging during such episodes.  Potential Loss of Therapeutic Engagement: The study discussed the possibility that the automatic and passive nature of smartphone sensing might reduce the therapeutic engagement and involvement that manual tracking and self-reporting can foster in individuals managing their bipolar disorder. |
|  | Predicting Early Warning Signs of Psychotic Relapse From Passive Sensing Data: An Approach Using Encoder-Decoder Neural Networks | Adler, Daniel A; Ben-Zeev, Dror; Tseng, Vincent W-S; Kane, John M; Brian, Rachel; Campbell, Andrew T; Hauser, Marta; Scherer, Emily A; Choudhury, Tanzeem; | Within this dataset, 726 days (approximately 0.037% of the total) were identified as being within a 30-day period leading up to a psychotic relapse The best-performing model utilized a fully connected neural network autoencoder architecture, which achieved a median sensitivity of 0.25 (with an interquartile range (IQR) of 0.15 to 1.00) and a median specificity of 0.88 (IQR of 0.14 to 0.96) This represents a median increase of 108% in the identification of behavioral anomalies near the time of relapse Further analysis was conducted on the most effective model to identify behavioral features with a medium-to-large effect size in distinguishing near-relapse anomalies from days of relative health (DRH) among participants who relapsed multiple times throughout the study... | Increased Anomalies Preceding Relapse: There was a significant increase in detected behavioral anomalies within the 30 days leading up to a psychotic relapse, suggesting that changes in smartphone-derived behaviors could serve as early indicators of worsening mental health conditions.  Model Performance: The fully connected neural network autoencoder model showed promising results in distinguishing days leading up to a relapse (sensitivity of 0.25 and specificity of 0.88), indicating a substantial increase in behavioral anomalies during these periods.  Identification of Behavioral Features: A post hoc analysis of the model's predictions revealed specific behavioral features with a medium-to-large effect size that distinguished near-relapse anomalies from days of relative health These features related to aspects of social behavior, physical activity, and sleep patterns, aligning with clinical observations during relapse events.  Clinical Validation: Clinical notes collected during the original study provided qualitative validation that the behavioral features identified by the model were indeed observed by clinicians during relapse events, reinforcing the potential clinical utility of the approach. | Limited Sample Size: The study involved data from 60 participants, including 18 who experienced a relapse, which may not be representative of the broader population of individuals with schizophrenia spectrum disorders This sample size limits the ability to generalize findings across diverse patient populations. |
|  | SmartSense-D: A safety, feasibility, and acceptability pilot study of digital phenotyping in young people with major depressive disorder | [Andres Camargo](https://journals.sagepub.com/doi/10.1177/20552076251330509?url_ver=Z39.88-2003&rfr_id=ori:rid:crossref.org&rfr_dat=cr_pub%20%200pubmed#con1),  [Scott D Tagliaferri](https://journals.sagepub.com/doi/10.1177/20552076251330509?url_ver=Z39.88-2003&rfr_id=ori:rid:crossref.org&rfr_dat=cr_pub%20%200pubmed#con2), [Lianne Schmaal](https://journals.sagepub.com/doi/10.1177/20552076251330509?url_ver=Z39.88-2003&rfr_id=ori:rid:crossref.org&rfr_dat=cr_pub%20%200pubmed#con10), [Tianyi Zhang](https://journals.sagepub.com/doi/10.1177/20552076251330509?url_ver=Z39.88-2003&rfr_id=ori:rid:crossref.org&rfr_dat=cr_pub%20%200pubmed#con4), [Zamantha Munoz](https://journals.sagepub.com/doi/10.1177/20552076251330509?url_ver=Z39.88-2003&rfr_id=ori:rid:crossref.org&rfr_dat=cr_pub%20%200pubmed#con5), [Pemma Davies](https://journals.sagepub.com/doi/10.1177/20552076251330509?url_ver=Z39.88-2003&rfr_id=ori:rid:crossref.org&rfr_dat=cr_pub%20%200pubmed#con6), [Mario Alvarez-Jimenez](https://journals.sagepub.com/doi/10.1177/20552076251330509?url_ver=Z39.88-2003&rfr_id=ori:rid:crossref.org&rfr_dat=cr_pub%20%200pubmed#con7), [Niels van Berkel](https://journals.sagepub.com/doi/10.1177/20552076251330509?url_ver=Z39.88-2003&rfr_id=ori:rid:crossref.org&rfr_dat=cr_pub%20%200pubmed#con8), [Vassilis Kostakos](https://journals.sagepub.com/doi/10.1177/20552076251330509?url_ver=Z39.88-2003&rfr_id=ori:rid:crossref.org&rfr_dat=cr_pub%20%200pubmed#con9), [Lianne Schmaal](https://journals.sagepub.com/doi/10.1177/20552076251330509?url_ver=Z39.88-2003&rfr_id=ori:rid:crossref.org&rfr_dat=cr_pub%20%200pubmed#con10) | Continuous smartphone tracking and up to 10 MRI measurements have been conducted over more than 250 days for each of the five participants diagnosed with psychotic illnesses This extensive monitoring aimed to analyze the variation in MRI data in correlation with both traditional and objectively determined indicators of illness activity. | Proof-of-concept for applying a deep dynamic phenotyping approach in individuals with severe mental illness, suggesting a new paradigm for evaluating behavioral and pharmacological interventions' effects on brain structure, function, and behavior. | Not Specified |
|  | Relapse Prediction in Schizophrenia through Digital Phenotyping | Barnett, Ian; Torous, John; Staples, Patrick; Sandoval, Luis; Keshavan, Matcheri; Onnela, Jukka-Pekka | SZ and CN both demonstrated positivity offset (SZ: t = 2.13, p = 0.04; CN: t = 9.61, p < 0.001). Positivity offset difference score significantly reduced in SZ vs CN (14.97 (46.66) vs 41.72 (30.07), F(1,92) = 10.86, p = 0.001, η²p = 0.11). No group differences in negativity bias slope difference score (0.44 (1.73) vs −0.03 (0.40), F(1,92) = 3.37, p = 0.07, η²p = 0.04). Positivity intercept: 45.38 (24.86) vs 52.91 (21.06), F(1,92) = 2.47, p = 0.12; Negativity intercept: 30.41 (30.25) vs 11.19 (12.60), F(1,92) = 16.30, p < 0.001; Positivity slope: −0.15 (1.06) vs 0.06 (0.26), F(1,92) = 1.72, p = 0.19; Negativity slope: 0.29 (1.38) vs 0.03 (0.19), F(1,92) = 1.73, p = 0.19. In SZ, lower positivity offset correlated with reduced ACLB.mean (r = 0.53, p = 0.02) and greater ACLB.s.d. (r = −0.52, p = 0.02), higher BNSS avolition (r = −0.34, p = 0.03) and anhedonia (r = −0.43, p < 0.01), higher active DP avolition (r = −0.57, p < 0.001) and anhedonia (r = −0.58, p < 0.001), and lower positive event frequency (r = 0.34, p = 0.03). Lower positivity intercept correlated with BNSS anhedonia (r = −0.36, p = 0.02) and avolition (r = −0.32, p = 0.03), and with active DP anhedonia (r = −0.63, p < 0.001) and avolition (r = −0.57, p < 0.001). Lower positivity slope correlated with BNSS anhedonia (r = −0.36, p = 0.02). Higher negativity intercept correlated with active DP anhedonia (r = 0.34, p = 0.02) and avolition (r = 0.41, p = 0.01), and with ACLB.s.d. (r = 0.54, p = 0.01). | Both groups showed a positivity offset, but it was significantly reduced in SZ compared to CN, indicating diminished approach motivation in low-arousal contexts. Neither group demonstrated a negativity bias. In SZ, reduced positivity offset was linked to lower physical activity, greater sedentary behavior, more severe avolition and anhedonia, and fewer positive daily events. Lower positivity intercepts and slopes were associated with greater negative symptoms, while higher negativity intercepts related to greater anhedonia, avolition, and sedentary patterns. These results suggest that deficits in approach motivation in SZ are observable in daily life and are associated with reduced engagement in rewarding activities. | Positivity offset and negativity bias were calculated from subjective reports of arousal and emotion; absence of physiological measures (e.g. HRV, skin conductance, pupil dilation) limits multimodal interpretation. Accelerometry and geolocation measures are third-generation negative symptom assessments still undergoing validation. Sample consisted of adult outpatients with chronic, stable SZ, limiting generalizability to earlier illness stages or higher symptom severity. No clinical comparison group was included; positivity offset may be a transdiagnostic mechanism (also seen in depression). |
|  | The Positivity Offset Theory of Anhedonia in Schizophrenia: Evidence for a Deficit in Daily Life using Digital Phenotyping | Lisa A. Bartolomeo, Ian M. Raugh, Gregory P. Strauss | 22 participants with ≥14 days of complete data were included in the final analysis (total days = 598).  Typical vigorous physical activity was significantly associated with lower daily stress (β = −0.152 [−0.298, −0.007], p = 0.041) and higher daily positive affect (β = 0.526 [0.061, 0.992], p = 0.028).  Variability in daily light (β = 0.004 [0.001, 0.006], p = 0.010) and moderate physical activity (β = 0.004 [0.001, 0.007], p = 0.009) were positively associated with daily stress.  Variability in daily light (β = 0.003 [0.001, 0.006], p = 0.018) and moderate physical activity (β = 0.004 [0.001, 0.007], p = 0.011) were also positively associated with daily anxiety.  Sedentary time: No significant associations with any psychological outcome.  Sensitivity analysis: COVID-19 lockdown status attenuated the stress association for typical vigorous activity | Vigorous physical activity may have protective effects on stress and positive affect in young people with MDD.  In contrast, greater variability in light and moderate activity was linked to increased stress and anxiety, suggesting that consistency in activity patterns may be important.  The lack of association for sedentary time suggests that not all inactivity is psychologically detrimental, though the type of sedentary behavior may matter.  Authors emphasize the neurobiological benefits of vigorous activity (e.g., HPA axis regulation, neurotrophic effects), and suggest that increasing its frequency could improve affective outcomes  The findings highlight the importance of distinguishing between typical vs daily fluctuations in activity when evaluating psychological symptoms  These findings support the utility of actigraphy and EMA for capturing dynamic symptom–behavior relationships in youth depression. | Small analytic sample (n = 22) limits statistical power and generalizability.  No multiple-testing correction applied due to exploratory design.  COVID-19 lockdown status influenced results; 50% of sample enrolled during lockdown.  No tracking of treatment type or illness severity.  Sedentary behaviour not stratified by cognitive engagement (e.g., passive vs active).  No clinical intervention tested; predictive findings not linked to treatment outcomes.  BMI and other physical health covariates were not collected.  Sample skewed toward females and slightly older youth, limiting representativeness. |
|  | The Dynamic Association Between Physical Activity and Psychological Symptoms in Young People With Major Depressive Disorder: An Active and Passive Sensing Longitudinal Cohort Study | [Rosalind Baynham](https://pubmed.ncbi.nlm.nih.gov/?term=%22Baynham%20R%22%5BAuthor%5D), [Andres Camargo](https://pubmed.ncbi.nlm.nih.gov/?term=%22Camargo%20A%22%5BAuthor%5D), [Simon D'Alfonso](https://pubmed.ncbi.nlm.nih.gov/?term=%22D%27Alfonso%20S%22%5BAuthor%5D), [Tianyi Zhang](https://pubmed.ncbi.nlm.nih.gov/?term=%22Zhang%20T%22%5BAuthor%5D), [Zamantha Munoz](https://pubmed.ncbi.nlm.nih.gov/?term=%22Munoz%20Z%22%5BAuthor%5D), [Pemma Davies](https://pubmed.ncbi.nlm.nih.gov/?term=%22Davies%20P%22%5BAuthor%5D), [Mario Alvarez‐Jimenez](https://pubmed.ncbi.nlm.nih.gov/?term=%22Alvarez%E2%80%90Jimenez%20M%22%5BAuthor%5D), [Niels van Berkel](https://pubmed.ncbi.nlm.nih.gov/?term=%22van%20Berkel%20N%22%5BAuthor%5D), [Vassilis Kostakos](https://pubmed.ncbi.nlm.nih.gov/?term=%22Kostakos%20V%22%5BAuthor%5D), [Lianne Schmaal](https://pubmed.ncbi.nlm.nih.gov/?term=%22Schmaal%20L%22%5BAuthor%5D), [Scott D Tagliaferri](https://pubmed.ncbi.nlm.nih.gov/?term=%22Tagliaferri%20SD%22%5BAuthor%5D) | Self-reported delusions were significantly associated with increased odds of violent ideation (odds ratio [OR] = 3.08), damaging property (OR = 8.24), and physical aggression (OR = 12.39). Cravings for alcohol and cigarettes showed significant associations with violent ideation (alcohol OR = 5.20, cigarettes OR = 6.08), damaging property (alcohol OR = 3.71, cigarettes OR = 4.26), threatening others (alcohol OR = 3.62, cigarettes OR = 3.04), and physical aggression (alcohol OR = 6.26, cigarettes OR = 8.02). Drug cravings were associated with violent ideation (OR = 2.76) and damaging property (OR = 5.09). Decreased variability in physical activity and noisy ward conditions were associated with violent ideation (OR = .71 and OR = 2.82, respectively) Participants spent an average of 4.34 hours in the hallways, 2.11 hours near the nurses’ station, .94 hours in the kitchen, 1.24 hours in the lounge, and .61 hours in the group room. On average, participants left the inpatient unit for .79 hours over the course of the study. Fourteen participants (52%) reported at least one event of violent ideation or behavior, totaling 110 events (19% of questionnaires), with specific behaviors reported varying in frequency. | Self-reported Delusions and Violence: Delusions self-reported by the participants were significantly associated with an increased likelihood of engaging in violent ideation, damaging property, and exhibiting physical aggression.  Substance Cravings and Violence: Cravings for alcohol, cigarettes, and drugs were significantly associated with various forms of violent behavior, including violent ideation, damaging property, threatening others, and physical aggression.  Environmental Factors and Violent Ideation: Both decreased variability in physical activity and noisy ward conditions were found to be associated with an increased likelihood of violent ideation, suggesting that both individual behavioral patterns and environmental factors play roles in the propensity for violence.  Prevalence of Violent Ideation and Behaviors: A notable portion of the study's participants reported experiences of violent ideation or behavior, indicating a significant concern within this high-risk population. | Self-reported Delusions and Violence: Delusions self-reported by the participants were significantly associated with an increased likelihood of engaging in violent ideation, damaging property, and exhibiting physical aggression.  Substance Cravings and Violence: Cravings for alcohol, cigarettes, and drugs were significantly associated with various forms of violent behavior, including violent ideation, damaging property, threatening others, and physical aggression.  Environmental Factors and Violent Ideation: Both decreased variability in physical activity and noisy ward conditions were found to be associated with an increased likelihood of violent ideation, suggesting that both individual behavioral patterns and environmental factors play roles in the propensity for violence.  Prevalence of Violent Ideation and Behaviors: A notable portion of the study's participants reported experiences of violent ideation or behavior, indicating a significant concern within this high-risk population. |
|  | Use of Multimodal Technology to Identify Digital Correlates of Violence Among Inpatients With Serious Mental Illness: A Pilot Study | Ben-Zeev, Dror; Scherer, Emily A.; Brian, Rachel M.; Mistler, Lisa A.; Campbell, Andrew T.; Wang, Rui | Sensor data indicated that outpatients were typically active for 2.5 hours and near human speech for 4.4 hours per day, with variations in activity and speech patterns among individuals They covered an average daily distance of 9 miles and spent approximately 16.7 hours in the same location Outpatients used smartphones for communication, sending an average of 4.5 text messages and making 7.2 calls daily Inpatients adhered well to the study protocol, charging their phones twice daily on average... | The study found significant associations between self-reported delusions and violent ideation, damaging property, and physical aggression Cravings for alcohol and cigarettes were linked with various forms of violent ideation and behavior Decreased variability in physical activity and noisy ward conditions were also associated with violent ideation | The accuracy of data collection relied on participants carrying the smartphone If they forgot the device or lent it to others, the captured data for that time might not reflect the intended user's activity and surroundings Furthermore, location ratings couldn't be generated for times when outpatients were indoors (lacking GPS data) or out of range of WiFi or cellular networks Additionally, for inpatients, location data couldn't be collected when they left the unit equipped with Bluetooth beacons. |
|  | Mobile Behavioral Sensing for Outpatients and Inpatients With Schizophrenia | Ben-Zeev, Dror; Wang, Rui; Abdullah, Saeed; Brian, Rachel; Scherer, Emily A; Mistler, Lisa A; Hauser, Marta; Kane, John M; Campbell, Andrew; Choudhury, Tanzeem | Primary Outcomes  Safety   - No adverse events were reported by participants or clinicians throughout the 8-week study period.   Feasibility   - Protocol completion: 83% (40/48) completed the full protocol, exceeding the predefined feasibility threshold of 70%. - EMA adherence:   - During the first 3 weeks, 72% (26/36) completed ≥65% of EMA surveys (below the 80% target).   - Over the full 6-week EMA period, 61% (22/36) met the ≥65% completion threshold. - Actigraphy adherence: - 95% (38/40) provided actigraphy data. - 56% (20/38) had valid data for ≥39 days (70% of 56-day period). - Passive sensing adherence: - 81% (22/27) provided ≥39 days of valid passive sensing data. - Coverage varied by sensor:   - Location: 43.1 days (78.4%)   - Unlock duration: 43.4 days (79%)   - Social media: 34.8 days (63.3%)   - Communication: 21.1 days (38.4%)   - Inter-key delay: 32.8 days (59.6%)  **Acceptability:**  - 83.1% of participants agreed the app was usable and comfortable. - 79.8% reported comfort with passive sensing features. - 83.3% disagreed that the app made them feel upset; 61.1% disagreed with privacy concerns.   Secondary Outcomes  Associations with Depressive Symptoms (QIDS Scores)  Ecological Momentary Assessment (EMA)  Positive mood ratings were significantly associated with lower QIDS scores:   - β = −2.66 [95% CI: −3.98, −1.34]; FDR-adjusted p = 0.003 - No significant associations were found for negative mood, mood variability, or other EMA-derived features after FDR correction.   Actigraphy Measures   - Light and moderate physical activity showed initial associations with QIDS scores:   - Light: β = 0.83 [0.09, 1.58]; p = 0.029   - Moderate: β = 1.63 [0.07, 3.18]; p = 0.041   - However, both became non-significant after FDR correction (adjusted p = 0.244). - Sleep regularity showed a trend toward significance (β = −0.09; p = 0.073), but did not survive correction.   Passive Sensing Measures   - No passive sensing features (e.g., unlock duration, social media use, location entropy) were significantly associated with QIDS scores after FDR correction.   Sensor-to-Sensor Correlations   - Significant correlations observed:   - Location entropy ↔ location transitions: r = 0.65, p = 0.009   - Unlock duration ↔ social media use: r = 0.58, p = 0.039   - Sleep regularity ↔ light physical activity: r = −0.54, p = 0.006 | The authors conclude that digital phenotyping via active (EMA) and passive (smartphone sensors, actigraphy) methods were both safe and acceptable in a clinical youth population with moderate-to-severe MDD.  The inverse relationship between positive mood ratings and QIDS scores reinforces the clinical relevance of EMA-derived affective data.  Sleep regularity and physical activity measures showed moderate correlations, with higher activity linked to more stable sleep patterns. Passive sensing features such as call volume, location entropy, and screen unlock duration were internally correlated, but did not show significant associations with depressive symptoms or mood ratings after FDR correction.  However, feasibility was constrained by technical limitations of the AWARE-Light app and inconsistent device adherence, particularly for actigraphy.  These challenges mirror prior studies that report declining sensor data quality over extended durations, underscoring the need for real-time data quality monitoring and adaptive protocols. | Small sample size (n = 40) limits generalizability to broader populations.  23% of screened participants used iPhones, which were incompatible with the AWARE-Light app—restricting feasibility and applicability across platforms.  EMA survey was custom-built for this study and had not been previously validated, despite item derivation from established scales (PSQI, DASS, PSWQ, RRS, UCLA Loneliness Scale).  Passive sensing data were aggregated over 8 weeks, which may obscure dynamic temporal patterns and limit insight into symptom trajectories.  Technical issues with the AWARE-Light app led to missing data and participant dropout; 7 withdrew due to persistent app failures.  Passive sensing completeness varied widely across features; communication and inter-key delay had the lowest coverage.  No clinical intervention was tested; predictive findings were not linked to treatment decisions or outcomes. |
|  | Using wearable technology to detect the autonomic signature of illness severity in schizophrenia | Cella Matteo a, Okruszek Łukasz, Lawrence Megan a, Zarlenga Valerio a, He Zhimin a, Wykes Til | Acceptability: Both groups, people with schizophrenia and controls, rated the usability of the mHealth device favorably It was found to be a feasible method for assessing autonomic activity without disrupting daily life. Physiological Data Validity: The heart rate variability (HRV) parameters showed strong and significant correlations with each other, validating the reliability of the measurements. Group Differences: The study found significant differences in autonomic responses: People with schizophrenia showed lower levels of HRV (Standard Deviation of NN intervals [SDNN]: 102 ± 34, Root Mean Square of Successive Differences [RMSSD]: 70 ± 25) compared to controls (SDNN: 129 ± 44, RMSSD: 90 ± 27). Electrodermal activity (EDA) did not show significant differences between the groups. Movement levels were significantly lower in the schizophrenia group compared to controls. Correlations with Clinical/Functional Measures: HRV measures were significantly associated with clinical symptoms in the schizophrenia group Lower HRV correlated with more severe positive symptoms. Movement levels negatively correlated with the severity of negative symptoms, indicating that decreased physical activity is associated with more severe negative symptoms. | High Acceptability: The mHealth device was rated highly acceptable by both patients with schizophrenia and control participants, indicating its potential for regular use in monitoring health without significant disruption to daily life. Validated Physiological Measurements: The device provided reliable measurements of autonomic activity, with strong inter-correlations between heart rate variability (HRV) parameters. Significant Autonomic Differences: Individuals with schizophrenia exhibited lower levels of HRV and movement compared to control participants, suggesting impaired autonomic regulation and reduced physical activity. Association with Clinical Symptoms: Lower HRV was associated with more severe positive symptoms of schizophrenia Moreover, reduced movement was linked to more severe negative symptoms, indicating a relationship between physical activity levels and symptom severity. Potential for Monitoring and Intervention: The findings support the use of wearable technology in monitoring and potentially intervening in the management of schizophrenia, particularly for assessing illness severity and preventing relapses. | Medication Effects: The study did not exclude participants based on their medication intake, which could affect autonomic functions Although the analysis controlled for medication levels, the impact of specific medications on physiological parameters was not fully isolated. Sample Size and Diversity: The study involved a limited number of participants, which might affect the generalizability of the findings The participant pool was also not diverse in terms of demographic characteristics, which might limit the applicability of the results to different populations. Short Recording Period: The use of an 8-hour recording period for collecting data might not fully capture the variations in autonomic activity over longer periods, potentially missing significant fluctuations related to different daily activities or stressors. Activity Monitoring: The study did not use self-reported methods to monitor participants' activities, relying solely on device measurements This could lead to discrepancies in understanding the context of physiological responses. Potential Influence of Tobacco Use: Tobacco use, which can influence autonomic function, was not controlled for in the study, potentially confounding the results related to autonomic measures. |
|  | Mood Prediction of Patients With Mood Disorders by Machine Learning Using Passive Digital Phenotypes Based on the Circadian Rhythm: Prospective Observational Cohort Study | Cho, Chul-Hyun; Lee, Taek; Kim, Min-Gwan; In, Hoh Peter; Kim, Leen; Lee, Heon-Jeong | The prediction accuracies for mood state over the following three days across all participants, those with major depressive disorder (MDD), bipolar disorder type 1 (BD I), and bipolar disorder type 2 (BD II) were 65%, 65%, 64%, and 65%, respectively The corresponding area under the curve (AUC) values were 0.7, 0.69, 0.67, and 0.67.  For predicting mood episodes, the accuracies for all participants for no episode (NE), depressive episode (DE), manic episode (ME), and hypomanic episode (HME) were 85.3%, 87%, 94%, and 91.2%, with AUC values of 0.87, 0.87, 0.958, and 0.912, respectively.  Specifically, in BD II patients, the model showed a notably balanced prediction accuracy, achieving 82.6% for NE, 74.4% for DE, and 87.5% for HME, with AUC values of 0.919, 0.868, and 0.949, respectively. | The study on predicting mood states in patients with mood disorders through machine learning and passive digital phenotypes found that the developed algorithm could moderately accurately predict mood states and episodes in the near future The algorithm's performance varied slightly among patients with major depressive disorder and bipolar disorder types I and II, but it remained generally effective across these groups The prediction model was particularly noteworthy for its ability to distinguish between different mood episodes, including no episode, depressive episodes, manic episodes, and hypomanic episodes, with a good degree of accuracy Moreover, the model showed a distinctively balanced prediction accuracy for patients with bipolar disorder type II, indicating its potential for tailored applications in specific subgroups of mood disorders. | Absence of Intrinsic and Preemptive Assessments: The study did not include more intrinsic and preemptive genetic and biological assessments related to circadian rhythms, which could have required a more complex study design and analysis methods.  Variable Prediction Capabilities: In some cases, the prediction capabilities were not high The main goal was not to diagnose mood disorders but to predict mood states or episodes in already diagnosed patients to enhance prognosis through self-monitoring and self-care.  Method of Analyzing Mood States: High absolute mood scores (HAMS) and low absolute mood scores (LAMS) were applied separately according to the absolute score of mood This approach might not accurately reflect the mood state as it did not apply high and low mood scores correctly The accuracy of daily mood scores could not be verified as easily as mood episodes due to potential variations in individual characteristics and tendencies.  Generalizability of the Model Performance: The study reported model performance results for individuals from the collected dataset only, leaving uncertain how well these results would generalize to a new population... |
|  | Effectiveness of a Smartphone App With a Wearable Activity Tracker in Preventing the Recurrence of Mood Disorders: Prospective Case-Control Study | Cho, Chul-Hyun; Lee, Taek; Lee, Jung-Been; Seo, Ju Yeon; Jee, Hee-Jung; Son, Serhim; An, Hyonggin; Kim, Leen; Lee, Heon-Jeon | The study found significant differences in the recurrence of mood episodes between the CRM group and the Non-CRM group The CRM group experienced substantially fewer and shorter depressive, manic/hypomanic, and overall mood episodes Specifically, in univariable analysis, the CRM group saw a 60.7% reduction in total depressive episodes annually, 48.5% shorter depressive episodes, 85.7% shorter manic/hypomanic episodes, 66.4% fewer total mood episodes, and 63% shorter total mood episodes After adjusting for multiple variables in the multivariable analysis, these improvements were even more pronounced: 96.7% fewer total depressive episodes annually, 99.5% shorter depressive episodes, 96.1% shorter manic/hypomanic episodes, 97.4% fewer total mood episodes, and 98.9% shorter total mood episodes.  The study also evaluated the effectiveness of a feedback system on behavioral changes, revealing significant positive changes in behaviors like CR amplitude, light exposure during daytime, and daytime steps, following warning alert feedback... | The CRM group experienced substantially fewer and shorter depressive, manic/hypomanic, and overall mood episodes compared to the non-CRM group, indicating the effectiveness of the intervention in reducing the frequency and duration of mood episodes. Significant positive behavioral changes were observed in the CRM group after receiving warning alert feedback, particularly in activities such as CR amplitude, light exposure during daytime, and steps during daytime These changes suggest an improvement in daily activity levels and behaviors following the intervention. The study also found that the CRM group maintained a consistent rate of Fitbit wearing over time, contrasting with the non-CRM group, which showed a decline in wearing rate This suggests higher engagement and compliance with the intervention among the CRM group. | Generalizability: The study's findings may have limited generalizability due to the specific characteristics of the study population and the intervention's design This means the results might not apply to all individuals with mood disorders or in different demographic or clinical settings.  Self-Reported Measures: The reliance on self-reported measures for some outcomes can introduce bias, as these measures are subject to participants' interpretations, memory recall, and willingness to report accurately.  Short Duration: The relatively short duration of the study might not capture the long-term effectiveness and sustainability of the intervention's benefits Longer follow-up periods would be necessary to assess whether the improvements are maintained over time.  Lack of Blinding: The absence of blinding among participants and researchers could introduce bias Participants who are aware of their group assignment may change their behavior simply because they know they are being observed (Hawthorne effect), and researchers' knowledge of participants' group assignments might influence their interpretations of the data. |
|  | Relapse prediction in schizophrenia with smartphone digital phenotyping during COVID-19: a prospective, three-site, two-country, longitudinal study | Cohen, Asher; Naslund, John A.; Chang, Sarah; Nagendra, Srilakshmi; Bhan, Anant; Rozatkar, Abhijit; Thirthalli, Jagadisha; Bondre, Ameya; Tugnawat, Deepak; Reddy, Preethi V.; Dutt, Siddharth; Choudhary, Soumya; Chand, Prabhat Kumar; Patel, Vikram; Keshavan, Matcheri; Joshi, Devayani; Mehta, Urvakhsh Meherwan; Torous, John | Anomalies were 2.12 times more frequent in the month preceding a relapse and 2.78 times more frequent in the month preceding and following a relapse compared to intervals without relapses The anomaly detection model that incorporated passive data was found to be a better predictor of relapse than a naive model that utilized only survey data Specifically, the anomaly detection analyses revealed 188 significant anomalies at p=0.005, with 13 anomalies (6.9%) being true positives.  In terms of relapses, 20 participants experienced clinical relapses prior to August 1st, 2022 Seventeen participants had one relapse, while three participants had two relapses... | The main findings of the study demonstrated a significant association between the occurrence of statistically significant data anomalies, detected through digital phenotyping using the mindLAMP smartphone app, and the relapse events in schizophrenia patients across multiple sites in the United States and India Anomalies in the collected data were more frequent around the time of a clinical relapse, indicating that the anomaly detection model incorporating passive data from smartphones could predict schizophrenia relapse more effectively than models based only on survey data | Relapse Data Collection: All relapse data was obtained from clinical interviews and medical records There is a possibility that some participants may have experienced relapses between interviews that were not reported when asked.  Impact of COVID-19: The study was conducted during the COVID-19 pandemic, which could have influenced participants' behavior and smartphone use, potentially affecting the generalizability of the passive data results The response to COVID-19 varied between the United States and India, as well as culturally and environmentally between all sites, which might have impacted the sites and participants differently.  Data Quality: Various factors affected data quality For active data, participants might have ignored reminders to complete surveys or turned off app notifications... |
|  | Wearable Technology for High-Frequency Cognitive and Mood Assessment in Major Depressive Disorder: Longitudinal Observational Study | Cormack, Francesca; McCue, Maggie; Taptiklis, Nick; Skirrow, Caroline; Glazer, Emilie; Panagopoulos, Elli; van Schaik, Tempest A; Fehnert, Ben; King, James; Barnett, Jennifer H | Adherence Throughout the study, full adherence was observed in 21 participants for cognitive assessments, 15 for mood assessments, and 13 for activity assessments Technical issues on the final day affected the evening session's adherence However, statistical analysis indicated stable adherence over time and no association between the severity of self-reported depressive symptoms and adherence levels Adherence was not significantly correlated with cognitive measures at the beginning of the study (maximum rho=0.15, P=.44).  Participants completed an average of 86.8% of all possible n-back tasks, with lower response rates in the morning (84%) compared to the afternoon (87%) and evening (89%)... | High Adherence: The study achieved high adherence rates with 21 out of 30 participants maintaining full adherence for cognitive assessments over the study period This suggests that the wearable technology was well-accepted and feasible for continuous monitoring.  Cognitive Performance: Cognitive assessments indicated an initial rapid improvement in performance that plateaued over time This was observed in the n-back task performance, which did not show a significant relationship with the number of tasks completed, indicating that performance improvements might plateau after initial gains.  Mood Assessment Reliability: The mood assessment tools showed high reliability, with high between-person reliability and moderate to high within-person generalizability This suggests that the mood assessments were consistent and reliable over the study period.  Improvement in Mood Over Time: Multilevel analysis of mood scores revealed a modest overall linear improvement in mood across the study duration... | Sample Size and Generalizability: The study involved a relatively small sample size of 30 participants, which may limit the generalizability of the findings to a broader population of individuals with major depressive disorder.  Technical Issues: There were technical problems that affected data collection, particularly on the final day of the study, which impacted the adherence rates and potentially the completeness of the data.  Device Discrepancies: Differences in activity data recorded by the Apple Watch and the iPhone highlighted inconsistencies that could affect the reliability of physical activity measurements.  Heart Rate Measurement Accuracy: Implausible heart rate measurements suggested limitations in the accuracy of the wearable technology's physiological sensors, which could impact studies relying on such data for health monitoring.  Potential Bias from Self-Selection: The participants who chose to enroll in the study might have been more technology-savvy or motivated, which could introduce bias in terms of adherence and engagement with the wearable technology.  Lack of Clinical Validation: The study mainly assessed feasibility and validity based on adherence and reliability metrics rather than clinical outcomes, so the clinical implications of the findings remain uncertain. |
|  | Cognitive bias modification for threat interpretations: using passive Mobile Sensing to detect intervention effects in daily life | Katharine E. Daniel, Sanjana Mendu, Anna Baglione, Lihua Cai, Bethany A. Teachman, Laura E. Barnes, and Mehdi Boukhechba. | Length of Homestay Weekday Days—Concerning Hypothesis 1a, a significant change in the proportion of time spent at home between 6am and 4pm on Mondays through Fridays was observed for participants in the EMA-only condition compared to the CBM-I condition over the study duration Unexpectedly, participants in the EMA-only condition showed a larger decrease in the proportion of time spent at home during weekday days following the intervention compared to those in the CBM-I only condition On average, participants spent 2.45 hours at home each weekday between 6am and 4pm.  Weekday Evenings—Regarding Hypothesis 1b, a significant main effect of time was found for differences in the proportion of time spent at home on Mondays, Tuesdays, Wednesdays, and Thursdays from 4pm to 12am during the final two weeks compared to the first two weeks of monitoring All participants spent less time at home during weekday evenings in the final two weeks of the study compared to the first two weeks... | Length of Homestay (Weekday Days): A significant change was observed in the proportion of time spent at home between 6 am and 4 pm on Mondays through Fridays for participants in the EMA-only condition relative to the CBM-I condition over the course of the study Unexpectedly, participants in the EMA-only condition showed a larger decrease in the proportion of time they spent at home compared to other places during weekday days following the intervention compared to individuals in the CBM-I only condition​​.  General Observations: All participants visited fewer locations in the final two weeks of the study compared to the first two weeks No main effect for condition or time-by-condition effect was observed for location visits or time-based entropy of locations, indicating that the amount of time participants spent across different locations was comparably stable over time for both groups​​.  Significance of Findings: Despite null results for the specific intervention, the study provides a robust analytical framework for assessing intervention effects in daily life using passively-collected GPS data This approach advances the understanding of behavior in psychological research and leverages smartphone-enabled GPS data for predicting state anxiety in daily life... | Not Specified |
|  | Behavioral and Self-reported Data Collected From Smartphones for the Assessment of Depressive and Manic Symptoms in Patients With Bipolar Disorder: Prospective Observational Study | Dominiak, Monika; Kaczmarek-Majer, Katarzyna; Antosik-Wójcińska, Anna Z; Opara, Karol R; Olwert, Anna; Radziszewska, Weronika; Hryniewicz, Olgierd; Święcicki, Łukasz; Wojnar, Marcin; Mierzejewski, Paweł | Phone Call Behavior:  Depressed patients made phone calls less frequently than euthymic patients, with the frequency of incoming answered calls being significantly lower during depressive states compared to euthymia (β = -0.064, P = 0.01). The number of missed incoming calls was higher during depressive states and increased as depressive symptoms intensified (β = 4.431, P < 0.001; β = 4.861, P < 0.001). Manic State Phone Call Behavior:  The fraction of outgoing calls was higher in manic states (β = 2.73, P = 0.03). The fraction of missed calls was higher in manic/mixed states compared to the euthymic state (β = 3.53, P = 0.01) and positively correlated with the severity of symptoms (β = 2.991, P = 0.02). Call Duration Variability:  The variability of the duration of outgoing calls was higher in manic/mixed states (β = 0.0012, P = 0.045) and positively correlated with the severity of symptoms (β = 0.0017, P = 0.02). Text Messaging Behavior:  The number and length of sent text messages were higher in manic/mixed states compared to the euthymic state (β = 0.031, P = 0.01; β = 0.015, P = 0.01, respectively) and positively correlated with the severity of manic symptoms (β = 0.116, P < 0.001; β = 0.022, P < 0.001, respectively). Self-assessment of Mood:  Self-assessment of mood was lower in depressive states (β = -1.452, P < 0.001) and higher in manic states (β = 0.509, P < 0.001). | Correlation with Clinical Assessments:  Behavioral data collected from smartphones showed a significant correlation with clinically rated symptoms on both the Hamilton Depression Rating Scale (HDRS) and the Young Mania Rating Scale (YMRS) This suggests that smartphone data can serve as a reliable indicator of mood states in bipolar disorder. Behavioral Markers:  Specific behavioral markers derived from phone usage patterns were identified as indicative of different mood states: Decreased phone call activity and increased missed calls were associated with depressive states. Increased outgoing call activity and text messaging were associated with manic or mixed states. Predictive Value:  The study confirmed that these behavioral markers have predictive value in distinguishing between different affective states such as euthymia, depression, and mania/mixed states | Sample Size and Dropout Rate:  The initial target was to enroll 100 participants, but only 84 were recruited, and after the initial psychiatric assessment, 20 patients dropped out This reduced the final sample size to 51 participants for analysis, affecting the statistical power and generalizability of the findings. Smartphone Platform Limitation:  The BDmon app used for data collection was compatible only with Android smartphones, which might have limited the inclusion of participants who used other types of smartphones Only Android users or those willing to switch to an Android device for the study could participate, potentially introducing a selection bias. Technical Issues:  There were technical challenges related to the functionality of the BDmon app, particularly in early 2018, which affected the continuity and completeness of data collection Additionally, issues like monitoring daily traveled distance using satellite navigation were problematic, leading to high refusal rates and missing data. Nonrandom Missing Data:  Data collection issues arose particularly with patients in manic states who were more likely to turn off their smartphones or uninstall the app, leading to nonrandom missing data which could bias the results. Privacy Concerns:  Nearly 90% of the participants refused to allow tracking of their traveled distance due to privacy concerns, significantly limiting the data available for analysis in this area. Exploratory Nature:  The study was exploratory, and no correction for multiple comparisons was applied in the statistical analysis, which could increase the risk of Type I errors (false positives). Variability in Phone Usage:  Variability in individual phone usage patterns could affect the reliability of using these behaviors as standardized indicators of mood states across different patients. |
|  | The relationship between wearable-derived sleep features and relapse in Major Depressive Disorder | F. Matcham, E. Carr, N. Meyer, K.M. White, C. Oetzmann, D. Leightley, F. Lamers, S. Siddi, N. Cummins, P. Annas, G. de Girolamo, J.M. Haro, G. Lavelle, Q. Li, F. Lombardini, D.C. Mohr, V.A. Narayan, B.W.H.J. Penninx, M. Coromina, G. Riquelme Alacid, S.K. Simblett, R. Nica, T. Wykes, J.C. Brasen, I. Myin-Germeys, R.J.B. Dobson, A.A. Folarin, Y. Ranjan, Z. Rashid, J. Dineley, S. Vairavan, M. Hotopf, on behalf of the RADAR-CNS consortium | In relapse analysis (n=218, 624 assessments), greater variability in sleep duration (OR=1.25, 95% CI=1.02–1.53), variability in sleep midpoint (OR=1.28, 95% CI=1.03–1.58), and higher sleep fragmentation (OR=1.32, 95% CI=1.08–1.62) were associated with increased relapse risk, while higher sleep efficiency was associated with reduced relapse risk (OR=0.79, 95% CI=0.64–0.98). In severity analysis (n=393, 1361 assessments), greater variability in sleep duration (β=0.93, 95% CI=0.47–1.40), variability in sleep midpoint (β=0.69, 95% CI=0.23–1.14), and higher sleep fragmentation (β=0.72, 95% CI=0.28–1.16) were associated with higher depressive symptom severity, while higher sleep efficiency (β=−0.66, 95% CI=−1.08 to −0.24) was associated with lower severity. | Greater variability in sleep duration and midpoint, along with higher sleep fragmentation, were linked to increased risk of relapse and greater depressive symptom severity in individuals with recurrent MDD. Higher sleep efficiency appeared protective, being associated with reduced relapse risk and lower symptom severity. These results suggest that improving sleep regularity and quality may help prevent relapse and reduce symptom burden in MDD, highlighting sleep features as potential targets for intervention. | Potential underpowering of the primary outcome due to fewer relapses identified than anticipated. |
|  | Behavioral activities collected through smartphones and the association with illness activity in bipolar disorder | Faurholt-Jepsen, Maria; Vinberg, Maj; Frost, Mads; Debel, Sune; Margrethe Christensen, Ellen; Bardram, Jakob E.; Kessing, Lars Vedel | Patterns in Smartphone Usage and Symptom Severity:  With increased severity of depressive symptoms: The smartphone screen was on longer (more screen-on time per day). There were more incoming calls per day. Fewer outgoing calls were made per day. Incoming calls were less frequently answered. Patients showed reduced movement between cell tower IDs, indicating decreased physical movement. With increased severity of manic symptoms: There were more outgoing text messages per day. Phone calls were longer. Incoming text messages had fewer characters. Outgoing calls had shorter durations. Patients moved more between cell tower IDs, indicating increased physical activity.Mood correlated negatively with the Hamilton Depression Rating Scale (HDRS) scores, indicating that lower self-reported mood was associated with higher depression scores. There was a positive correlation with the Young Mania Rating Scale (YMRS) scores, showing that higher self-reported mood corresponded to higher mania scores. Notably, mood on a scale from -3 to +3 had coefficients of -0.049 for HDRS (p<0.001) and +0.045 for YMRS (p<0.001) in adjusted models. Outgoing text messages/day were positively correlated with YMRS scores, coefficient +0.31 (p=0.01). Screen "on" time in seconds/day showed a positive correlation with HDRS, coefficient +209.6 (p=0.04). Changes in cell tower IDs/day were negatively correlated with HDRS, coefficient -0.56 (p=0.004) and positively with YMRS, coefficient +0.40 (p=0.04). Sub-item analyses highlighted that specific behaviors like the number of outgoing text messages were positively correlated with mania-related activities on the YMRS.  Significant differences in outgoing text messages and screen "on" time were observed between euthymic, depressive, and manic states, illustrating how these markers varied across different mood states. | Correlation with Clinical Scales: The data collected from smartphones showed significant correlations with clinical ratings of depression and mania, measured by the Hamilton Depression Rating Scale (HDRS) and the Young Mania Rating Scale (YMRS) Objective smartphone data such as the number of calls, duration of screen time, and movement between cell towers correlated with symptom severity.  Behavioral Indicators:  Depressive Symptoms: Increased depressive symptoms were associated with longer screen-on times, more received calls, fewer outgoing calls, and less movement between cell tower locations, suggesting reduced physical and social activity. Manic Symptoms: Increased manic symptoms correlated with more outgoing text messages, longer phone calls, and increased physical movement as indicated by more frequent changes between cell tower IDs. Discriminative Ability: The smartphone data were able to distinguish between affective states (e.g., euthymic, depressive, manic states), indicating that these behavioral markers can be effective in identifying different mood states in bipolar disorder. | Sample Size and Composition:  The study had a relatively small sample size, which might limit the generalizability of the findings Small sample sizes can also reduce the statistical power of the analysis, potentially affecting the reliability of the conclusions. Short Duration:  The duration of the study was limited to 12 weeks This shorter follow-up period might not fully capture the longitudinal dynamics of bipolar disorder, especially given its episodic nature with potential for long-term fluctuations in mood states. Technical and Operational Limitations:  The study relied on a specific smartphone application and did not include popular operating systems like iOS This limitation could exclude a significant portion of the population and introduce bias, as the type of smartphone and operating system used can be associated with demographic and socioeconomic variables. Dependence on Participant Compliance:  The effectiveness of data collection was heavily dependent on participants carrying the smartphone with them at all times and using it as their primary communication device. |
|  | Daily electronic self-monitoring in bipolar disorder using smartphones – the MONARCA I trial: a randomized, placebo-controlled, single-blind, parallel group trial | Faurholt-Jepsen, M.; Frost, M.; Ritz, C.; Christensen, E. M.; Jacoby, A. S.; Mikkelsen, R. L.; Knorr, U.; Bardram, J. E.; Vinberg, M.; Kessing, L. V | Depressive Symptoms: The intention-to-treat analyses using linear mixed models showed no significant effects of daily self-monitoring using smartphones on depressive symptoms overall However, there was a tendency towards more sustained depressive symptoms in the intervention group compared to the control group, quantified as B = 2.02, with a 95% confidence interval of −0.13 to 4.17, and a p-value of 0.066.  Manic Symptoms: There were no significant effects on manic symptoms overall as per the intention-to-treat analyses Sub-group analysis among patients without mixed symptoms and patients with the presence of depressive and manic symptoms showed significantly more depressive symptoms and fewer manic symptoms during the trial period in the intervention group. | Electronic Self-Monitoring: Although intuitive and appealing, electronic self-monitoring needs critical consideration and further clarification before it is implemented as a clinical tool The study found that while electronic self-monitoring did not significantly affect depressive and manic symptoms overall, the approach did not conclusively improve patient outcomes and suggested a need for more targeted approaches or refinements in the tool used.  Depressive Symptoms: There was a trend where the intervention group, which used daily electronic self-monitoring, tended to have more sustained depressive symptoms compared to the control group This suggests that while the monitoring tool was effective in gathering data, it might have influenced patients' focus on their symptoms, possibly exacerbating them or affecting their perception.  Manic Symptoms: In contrast to depressive symptoms, the subgroup analysis indicated that the intervention group had fewer manic symptoms compared to the control group among those who exhibited these symptoms This suggests that the monitoring tool might be more effective in managing or tracking manic phases.  Patient Adherence: The study highlighted high patient acceptance and adherence to the use of smartphones for monitoring, indicating the feasibility of using such technology in managing bipolar disorder. | Non-Blinding of Participants and Clinicians: Due to the nature of the intervention involving smartphones, it was not possible to blind the patients or the clinicians to the allocation of the intervention or control group This could introduce bias as participants and clinicians were aware of the treatment being administered.  Specific Patient Population: The trial was conducted at a specialized mood disorder clinic, which may limit the generalizability of the findings The patients had access to highly specialized care, which might not be available in all clinical settings.  Potential Ceiling Effect: Patients were included in a remitted or partially remitted phase with relatively few depressive and manic symptoms at baseline, which might have limited the apparent effect of the intervention This might make it difficult to observe significant changes due to the intervention.  Complexity of the Intervention: The intervention combined several components (smartphone monitoring and feedback loops), making it challenging to distinguish which aspects were most effective or if certain components contributed more to the observed effects.  Control Group Intervention: The control group received a smartphone for normal use without the monitoring system, which could have had its own psychological impact, potentially confounding the results.  High Adherence as a Limiting Factor: High adherence to the intervention might reflect its usability and low intrusiveness rather than its effectiveness in symptom management.  Short Duration and Follow-up: The trial period might have been too short to observe significant changes in coping strategies or other long-term outcomes that could be influenced by the intervention. |
|  | Differences in mobility patterns according to machine learning models in patients with bipolar disorder and patients with unipolar disorder | Faurholt-Jepsen, Maria; Busk, Jonas; Rohani, Darius Adam; Frost, Mads; Tønning, Morten Lindberg; Bardram, Jakob Eyvind; Kessing, Lars Vede | A total of 65 patients with bipolar disorder (BD subtype I: 37) and 75 patients with unipolar disorder (UD) provided varying levels of patient-reported smartphone-based data and/or passively collected smartphone-based location data during the trials The patients with BD provided smartphone-based data on mood for an average of 91.1 days and location data for an average of 39.9 days In contrast, patients with UD provided mood data for an average of 97.8 days and location data for 27.8 days Overall, there were 5924 observations of smartphone-based mood data for patients with BD and 7337 for patients with UD... | Mobility Patterns During Depressive States: Patients with BD exhibited statistically significantly lower mobility than those with UD during depressive states This included a smaller area of movement per day, a lower total distance of moves, and a significantly shorter duration of moves Specifically, patients with BD had a 26% lower duration of moves per day compared to patients with UD.  Location Entropy Differences: Across all affective states, including euthymic states, patients with BD showed lower location entropy compared to UD patients This suggests that patients with BD had less variability in their location, indicating more routine and less chaotic movements compared to those with UD.  Classification Accuracy: The classification models using combined passively collected smartphone-based location data demonstrated good diagnostic accuracy... | ample Size and Statistical Power: The sample size was relatively small, which could limit the statistical power of the findings This issue is especially critical given the variability in depressive symptom severity among the participants.  Generalizability: The findings might not be generalizable to all patients with bipolar disorder and unipolar disorder, as the study participants were capable of using smartphones and participated in the data collection process actively This could exclude more severely affected individuals who might be less likely to engage with technology. |
|  | Smartphone data as objective measures of bipolar disorder symptoms | Faurholt-Jepsen, Maria; Frost, Mads; Vinberg, Maj; Christensen, Ellen Margrethe; Bardram, Jakob E.; Kessing, Lars Vedel | Throughout the 3-month follow-up, no patients dropped out The participants' mean age was 33.4 years (SD: 9.5), with 82.4% (14 patients) diagnosed with bipolar disorder type I Employment status among the patients was varied: 35.3% (6 patients) were employed (five in full-time roles and one part-time), 17.6% (3 patients) were unemployed, 11.8% (2 patients) were on sick leave, 29.4% (5 patients) were students, and 5.9% (1 patient) was on disability pension The adherence rate for self-assessments in the MONARCA system was 88%, and the completion rate for clinical ratings was 100%... | Correlation between Self-rated Mood and HDRS-17 Scores:  There was a significant correlation between self-reported mood scores and clinically rated depressive symptoms on the HDRS-17 Specifically, higher depressive scores on HDRS-17 were associated with lower self-rated mood scores, suggesting that self-monitoring via smartphone can reflect clinically significant changes in mood states. Lack of Correlation with YMRS Scores:  The study did not find a significant correlation between self-rated mood scores and the Young Mania Rating Scale (YMRS) scores, indicating that self-reported mood may not be as effective in detecting or correlating with manic states compared to depressive states. Mobility Patterns Indicated by Cell Tower Changes:  A significant finding from the unadjusted model showed that fewer changes in cell tower IDs per day were associated with higher depressive scores on HDRS-17 This suggests that increased depressive symptoms are linked with decreased physical mobility, as patients with higher depressive scores tended to change locations less frequently. Adjustments for Age and Sex:  When the model was adjusted for age and sex, the correlation between cell tower changes and HDRS-17 scores became borderline significant, still indicating a trend where more severe depressive symptoms could lead to less movement and fewer location changes. Non-Significant Trends in Communication Patterns:  The study observed non-significant trends, such as a decrease in the number of outgoing calls with higher depressive scores, suggesting potential changes in social behavior associated with more severe depressive symptoms. | Small Sample Size:  The study involved only 17 participants, which limits the statistical power and generalizability of the findings Small sample sizes may not accurately represent broader populations and can result in less reliable conclusions. Lack of Diversity in Technological Preference:  Three potential participants declined to participate due to a preference for iPhones, indicating a selection bias towards users comfortable with Android devices This could limit the applicability of the findings to a broader demographic that uses different types of smartphones. Non-representation of Manic Symptoms:  The study did not find significant correlations with manic symptoms as measured by YMRS, possibly due to the low prevalence of manic symptoms among participants during the study period This limits the study's utility in assessing manic phases of bipolar disorder. Subjective Nature of Self-Reported Data:  Self-reported mood ratings can be subjective and may not always accurately reflect true mood states... |
|  | Smartphone sensing of social interactions in people with and without schizophrenia | Fulford, Daniel; Mote, Jasmine; Gonzalez, Rachel; Abplanalp, Samuel; Zhang, Yuting; Luckenbaugh, Jarrod; Onnela, Jukka-Pekka; Busso, Carlos; Gard, David E. | individuals with schizophrenia (SZ) reported significantly higher levels of dispositional loneliness and lower levels of interviewer-rated social functioning compared to controls, both with high statistical significance (p < 0.01) Although there was no significant difference in the quantity of GPS data points or audio data collected between the groups, a small effect size indicated that controls had slightly more usable GPS data points than the SZ group (Cohen’s d = 0.38).  Females with SZ showed lower overall social functioning compared to males (t = 2.38, p < 0.05) Employed individuals with SZ reported more social interactions per ecological momentary assessment (EMA) prompt (mean = 2.23) compared to unemployed individuals (mean = 1.52; t = -2.30, p < 0.05) Those with SZ who were married or cohabitating had higher social functioning than those who were unmarried or not cohabitating (t = -2.47, p < 0.05).  No demographic variables were related to EMA-reported time spent alone or dispositional loneliness... | Mobility Metrics and Social Interactions:  In the control group, there was a significant association between the average number of EMA-reported interactions and longer daily distances traveled Additionally, more time spent alone was linked to spending more time at home and adhering to a weekday routine. For those with schizophrenia, these mobility metrics were mostly unrelated to EMA-reported social interactions, suggesting different patterns of mobility in relation to social activity compared to controls. Voice Activity Detection (VAD):  In controls, the number of EMA-reported interactions was moderately associated with longer durations of detected voice activity, suggesting a correspondence between social interaction and voice activity. In contrast, for individuals with schizophrenia, voice activity was not related to the number of EMA-reported interactions but was significantly higher when participants reported being alone, suggesting potential differences in how social interaction is perceived or reported by individuals with schizophrenia. Social Functioning and Loneliness:  Controls showed clear associations between higher dispositional loneliness and several mobility metrics like shorter movement durations and more time spent at home, which indicates a pattern where loneliness influences how individuals engage in social environments. In people with schizophrenia, higher dispositional loneliness was associated with fewer significant locations visited, but interestingly, more distance traveled and longer movement durations were noted This contrast with controls may reflect a more complex relationship between loneliness, social functioning, and mobility in schizophrenia. Differences in Correlation Magnitudes:  Statistically significant differences between the groups were observed in terms of the association between loneliness and mobility metrics For example, loneliness was associated with movement duration differently in controls compared to individuals with schizophrenia. Implications for Social Impairment:  The study provided evidence that smartphone-based digital phenotyping, through both active (EMA) and passive (sensor-based) measures, could offer valuable insights into social behavior and functioning among those with schizophrenia compared to controls... | Sample Size:  The study acknowledged that the sample size was relatively small This limitation could potentially affect the power to detect subtle differences and complex interactions between the variables studied. Generalization of Findings:  Because all participants used smartphones provided by the study, there may be concerns regarding how well the findings generalize to everyday use of personal devices by individuals with schizophrenia The extent to which participants kept the phones 'on hand' might have influenced the results, especially if there were differences in personal phone usage habits between controls and individuals with schizophrenia. Quality of Social Relationships:  The study did not focus on the quality of social relationships, which can significantly impact mental health outcomes Social interactions are not always supportive and can sometimes be sources of stress and conflict, potentially influencing the results in ways not captured by the study. Complexity of Social Behavior:  The study primarily captured quantitative aspects of social interactions and mobility but did not deeply analyze the qualitative aspects of social behavior, such as the emotional or psychological significance of social contacts or the nature of social interactions. Potential Confounds:  The study could not adequately test for potential confounding factors such as age and race/ethnicity on the associations examined, which might influence both the generalization and interpretation of the findings. Use of Loaner Phones:  Since the study involved providing participants with study phones, this might have affected how participants engaged with the devices compared to their personal phones... |
|  | Digital Communication Biomarkers of Mood and Diagnosis in Borderline Personality Disorder, Bipolar Disorder, and Healthy Control Populations | Gillett, George; McGowan, Niall M.; Palmius, Niclas; Bilderbeck, Amy C.; Goodwin, Guy M.; Saunders, Kate E. A | Mood Symptoms: Across all participants, manic symptoms were associated with higher frequencies and durations of phone calls (total and outgoing), but not with average durations Specifically, manic symptoms correlated with a 0.27 increase in total call frequency and a 0.22 increase in outgoing call frequency In terms of duration, a single unit increase in manic symptoms increased total call duration by 70.91 seconds, incoming call duration by 33.50 seconds, and outgoing call duration by 37.70 seconds For depressive symptoms, they were positively linked to average call durations—increasing mean total call duration by 3.336 seconds and mean incoming call duration by 5.104 seconds... | Manic Symptoms and Communication:  Manic symptoms were positively associated with both the frequency and duration of phone calls and text messages Increased manic symptoms led to more frequent and longer phone calls and texts, suggesting heightened communication activity during manic states. Depressive Symptoms and Communication:  Depressive symptoms were associated with longer durations of incoming phone calls but not with the frequency of calls or texts This might reflect more prolonged conversations possibly due to slower speech or longer pauses which are common during depressive episodes. Bipolar Disorder (BD) and Communication:  Participants with Bipolar Disorder exhibited increased total call frequencies compared to healthy controls, suggesting more frequent phone use However, when in a depressive state, these participants showed a decrease in both the frequency and duration of phone calls. Borderline Personality Disorder (BPD) and Communication:  Individuals with Borderline Personality Disorder had increased frequencies and lengths of text messages compared to healthy controls, indicating a preference for text communication over voice calls in this group. Differential Effects by Mood State:  Manic states were associated with increases in both the frequency and total duration of phone calls and texts... | Sample Size and Composition:  The sample size was relatively small (55 participants), which may limit the generalizability of the findings Additionally, the gender distribution was uneven across the diagnostic groups, particularly with a low number of male participants in the Borderline Personality Disorder group. Use of Provided Smartphones:  Participants were provided with smartphones to ensure consistent data collection This could introduce bias if participants continued to use their personal devices alongside the study-provided smartphones, potentially skewing the data collection. Scope of Digital Communication Tracking:  The study focused on traditional forms of digital communication (calls and SMS) and did not include newer forms such as social media and instant messaging platforms, which are increasingly prevalent and could provide additional insights into digital communication behaviors. Self-reported Mood Assessments:  Mood states were assessed using self-report measures, which can be subjective and potentially less reliable than clinical evaluations, especially if the mood states themselves affect the participants’ ability to report accurately. High Frequency of Assessments:  The frequent assessments required for the study could lead to participant fatigue, potentially affecting their engagement and the accuracy of the data collected over time. Potential for Unobserved Influences:  Factors such as personality traits and social behaviors that could influence communication patterns were not controlled for in the study These factors could confound the relationships observed between communication behaviors and mood or diagnostic categories. Analytical Complexity:  The study involved multiple analyses and did not adjust for multiple testing, which could increase the risk of type I errors (false positives)... |
|  | Smartphone-Based Recognition of States and State Changes in Bipolar Disorder Patients | Grünerbl, Agnes; Muaremi, Amir; Osmani, Venet; Bahle, Gernot; Ohler, Stefan; Tröster, Gerhard; Mayora, Oscar; Haring, Christian; Lukowicz, Paul | State Recognition Accuracy: The study achieved a recognition accuracy of 76% by fusing all sensor modalities. Phone (Communication behavior via phone calls): Average Accuracy: 66% Average Recall: 61% Average Precision: 58% Sound (Audio features during phone calls): Average Accuracy: 70% Average Recall: 60% Average Precision: 59% Fusion (Combination of phone and sound data): Average Accuracy: 69% Average Recall: 52% Average Precision: 55% Change Detection Precision and Recall: The precision and recall for detecting changes in mental states were over 97%​​ accelerometer and location data, both individually and combined, for state recognition:  Phone (Data based on phone usage): Recall: 54.4% Precision: 37.3% Sound (Audio features): Recall: 61.3% Precision: 24.2% Fusion P + S (Combination of phone and sound data): Recall: 58.4% Precision: 59.0% ACC (Accelerometer data): Recall: 62.9% Precision: 64.8% LOC (Location data): Recall: 72.3% Precision: 76.5% Fusion A + L (Combination of accelerometer and location data): Recall: 66.3% Precision: 57.9% All-in Fusion (Combination of all modalities): Recall: 65.8% Precision: 72.0% effectiveness of different fusion strategies in detecting state changes:  A+L Weighted Fusion (Accelerometer and location data with weighted fusion): Recall: 96.40% Precision: 94.50% All-in AND Fusion (Requires all sensors to agree on a state change): Recall: 42.87% Precision: 61.18% All-in OR Fusion (Any sensor can indicate a state change): Recall: 92.15% Precision: 70.28% All-in WEIGHTED Fusion (Weighted combination of all sensor modalities): Recall: 97.36% Precision: 97.19% | The study demonstrated the effectiveness of using smartphone-sensing data to recognize depressive and manic states in bipolar disorder patients, as well as to detect state changes The use of multiple sensor modalities (e.g., GPS, acceleration, phone usage, and sound data) significantly improved the system's ability to accurately detect and differentiate between states The high precision and recall in change detection indicate that the system could potentially provide reliable and early warnings of state changes, which is crucial for timely intervention in bipolar disorder management. | Small Sample Size: The study was conducted with only ten patients This small sample size limits the generalizability of the findings to a broader population of bipolar disorder patients.  Variability in Data Collection: The data was collected from patients in their real-life settings, leading to variability in how often and how consistently the smartphone sensors were used This included patients sometimes turning off sensors to save battery or forgetting to charge the phone, which resulted in incomplete data capture on some days.  Irregular Ground Truth Data: The clinical assessments used as ground truth for validating the sensor data were only conducted every three weeks, which may not have captured all relevant changes in patient states Additionally, reliance on patient self-assessment could introduce subjectivity and affect the accuracy of these ground truth data.  Complexity in Data Processing and Analysis: The study involved complex processing and analysis of multimodal sensor data... |
|  | Testing Suicide Risk Prediction Algorithms Using Phone Measurements With Patients in Acute Mental Health Settings: Feasibility Study | Haines-Delmont, Alina; Chahal, Gurdit; Bruen, Ashley Jane; Wall, Abbie; Khan, Christina Tara; Sadashiv, Ramesh; Fearnley, David | Algorithm Performance: The K-nearest neighbors (KNN) algorithm with k=2 using uniform weighting and the Euclidean distance metric was identified as the most promising This algorithm achieved a mean accuracy of 68%, averaged over 10,000 simulations involving 10-fold cross-validation The average area under the curve (AUC) was 0.65.  Comparative Analysis: A combined 5×2 F test was applied to compare the KNN model's performance against other models, such as the baseline classifier that guesses training majority, random forest, support vector machine, and logistic regression The F statistics were significant at 10.7 and 17.6 for training majority and random forest, respectively, indicating a rejection of the null hypothesis that the performance was the same. | The study's findings indicate that using smartphone-generated user input and passive sensor data is feasible for developing a risk algorithm among inpatients at suicide risk The model showed a fair concordance between phone-derived and research-generated clinical data The use of machine learning, specifically the K-nearest neighbors algorithm, demonstrated the potential for accurate suicide risk prediction using mobile devices These findings suggest that mobile technology can be integrated into mental health settings to enhance real-time monitoring and management of suicide risk. | Data Collection and Model Generalizability: The data was collected from a relatively small cohort (66 participants), which may limit the generalizability of the findings The small sample size could also affect the robustness and reliability of the predictive model.  Short Follow-up Period: The study's follow-up period was limited to up to 7 days, which might not be sufficient to capture longer-term patterns or changes in suicide risk.  Predictive Model Complexity: The complexity of the predictive models used may result in overfitting, where models perform well on training data but less so on unseen data. |
|  | Deriving symptom networks from digital phenotyping data in serious mental illness | Hays, Ryan; Keshavan, Matcheri; Wisniewski, Hannah; Torous, John | High transition probabilities were found for several symptoms, specifically: Anxiety-inducing mood had a transition probability of 0.357 with a significance level of P < 0.001. Psychosis-inducing mood had a transition probability of 0.276 with a significance level of P < 0.001. Anxiety-inducing poor sleep had a transition probability of 0.268 with a significance level of P < 0.001​​. | Symptom Networks: The study demonstrated that certain symptoms of schizophrenia might trigger or elevate other symptoms in future time steps This supports a network approach to understanding the dynamic and interconnected nature of psychopathology in schizophrenia​​.  High Transition Probabilities: Specific high transition probabilities were identified, including:  Anxiety inducing mood changes, Psychosis inducing mood changes, and Anxiety inducing poor sleep​​. Generative Models: Using generative models, the study illustrated how digital phenotyping can help map the temporal dynamics of symptom interactions This approach allows for the identification of how certain symptoms can influence the onset or worsening of other symptoms over time​​.  Clinical Implications: The results suggest that understanding these symptom interactions can aid in creating more targeted interventions for schizophrenia, potentially allowing for preemptive actions to mitigate worsening symptoms or the onset of new symptoms​​. | The study's limitations included the relatively small sample size, especially for the validation set, which may have influenced the reliability of the findings Also, the study only considered transitions across 3-day intervals, which may not capture more immediate symptom interactions​​. |
|  | Anomaly detection to predict relapse risk in schizophrenia | Henson, Philip; D'Mello, Ryan; Vaidyam, Aditya; Keshavan, Matcheri; Torous John | Single Anomalies: Overall, 73 participants experienced at least one anomaly, contributing to a total of 1006 anomalies The monthly anomaly rates per participant ranged from as low as 0 to as high as 4.7, specifically noted in the mobility category Variations were also observed between anomaly rates across different individual features.  Paired Anomalies: Out of all participants, 39 (28 individuals with schizophrenia and 11 healthy controls) experienced at least one day with paired anomalies Among those with schizophrenia, 7 (25%) had anomalies that combined survey data with passive data anomalies, while the remaining had anomalies involving only passive data... | Effectiveness of Anomaly Detection:  Anomaly detection demonstrated high sensitivity (89%) and reasonable specificity (75%) in predicting clinical relapses This suggests that the approach is quite effective at identifying patients who are at risk of relapse, though there's some room to improve in reducing false positives. Rates of Anomalies:  The study observed considerable variation in anomaly rates across participants, ranging from zero to as high as 4.7 anomalies per month in the mobility data category This variation indicates the complex and individual nature of behavioral patterns in schizophrenia. Paired Anomalies:  A significant number of participants experienced at least one day with paired anomalies, which involved combinations of data anomalies from both survey and passive data sources These paired anomalies were found in both schizophrenia patients and healthy controls, albeit more frequently in the former. Impact of COVID-19:  The study noted that no paired anomalies were detected after the initiation of social distancing measures due to COVID-19... | Variability in Study Duration and Data Collection:  The duration of participation varied among subjects, and so did the frequency of ecological momentary assessments (EMAs) This variability could affect the rates of detected anomalies, as longer participation or more frequent assessments provide more data, potentially leading to better anomaly detection. Smartphones as Proxies for Behavior:  Smartphones are used as proxies for participant behavior, which can lead to inaccuracies For instance, a phone left on a table, indicating inactivity, might not accurately represent the user's actual physical activity or mental state. Definition and Timing of Relapse Events:  Some definitions of relapse used in the study do not provide specific dates, making it challenging to precisely identify when interventions should be applied The lack of specific timing for certain relapse indicators (like changes in PANSS or CGI scores) complicates the use of anomaly detection for timely interventions. Control Group Matching:  The control group was not matched perfectly with the patient group in terms of age and race, which could introduce confounding variables affecting the study's outcomes. High Levels of Psychopathology in Controls:  The study noted that a significant number of healthy controls also exhibited high levels of detected anomalies, potentially indicating unaccounted psychopathology or other stressors affecting these individuals, which could skew the results and affect the positive predictive value of the anomaly detection. No Relapses During COVID-19 Period:  The lack of relapse events or paired anomalies during the COVID-19 pandemic period might not accurately reflect the general effectiveness of the anomaly detection method under normal circumstances... |
|  | Towards clinically actionable digital phenotyping targets in schizophrenia | Henson, Philip; Barnett, Ian; Keshavan, Matcheri; Torous, John | Clustering Analysis:  In the clustering analysis comparing active data mean and variance (e.g., psychosis score) with passive data mean and variance (e.g., Hometime), 19% of participants (4 out of 21) showed overlap between specific active and passive data features There was no overlap among participants in the cluster with the greatest mean/variance. Correlation Matrix:  A correlation matrix was created to analyze the interactions between active and passive data features Significant correlations were found, particularly within the schizophrenia group, more so than in healthy controls Significant correlations that were shared between the groups often displayed reversed polarity... | Clinical Utility of Passive Data:  Greater dysregulation in social rhythms, suggesting less routine, correlated with higher severity of self-reported depression, anxiety, psychosis, and sleep symptoms in individuals with schizophrenia but not in controls This indicates that passive data can be actionable for setting therapeutic goals and monitoring both recovery and relapse. Active and Passive Data Correlation:  The study found that correlations between active and passive data were heterogeneous among individuals with schizophrenia, with only 19% agreement, suggesting that passive data are not always a direct proxy for mental health symptoms. Potential Clinical Applications:  The results support potential clinical applications such as population-level screening for psychosis risk based on early detection of passive data irregularities like circadian routine disturbances, as well as individual-level monitoring to improve social rhythms and reduce symptom severity. Insight into Digital Phenotypes:  The study suggests that certain digital phenotypes may emerge on the population level through clustering analysis, which could help stratify current definitions of schizophrenia and potentially guide personalized treatment approaches. | Data Quality and Missingness:  The study noted that data quality and missingness could significantly affect the results, potentially due to poor engagement, technical issues with smartphones or apps, or third-party apps leading to uncaptured sociability features. Smartphone App Sensitivity:  The smartphone app used was originally designed for individuals with schizophrenia, who might have more severe and variable symptoms than healthy controls, potentially making the app more sensitive to picking up behavior and symptomatology in schizophrenia than in healthy controls. Sample Matching:  Schizophrenia participants and healthy controls were not matched for age, race, and education, which could introduce confounding factors, particularly affecting the effect of age on circadian rhythm. |
|  | Investigating Associations Between Screen Time and Symptomatology in Individuals With Serious Mental Illness: Longitudinal Observational Study | Henson Philip; Rodriguez-Villa Elena; Torous John | At baseline, correlations between screen time metrics and various cognitive subdomains (such as Verbal Memory and Symbol Coding) ranged from -0.17 to 0.29, with no significant correlations identified (P values from .25 to .98) Longitudinally, a multivariate regression analysis showed a significant relationship between screen time metrics and cognitive performance in participants with schizophrenia (F=5.43, P<.001, adjusted R² = 0.107) This analysis considered factors like the number of screen time sessions and checks In contrast, the same analysis did not yield significant results for the healthy control group (adjusted R² = 0.035, P = .068).  Specification Curve Analysis A comprehensive specification curve analysis covering all 88 participants displayed a broad range of associations between screen time and various symptoms, with regression coefficients ranging widely from -1.19 to 1.05... | Variability in Associations: The study highlighted a considerable heterogeneity in the associations between screen time and various mental health symptoms These associations varied widely, ranging from very negative to very positive, depending on the diagnostic group, age bracket, and type of regression model used.  Cognition and Screen Time: Longitudinal analysis showed a significant, though modest, association between screen time metrics and cognitive performance in individuals with schizophrenia, as measured by the Jewels B cognitive task This suggests that screen time has some impact on cognitive functions, though the degree of influence is relatively small (adjusted R² = 0.107).  Specification Curve Analysis (SCA): This analysis revealed a broad spectrum of possible outcomes based on different analytical approaches, suggesting that the effect of screen time on mental health symptoms is complex and influenced by multiple factors The SCA showed that different combinations of variables could lead to varied interpretations of how screen time affects mental health.  Individual Variability: The effects of screen time on mental health symptoms were not uniform across individuals... | Variability in Data Collection: The study noted that behavioral and symptomatology data could vary throughout the day, but the method used aggregated the data daily This approach may not capture intraday fluctuations that could provide more granular insights into the effects of screen time.  Survey Completion: Not all participants completed the surveys comprehensively The analysis included only those who completed surveys across all four categories, potentially excluding valuable data from less engaged participants This might affect the generalizability of the findings.  Potential Time Lag Issues: The study paired screen time and symptom data from the same day, which may not accurately reflect the lagged effects where changes in symptoms could follow changes in screen time usage after a delay... |
|  | Digital Biomarkers of Social Anxiety Severity: Digital Phenotyping Using Passive Smartphone Sensors | Jacobson, Nicholas C.; Summers, Berta; Wilhelm, Sabine | A correlation coefficient (r) of 0.702 was found between predicted and observed social anxiety symptom severity, suggesting a strong predictive ability of the smartphone sensor data. The results also showed discriminant validity between depression, negative affect, and positive affect The correlations between the predicted SAD severity from smartphone biomarkers and these measures were significantly lower than the correlation with observed SAD severity: Correlation with depression severity: r = 0.357 Correlation with negative affect: r = 0.384 Correlation with positive affect was negative and not significant: r = -0.138 | The study successfully demonstrated that smartphone sensor data could predict social anxiety disorder (SAD) symptom severity with a moderately strong accuracy (correlation coefficient r=0.702). This predictive ability was strong enough to distinguish between social anxiety symptoms and other psychological states such as depression, negative affect, and positive affect, showcasing discriminant validity. The results suggest that simple, passively collected behavioral data can be a feasible and specific tool for identifying individuals at risk of significant social anxiety, pointing towards the potential of mobile technology in mental health assessment and intervention. | Modest Sample Size and Demographic Limitations: The sample was modest in size and consisted entirely of undergraduate students, which may limit the generalizability of the findings As the study was conducted on a relatively homogeneous group, the findings may not apply to other demographic groups, such as older adults or individuals from different educational backgrounds.  Nature of Passive Data Collection: The passive data collection method used does not allow for a qualitative assessment of the social interactions, such as whether the contacts were with close friends or mere acquaintances This limits the depth of insight that can be drawn from the data, as the nature of social contacts can significantly influence social anxiety dynamics.  Predictive Modeling Focus on Between-Person Differences: The predictive models were trained to distinguish between-person differences in social anxiety symptoms, rather than within-person variations over time This approach might not effectively capture the fluctuating nature of social anxiety symptoms as experienced by individuals over different times or situations. |
|  | MoodSensing: A smartphone app for digital phenotyping and assessment of bipolar disorder | Jia-Hao Hsu, Chung-Hsien Wu, Esther Ching-Lan Lin, Po-See Chen | Participants: 181 BD patients recruited from NCKUH.  Thresholds for inclusion:   - GPS: ≥2000 points/week - Emotional scores: ≥3 days/week - Multimedia: ≥2 days/week - Daily mood & sleep: ≥3 days/week - Self-scale: ≥1/week   3532 out of 6989 weekly data entries met the threshold criteria and were retained for further modeling.  Data Quality & Prediction Accuracy: Model: GRU deep learning model trained on sequential yearly data. Performance:   - HAM-D MAE improved from 1.25 (2020 only) to 0.81 (2020–2022) - YMRS MAE improved from 0.41 to 0.19   Emotional Feature Extraction: Models used:   - Text: RoBERTa (Micro-F1 = 59.17) - Speech: Wav2vec 2.0 (Accuracy = 93.80%) - Facial: ResNet18 (Accuracy = 89.07%)   Feature Importance in Scale Prediction   - Experiment: Masking each data type to assess impact. - Findings:   - Removing self-scale data caused largest drop in accuracy (HAM-D MAE = 2.07)   - Multimedia also critical (HAM-D MAE = 1.13)   - GPS least impactful (HAM-D MAE = 0.86)   Model Comparison   - Best performer: GRU model - Traditional models (e.g., linear regression) performed poorly. | Self-scale features (e.g., PHQ-9, GAD-7) were consistently the strongest predictors of mood scores.  Multimedia features added value but were less predictive than self-scales.  GPS-derived features had minimal impact on model performance.  The GRU model’s ability to capture temporal patterns across 7-day sequences improved prediction accuracy over static models.  The study demonstrated that passive sensing and multimodal data can support mood prediction, but self-report remains central.  The study supports the feasibility of integrating passive sensing and active self-report in smartphone-based mental health tools. | The study lacked external validation, limiting generalizability to broader clinical populations.  Staff-Dependent Interviews: Weekly scale interviews require psychiatric professionals, making data collection labor-intensive and limiting dataset size for training deep learning models.  Restricted Patient Pool: Participation requires consent and protocol adherence, resulting in a sample skewed toward stable patients and few high-scale-score cases.  Incomplete Data Modalities: Multimedia and GPS data were poorly collected, likely due to user reluctance and device limitations.  App Functionality Gaps: The app lacks a feature for patients to contact physicians directly, though clinicians can monitor usage data.  No Severity Stratification: Patients were included based on broad criteria (DSM-5 BD diagnosis, age, language, smartphone use), without classification by illness severity. |
|  | Smartwatch digital phenotypes predict positive and negative symptom variation in a longitudinal monitoring study of patients with psychotic disorders | Kalisperakis, Emmanouil; Karantinos, Thomas; Lazaridi, Marina; Garyfalli, Vasiliki; Filntisis, Panagiotis P.; Zlatintsi, Athanasia; Efthymiou, Niki; Mantas, Asimakis; Mantonakis, Leonidas; Mougiakos, Theodoros; Maglogiannis, Ilias; Tsanakas, Panayotis; Maragos, Petros; Smyrnis, Nikolaos | Heart Rate Average (HRA):  An increase in the average heart rate (HRA) during wakefulness and sleep was correlated with increases in positive psychopathology symptoms. Specifically, for wakefulness, the mean increase in HRA was positively associated with an increase in positive psychopathology symptoms with a significant p-value (p = 0.001), indicating that higher heart rates were correlated with more severe positive symptoms. Heart Rate Variability (HRV):  Decreased heart rate variability (HRV) and an increase in its monthly variance were associated with increases in negative psychopathology. For example, an increase in the standard deviation of HRV during wakefulness was correlated with an increase in negative psychopathology symptoms with a significant p-value (p = 0.005), suggesting that greater variability in heart rate was linked to more severe negative symptoms. Sleep/Wake Ratio (SWR):  Changes in the sleep/wake ratio were also found to be significant An increase in the mean sleep/wake ratio was correlated with more severe negative psychopathology symptoms with a significant p-value (p = 0.010), indicating that disturbances in sleep patterns were associated with negative symptomatology. | Heart Rate Average (HRA): The study found that increased HRA during wakefulness and sleep was associated with increases in positive psychopathology, suggesting that heart rate could be a significant indicator of psychopathology exacerbation. Heart Rate Variability (HRV): Decreased HRV and increased monthly variance in HRV were associated with increases in negative psychopathology This highlights HRV as a critical measure in understanding and predicting mental health status. Sleep/Wake Ratio (SWR): Variations in SWR were significantly correlated with changes in negative psychopathology, suggesting that alterations in sleep patterns are relevant indicators of negative symptomatology in psychotic disorders. | Small Sample Size: The study was conducted with a relatively small group of participants (35 patients), which may limit the generalizability of the findings to a broader population. Diagnostic Group Specificity: The sample included specific diagnostic groups within the spectrum of psychotic disorders, which might affect the applicability of the findings to other psychiatric conditions or to a more diverse psychiatric population. Data Aggregation: The aggregation of individual digital phenotype data over a monthly period could potentially lead to the loss of finer temporal resolution that might be crucial for understanding more immediate or acute changes in psychopathology. |
|  | Smartphone digital phenotyping, surveys, and cognitive assessments for global mental health: Initial data and clinical correlations from an international first episode psychosis study | Lakhtakia, Tanvi; Bondre, Ameya; Chand, Prabhat Kumar; Chaturvedi, Nirmal; Choudhary, Soumya; Currey, Danielle; Dutt, Siddharth; Khan, Azaz; Kumar, Mohit; Gupta, Snehil; Nagendra, Srilakshmi; Reddy, Preethi V.; Rozatkar, Abhijit; Scheuer, Luke; Sen, Yogendra; Shrivastava, Ritu; Singh, Rahul; Thirthalli, Jagadisha; Tugnawat, Deepak Kumar; Bhan, Anant; Naslund, John A.; Patel, Vikram; Keshavan, Matcheri; Mehta, Urvakhsh Meherwan; Torous, John | Demographics and Clinical Symptom Severity:  A total of 60 participants completed the initial visit: 17 at BIDMC, 20 at NIMHANS, and 23 at Sangath-AIIMS Bhopal There were no significant differences between the age and gender of participants at each site Participants at BIDMC differed by race and ethnicity from NIMHANS and Sangath-AIIMS Bhopal participants. Psychotic symptom severity was mild across the three sites, with Sangath-AIIMS Bhopal participants having the highest mean PANSS total score of 55 (standard deviation, SD=16), and NIMHANS participants having the lowest mean score of 37 (SD=15). Engagement:  The total number of activities completed per day was 8.8 (SD=7.0), with no significant difference in overall mean completion across sites The mean survey activities per day were 5.9 (SD=4.2) and games per day were 2.58 (SD=3.22). Passive Data Quality:  No significant differences emerged between GPS data quality among the three study sites, and all sites had mean GPS data quality greater than 50%. mindLAMP Real-Time Self-Report:  Symptoms reported on the EMA surveys were correlated with clinical assessments for anxiety and psychosis... | The study found no significant difference between app activities completed or digital phenotyping data collected across the three study sites App use also did not correlate to clinical or cognitive assessment scores Preliminary findings suggest that app-based assessment correlates with standard cognitive and clinical assessments​​. | Challenges Stemming from COVID-19: The research was hindered by the effects of the COVID-19 pandemic, impacting recruitment and potentially restricting the applicability of findings beyond this unique timeframe.  Constraints Related to Sample Composition: Participants exclusively consisted of individuals diagnosed with schizophrenia spectrum disorders who were actively receiving care and exhibited mild symptoms Consequently, the findings might not be readily applicable to patients not engaged in care or experiencing severe symptoms Additionally, the study's focus on younger demographics limits its extrapolation to older individuals with the same condition.  Heterogeneity Across Sites and Limited Sample Size: Variability in illness severity across different study sites, coupled with a relatively small sample size, complicates the ability to draw overarching conclusions regarding clinical implications Nevertheless, the results suggest potential efficacy across diverse clinical settings.  Language Proficiency Variation: While participants were proficient in the primary local language at each site, some might have had familiarity with English... |
|  | Prediction of impending mood episode recurrence using real-time digital phenotypes in major depression and bipolar disorders in South Korea: a prospective nationwide cohort study | Lee, Heon-Jeong; Cho, Chul-Hyun; Lee, Taek; Jeong, Jaegwon; Yeom, Ji Won; Kim, Sojeong; Jeon, Sehyun; Seo, Ju Yeon; Moon, Eunsoo; Baek, Ji Hyun; Park, Dong Yeon; Kim, Se Joo; Ha, Tae Hyon; Cha, Boseok; Kang, Hee-Ju; Ahn, Yong-Min; Lee, Yujin; Lee, Jung-Been; Kim, Leen | A total of 270 mood episodes recurred in 135 subjects during the follow-up period. The prediction accuracies for impending major depressive episodes, manic episodes, and hypomanic episodes for the next 3 days were respectively 90.1%, 92.6%, and 93.0%. The area under the curve (AUC) values were 0.937 for major depressive episodes, 0.957 for manic episodes, and 0.963 for hypomanic episodes. The study utilized algorithms that predicted mood episodes and compared these predictions to face-to-face clinical interviews that incorporated assessments of daily mood and energy. | The study successfully predicted the onset of mood episode recurrences exclusively using digital phenotypes, specifically noting that phenotypes indicating circadian rhythm (CR) misalignment contributed the most to the prediction of episodes recurrences. The monitoring of circadian rhythms using digital devices was suggested to be useful in preventing and treating mood disorders, as digital phenotyping provided a high accuracy in predicting impending mood episodes based on CR disruption. The results support the concept that proactive, real-time monitoring of physiological and behavioral patterns can effectively contribute to managing mood disorders, potentially enhancing treatment approaches. | Accuracy of Episode Onsets: The onset of mood episodes as determined during the study may not be accurate due to the retrospective nature of clinical evaluations, which could impact the reliability of the findings.  External Validation Needed: The prediction model was trained and tested within the same participant group, limiting its generalizability The study acknowledges the need for external validation to test the model on new groups of mood disorder patients to establish its applicability across different populations.  Data Collection Constraints: Due to iOS restrictions, light data could not be collected from iPhones, and the light exposure data recorded from Android devices might not have accurately reflected true light exposure These data were supplemented by imputation, which might affect the findings related to light exposure and its influence on mood episodes.  Self-report and Wearable Data Reliability: The study heavily relied on self-reported data and data from wearable devices, which may include biases or inaccuracies inherent in self-reporting methods and the sensitivity and specificity of the wearable technology used. |
|  | Digital phenotyping in bipolar disorder: Using longitudinal Fitbit data and personalized machine learning to predict mood symptomatology | Jessica M. Lipschitz, Sidian Lin, Soroush Saghafian, Chelsea K. Pike, Katherine E. Burdick | - BiMM forest ROC-AUC:  86.0% (depression), 85.2% ((hypo)mania)  Accuracy:  80.1% (depression), 89.1% ((hypo)mania)  Sensitivity: 71.2% (depression), 80.0% ((hypo)mania)  Specificity: 85.6% (depression), 90.1% ((hypo)mania)  Fitbit compliance: 87.8% of weeks  Imputation required for 4.3% of Fitbit days | High predictive accuracy using passive Fitbit data: The BiMM forest model achieved prediction accuracies of 80.1% for depression and 89.1% for (hypo)mania, respectively.  Personalized modeling outperformed generalized approaches: The BiMM forest algorithm incorporates mixed-effects modeling, allowing for individualized prediction based on each participant’s longitudinal data. This personalization was key to outperforming six other ML models.  Key predictive features identified:  For depression:   - Duration of awakenings - Total sleep time - Median bedtime - Resting heart rate - Deep sleep percentage   For (hypo)mania:   - Heart rate - Sleep efficiency - REM sleep percentage - Very active minutes - Median bedtime   Clinical relevance: The authors argue that a prediction accuracy of ~89% for (hypo)mania could justify clinical outreach between appointments. The model’s reliance on non-invasive, passively collected data makes it feasible for real-world implementation.  Minimal data filtering and high compliance: Unlike prior studies that excluded large portions of data, this study retained 95.3% of Fitbit days and included 83.1% of enrolled participants. | - Sample size and generalizability: Although the dataset was larger than many prior digital phenotyping studies, it remains modest for machine learning applications. The absence of external validation limits generalizability across populations and clinical settings.  - Ground truth measurement error: Mood symptomatology was defined using PHQ-8 and ASRM scores, which -while feasible for routine care- carry inherent error rates and may not reflect clinician-rated mood states.  - Diagnostic skew: The sample was predominantly BD I, limiting insights into BD II-specific symptom prediction.  - Device limitations: Fitbit-derived metrics (e.g., sleep stages, heart rate) are less precise than clinical-grade tools like polysomnography or ECG, and rely on proprietary algorithms.  - Missing data and filtering: Despite using random forest imputation to retain data, 11 participants were excluded due to dropout or insufficient data, and 4.3% of Fitbit days required imputation, which is argued to be enough for clinical utility.  - Compliance bias: Fitbit compliance averaged ~88%, which is higher than typical BD samples. This may reflect selection bias or study incentives, and could affect real-world applicability.  - Feature constraints: Heart rate variability -a potentially important predictor- was not captured due to Fitbit API limitations at the time of data collection.  No clinical intervention tested: While predictive accuracy was high, the study did not evaluate whether model outputs could improve clinical outcomes or guide treatment decisions. |
|  | Digital Phenotyping in Bipolar Disorder | Laura Orsolini , Michele Fiorani and Umberto Volpe | Initial analyses included 37 individuals with significant mood episodes Without pre-processing, the Area Under the Curve (AUC) for detecting depression was 0.64 and for mania was 0.57. The Samsung Galaxy S5 outperformed the S3, with significant audio clipping noted in the S3, especially during manic episodes. Applying a declipping algorithm improved the AUC for mania to 0.70 for combined data from both devices. Further analysis using 2-second segmentation across calls improved sensitivity, with AUCs of 0.74 for mania and 0.77 for depression. | Digital phenotypes derived from mobile device-captured speech can predict mood states effectively. The study demonstrates the utility of mobile technology in ongoing monitoring and assessment, offering a more objective method than traditional clinical assessments. By integrating technology into daily routines, passive monitoring systems like the PRIORI app can offer continuous insights into mood fluctuations without requiring active input from users. | Not Specified |
|  | Machine Learning Identifies Digital Phenotyping Measures Most Relevant to Negative Symptoms in Psychotic Disorders: Implications for Clinical Trials | Narkhede, Sayli M.; Luther, Lauren; Raugh, Ian M.; Knippenberg, Anna R.; Esfahlani, Farnaz Zamani; Sayama, Hiroki; Cohen, Alex S.; Kirkpatrick, Brian; Strauss, Gregory P. | Machine Learning Accuracy: The machine learning algorithms used in the study were able to classify the presence of a psychotic disorder diagnosis with an accuracy of 80% during cross-validation and a test accuracy of 79% when using the Recursive Feature Elimination with Cross-Validation feature selection model. Symptom Domain Classification: The models classifying the presence versus absence of clinically significant elevations in the five negative symptom domains (anhedonia, avolition, asociality, alogia, and blunted affect) varied in test accuracy from 73% to 91% This indicated a robust ability of the digital phenotyping measures to differentiate between the various negative symptom domains. Predictive Features: A few active and passive digital phenotyping features were highly predictive of all five negative symptom domains, but there were also unique predictors for each domain, which shows the complexity and specificity of the digital measures in relation to different symptoms of psychotic disorders. | Model Effectiveness: Machine learning models were effective in classifying diagnostic status and the presence of specific negative symptom domains (anhedonia, avolition, asociality, alogia, and blunted affect) with high accuracy. Relevance of Digital Measures: Specific active and passive digital phenotyping variables were found to be highly predictive of negative symptoms The study identified unique predictors for each symptom domain, indicating the potential for tailored digital assessments. | Generalizability: The models were developed and validated on a relatively small and specific sample, which may limit the generalizability of the findings to broader populations. Complexity of Negative Symptoms: Negative symptoms are complex and multifaceted, and the digital phenotyping measures may not capture all aspects of these symptoms comprehensively. Dependence on Self-Reported Measures: While the study leveraged both active (self-reported via surveys) and passive (sensor-based) data, the reliance on self-reported data can introduce biases, such as social desirability and recall bias. Technical Challenges: The study discussed the technical challenges of digital phenotyping, including the need for extensive validation of the technology and the algorithms used, which are critical for ensuring reliability and accuracy in clinical settings. |
|  | Wearable devices and mobile technologies for supporting behavioral weight loss among people with serious mental illness | Naslund John A.; Aschbrenner Kelly A.; Scherer Emily A.; McHugo Gregory J.; Marsch Lisa A.; Bartels Stephen J. | Wearable Device Use: Participants used Fitbit devices for an average of 181.7 days, with a mean usage of 86.2% of the days they were enrolled in the study The range of days with the Fitbit in use varied, but the median was 181.5 days.  Average Daily Step Count and Weight Loss and Improved Fitness: Participants averaged 4,453.5 steps per day Those who managed to achieve 10,000 steps or more on at least one day during the study constituted 61.8% of the participants, with these participants achieving 10,000 daily steps or more for a mean of 16.1% of the days they used the Fitbit A significant association was found between the average daily step count and weight loss... | The study found that a higher average daily step count over the 6-month program duration was associated with greater weight loss among participants with serious mental illness enrolled in a community-based group behavioral weight loss program This suggests that promoting higher step counts using wearable devices can be an effective strategy for weight management in this population However, while the direction of the relationship between step count and fitness was positive, it was not statistically significant, indicating that increased steps alone may not be sufficient to improve fitness levels notably without other supportive measures​​. | Small Sample Size and Lack of Diversity: The study had a small sample size and lacked racial or ethnic diversity, which may restrict the generalizability of the results to other community mental health settings or to populations with different demographic profiles​​.  Analysis Based on Engaged Participants: The analyses were based on participants who received the Fitbit devices, remained engaged in the study, and used the devices over the 6-month study duration   Generalizability to Broader Populations: The participants were all receiving services through community mental health settings, which might not make the findings representative of individuals with serious mental illness who are not receiving similar services​​. |
|  | Enhancing early psychosis treatment using smartphone technology: A longitudinal feasibility and validity study | Niendam, Tara A.; Tully, Laura M.; Iosif, Ana-Maria; Kumar, Divya; Nye, Kathleen E.; Denton, Jennifer C.; Zakskorn, Lauren N.; Fedechko, Taylor L.; Pierce, Katherine M. | Feasibility: The study had an enrollment rate of 53%, with participants remaining in the study for an average of 183 days The daily and weekly survey completion rates were high, with weekly survey completion averaging 77.3% and daily survey completion averaging 69%.  Validity of Smartphone Symptom Assessment: The smartphone assessments were found to be valid for measuring symptoms of early psychosis This was evidenced by significant associations between weekly survey symptoms and gold-standard Brief Psychiatric Rating Scale (BPRS) assessments for positive symptoms (with a correlation coefficient, β, of 0.56 for maximum weekly symptoms relating to BPRS positive symptoms) and for depression/anxiety symptoms (with a correlation coefficient, β, of 0.58) However, no significant association was found for negative symptoms, indicating that weekly self-report surveys via smartphone may not effectively capture these symptoms. | Feasibility and Validity: The study successfully demonstrated the feasibility and validity of using a smartphone application for symptom monitoring in early psychosis care It reported high compliance with the app usage, with a significant portion of participants regularly completing both daily and weekly surveys. Symptom Assessment: The results supported the use of the smartphone app for assessing symptoms comparable to clinician-rated interviews, particularly for positive and depression/anxiety symptoms The assessments provided by the app showed strong correlations with the gold-standard assessments, reinforcing the potential utility of mobile health technology in clinical settings. Engagement and Retention: The study highlighted that patients in early psychosis treatment were willing to engage with and maintain use of mobile technology as part of their care, indicating a positive reception and potential for broader application. | Control Group: The study did not include a control group, which limits the ability to fully evaluate the impact of the smartphone intervention against standard treatment methods. Generalizability: The participants were predominantly from a specific clinical program (UC Davis Early Psychosis Program), which may not represent the broader population of individuals with early psychosis. Technology Bias: The study might have a selection bias, as it only included participants amenable to using technology, which might not reflect the wider early psychosis patient population. Data Interpretation: Some data interpretations, particularly regarding negative symptoms, were less conclusive The app-based assessments for negative symptoms did not show a significant correlation with clinician ratings, indicating limitations in capturing these symptoms through self-reported methods. |
|  | Detecting Bipolar Depression From Geographic Location Data | Palmius, N.; Tsanas, A.; Saunders, K. E. A.; Bilderbeck, A. C.; Geddes, J. R.; Goodwin, G. M.; De Vos, M. | Questionnaire Score Estimation: Using geolocation-derived features from bipolar disorder (BD) participants demonstrated a mean absolute error rate of 3.73 in estimating questionnaire scores, which indicates the effectiveness of using these features for assessing depression levels.  Depression Detection Performance: The optimal performance for detecting depression achieved a median F1 score of 0.857 with an interquartile range (IQR) of ±0.022 The classification accuracy was 0.849 (±0.016 IQR), with sensitivity at 0.839 (±0.014 IQR) and specificity at 0.872 (±0.047 IQR) This demonstrates a strong link between geographic movements and depressive states in bipolar disorder, showing that changes in movement patterns can effectively indicate depressive episodes. | Effective Detection: The study demonstrated that geographic movement patterns, as measured through mobile device data, are strongly linked to depressive states in individuals with bipolar disorder. High Classification Accuracy: The methods used achieved high classification accuracy, sensitivity, and specificity in identifying depressive episodes using geolocation features. Potential for Monitoring: The findings suggest that passive data streams from mobile devices, such as geolocation, could serve as a valuable tool for monitoring mood states in bipolar disorder, potentially aiding in early intervention and management of depression. | Data Gaps and Compliance: There were significant gaps in data due to participants not always carrying the mobile device or due to technical issues This affected the continuity and completeness of the geolocation data. Generalizability: The study's findings may not be generalizable to all populations with bipolar disorder, as the sample was relatively small and specific to a community setting around Oxford. Dependence on Device Performance: The accuracy of the geolocation data was highly dependent on the performance of the mobile devices used, which might vary widely among different devices and conditions. |
|  | Monitoring Changes in Depression Severity Using Wearable and Mobile Sensors | Pedrelli, Paola; Fedor, Szymon; Ghandeharioun, Asma; Howe, Esther; Ionescu, Dawn F; Bhathena, Darian; Fisher, Lauren B; Cusin, Christina; Nyer, Maren; Yeung, Albert; Sangermano, Lisa; Mischoulon, David; Alpert, Jonathan E; Picard, Rosaline W | Adherence to Sensor Use: Participants wore the right and left wrist sensors 92% and 94% of the time, respectively This high adherence indicates good compliance with the wearable component of the study.  Machine Learning Model Performance:  The models developed to estimate depressive symptom severity from smartphone and wearable sensor data showed moderate to high correlations with clinician-rated HDRS scores The correlations ranged from 0.46 to 0.7. The mean absolute error (MAE) of the models' estimates of HDRS scores ranged between approximately 3.88 and 4.74, suggesting moderate accuracy in predicting depression severity. The model that included only smartphone data features performed the best in one of the evaluation scenarios, demonstrating the lowest MAE. Predictive Features:  The ten most predictive features identified by the models were related to mobile phone engagement, activity level, skin conductance, and heart rate variability These features were significant in estimating the severity of depressive symptoms. | Utilizing smartphones and wrist sensors to monitor patients with Major Depressive Disorder (MDD) after undergoing a clinician-rated Hamilton Depression Rating Scale (HDRS) assessment appears viable and could offer insights into fluctuations in depressive symptom severity. | The study was limited by a small sample size and participants who exhibited minimal variability in depressive symptoms throughout the study period For instance, the within-user HDRS scores showed an average standard deviation of 3.7, and the average decrease in HDRS scores from baseline to the final assessment was 15% This raises uncertainty about the generalizability of the model's performance to patients with more fluctuating depressive symptoms. |
|  | Behavioral Indicators on a Mobile Sensing Platform Predict Clinically Validated Psychiatric Symptoms of Mood and Anxiety Disorders | Place, Skyler; Blanch-Hartigan, Danielle; Rubin, Channah; Gorrostieta, Cristina; Mead, Caroline; Kane, John; Marx, Brian P; Feast, Joshua; Deckersbach, Thilo; Alex “Sandy” Pentland; Nierenberg, Andrew; Azarbayejani, Ali | Participant Characteristics: A total of 73 participants completed the 12-week study, including 49 males (67%) and 24 females (33%) The participants included 48% non-Hispanic white and 33% veterans​​.  Models and Model Fit:  Four symptom targets were modeled: depressed mood, fatigue, interest in activities, and social connectedness. The models showed cross-validated areas under the curve (AUCs) for predicting these symptoms as follows: Depressed mood: AUC = 0.74 Fatigue: AUC = 0.56 Interest in activities: AUC = 0.75 Social connectedness: AUC = 0.83​​. Acceptability and Feasibility:  Adherence to the study protocol was high, with 96% of participants completing at least one audio diary per week. The mobile app was rated easy to use (Mean = 4.05, SD = 1.14). Interest in future use of the app was moderate (Mean = 3.59, SD = 1.27). Comfort with sharing individual data varied, with participants expressing more comfort in sharing with physicians (Mean = 3.08, SD = 1.22), mental health providers (Mean = 3.25, SD = 1.39), and medical researchers (Mean = 3.03, SD = 1.36) compared to lower comfort levels with sharing data with insurance providers and friends​​. | The study found that behavioral indicators collected through a mobile sensing platform could predict clinically assessed symptoms of depression and PTSD. The models used were parsimonious, objective, and actionable They utilized passively collected digital trace data and extracted vocal features of tonality and speaking style. Adding self-reported predictors to the models did not significantly increase model performance, highlighting the effectiveness of the behavioral indicators alone. These behavioral indicators are clinically meaningful and can capture levels of impairment that are relevant for clinical assessments and interventions​​. | There was a high rate of data loss due to technical issues (e.g., lost phones), which affected the study's participant retention. |
|  | A comparison of passive and active estimates of sleep in a cohort with schizophrenia | Staples, Patrick; Torous, John; Barnett, Ian; Carlson, Kevin; Sandoval, Luis; Keshavan, Matcheri; Onnela, Jukka Pekka | Active data from Ecological Momentary Assessments (EMAs) and passive data from smartphone sensors were used to estimate sleep quality and patterns. The in-clinic Pittsburgh Sleep Questionnaire Inventory (PSQI) assessments were compared to smartphone-based estimates The cross-validated simple linear regression model showed that EMAs and passive data classified 85% of subjects (11 out of 13) as exhibiting high or low sleep quality, aligning with in-clinic assessments. The mean average error between predictions from smartphone data and actual PSQI scores was 0.75, indicating a strong prediction capability This suggests that smartphone-based monitoring can effectively estimate sleep quality as assessed in a clinical setting. Smartphone accelerometer data used to infer sleep duration was moderately correlated with subject self-assessment of sleep duration, with a correlation coefficient (r) of 0.69 and a 95% confidence interval of 0.23–0.90. For future PSQI scores, predicted by using smartphone EMAs and passive data, the mean absolute error was 1.9 on a scale from 0 to 14, suggesting that smartphone-based assessments can provide a reasonably accurate forecast of sleep quality. | The study demonstrated that smartphone-based monitoring, using both passive data (such as accelerometer readings) and active data (Ecological Momentary Assessments - EMAs), is feasible and effective for assessing sleep in patients with schizophrenia. The results indicated that smartphone EMAs and passive sensor data could classify sleep quality with high accuracy when compared to traditional in-clinic assessments using the Pittsburgh Sleep Questionnaire Inventory (PSQI). The correlation between smartphone-based estimates and in-clinic PSQI scores showed that smartphone monitoring could serve as a reliable tool for evaluating sleep in this population. Smartphone data predicted clinical PSQI scores with a mean average error of 0.75, which was considered quite accurate, suggesting that smartphone tools can be used effectively in long-term monitoring and management of sleep quality in schizophrenia. The predictive model for future PSQI scores based on smartphone data indicated a mean average error of 1.9 on a scale of 0-14, demonstrating the potential for these tools to forecast future sleep quality metrics. | The absence of validation against more definitive sleep monitoring methods like polysomnography was noted as a limitation, which might affect the accuracy of the sleep duration estimates from smartphone data. A significant amount of accelerometer data was missing, potentially due to factors like phones being turned off, which could limit the robustness of the sleep estimates. The study’s small sample size and observational design could affect the generalizability of the findings and the statistical power to detect more nuanced associations. |
|  | Dynamic Bidirectional Associations Between Global Positioning System Mobility and Ecological Momentary Assessment of Mood Symptoms in Mood Disorders: Prospective Cohort Study | Ting‑Yi Lee; Ching‑Hsuan Chen; I‑Ming Chen; Hsi‑Chung Chen; Chih‑Min Liu; Shu‑I Wu; Chuhsing Kate Hsiao; Po‑Hsiu Kuo | Polyserial correlation analysis showed significant associations between certain GPS mobility features and EMA mood scores, with correlation coefficients ranging from 0.24 to 0.41 (P<.05). In the generalized estimating equation models, increased time spent at home and reduced location variance were significantly associated with higher depressive symptom scores (β=0.18, P=.02; β=−0.15, P=.04, respectively), while greater total distance traveled was associated with lower depressive symptoms (β=−0.20, P=.01). For manic symptoms, greater location variance and higher number of locations visited were positively associated with symptom scores (β=0.22, P=.03; β=0.19, P=.04, respectively). | Reduced mobility, indicated by increased time spent at home and lower location variance, was associated with worsening depressive symptoms, while greater mobility (total distance traveled) was linked to lower depression scores. In contrast, manic symptoms were associated with greater location variance and visiting more locations. These results suggest that GPS-derived mobility features can capture meaningful fluctuations in mood states among individuals with mood disorders. | Small sample size with moderate statistical power (0.70) limits representativeness; absence of Apple iOS data may introduce selection bias given high iOS market share in Taiwan; missing data due to hospitalization prevented EMA and GPS capture for some participants; potential recall bias from flexible EMA completion times, though high completion rates and strong intraclass correlations suggest stable reporting; approximately 16% dropout within the first two weeks. |
|  | Associations among smartphone app-based measurements of mood, sleep and activity in bipolar disorder | Tseng, Yu-Ching; Lin, Esther Ching-Lan; Wu, Chung Hsien; Huang, Huei-Lin; Chen, Po See | Smartphone-Based Measurements of Mood, Sleep, and Activity:  The mean daily mood recorded was 3.31 (on a scale where higher scores indicate better mood). The average sleep duration was 8.79 hours per night. The average daily distance of movement was 5,950 meters. Correlations and Associations:  The daily mood scores collected via the app showed significant positive correlations with the scores on mood-related sub-items of various clinical scales. Sleep duration from the app correlated significantly with the sleep duration reported in the Pittsburgh Sleep Quality Index (PSQI). No significant correlations were found between the total daily distance of movement and any of the clinical scale scores. Analysis of Associations Over Time:  In daily comparisons, significant positive correlations were found within individual variables but not between different variables. Over weeks, daily mood positively correlated with total daily distance of movement in the subsequent week Sleep duration was positively correlated with daily mood and total daily distance of movement in the subsequent week. Over months, similar significant inter-variable correlations were observed, indicating a positive association between these variables over longer periods. | Smartphone App Validity: The smartphone app used in the study provided valid measurements of mood and sleep, which were significantly correlated with clinical scales and the PSQI, respectively. Inter-variable Associations: Significant positive correlations were observed not only within each variable (mood, sleep, and activity) but also between these variables over weekly and monthly time frames For instance, better mood was associated with increased activity and sleep duration in subsequent periods. Long-Term Monitoring: The app demonstrated potential for long-term monitoring of BD symptoms, suggesting that interventions targeting multiple aspects of patient behavior could be effective. | Population Stability: The study included only outpatients with relatively stable symptoms, which might limit the generalizability of the findings to patients with more severe symptoms of BD or those who are inpatients. Irregular App Use: Many subjects did not use the app regularly or continuously, which could impact the data's reliability and the overall findings Inconsistent data collection might affect the strength and validity of the observed associations. Technical Issues with Data Collection: Difficulties in collecting sufficient GPS data due to lack of internet access or the environment (e.g., inside buildings) potentially affected the activity data's accuracy. Potential for Bias in Statistical Analysis: The analysis method used might lead to type I errors |
|  | Characterizing the clinical relevance of digital phenotyping data quality with applications to a cohort with schizophrenia | Torous, John; Staples, Patrick; Barnett, Ian; Sandoval, Luis R; Keshavan, Matcheri; Onnela, Jukka-Pekka | Participation and Data Collection: Out of 16 subjects in a state mental health clinic, 5 experienced a relapse during the study, which was defined by hospitalization for psychiatric reasons, or an increase in the level of care or medications related to psychiatric symptoms The subjects used the Beiwe smartphone app, which collected both active and passive data, for up to 3 months.  Data Quality Measures and Their Relationship with Survey Responses: Various measures of data quality, such as the coverage of passive data like accelerometer and GPS data, as well as the timing and completion of surveys, were analyzed for their association with future survey scores This analysis was important to determine if the quality of data collected could predict future clinical outcomes.  Statistical Analysis Findings:  A significant correlation was found between measures of data quality and future survey responses For example, decreased accelerometer data coverage was weakly associated with higher survey scores, which typically indicated worse clinical outcomes. Faster survey viewing times and slower completion rates were associated with higher (worse) survey scores, especially in questions probing anxiety... | Clinical Relevance of Data Quality: The study demonstrated that data quality metrics from smartphone-based digital phenotyping, including empirical coverage of collected accelerometer and GPS data, as well as survey timing and completion metrics, are significantly associated with future survey scores for various symptom domains in schizophrenia.  Predictive Potential of Metadata: The metadata, which includes details like the timing of survey prompts and the actual times of survey completion, was found to contain potentially clinically relevant information that could predict future clinical outcomes This finding suggests that the way patients interact with the digital phenotyping app can provide insights into their mental state and possible future changes in their condition.  Association with Clinical Symptoms: Measures of data quality were found to correlate with the severity of clinical symptoms For example, lower accelerometer data coverage and faster survey viewing times were associated with higher survey scores, indicating worse outcomes These associations were particularly noted in symptom domains related to anxiety and anhedonia. | Small Sample Size: The study involved only 16 subjects, which is relatively small and might limit the generalizability of the findings A larger sample would provide more robust data and help to confirm the patterns observed.  Variability in Smartphone Usage: The variability in how subjects used their smartphones could influence the data collected Factors such as different smartphone models, operating systems, and individual usage habits might affect the consistency and reliability of the data.  Passive Data Collection Issues: The study noted issues with passive data collection, such as the incomplete coverage of GPS and accelerometer data This incomplete data collection could be due to technical limitations, such as the inability to continuously sample GPS without draining the phone's battery or the phone being in a location that obstructs GPS signal.  Potential for Technological Confounders: Differences in smartphone hardware and software between subjects could act as confounders... |
|  | Predicting Mood Disturbance Severity with Mobile Phone Keystroke Metadata: A BiAffect Digital Phenotyping Study | Zulueta, John; Piscitello, Andrea; Rasic, Mladen; Easter, Rebecca; Babu, Pallavi; Langenecker, Scott A; McInnis, Melvin; Ajilore, Olusola; Nelson, Peter C; Ryan, Kelly; Leow, Alex | - Sample size: 99 - Fitbit devices: 41 Charge 3, 58 Charge 4  Data completion:  – Wearables: 91% (SD 22%) week 1  – Smartphones: 82% (SD 32%) week 1  – Higher completion with study phone vs own phone (91% vs 77%, *P*<.001)  Symptom–biomarker associations:  – Negative symptoms (BNSS & PANSS-N): linked with the greatest number of digital measures (8/12).  – Experiential deficits (anhedonia, avolition): associated with less distance travelled, more time awake in bed.  – Asociality: associated with fewer messages sent (sociability marker).  – Expressive deficits (blunted affect, alogia): negatively associated with steps taken. – Alogia: also linked with sleep features (total sleep, light sleep, REM sleep). – PANSS-positive & SOFAS: each associated with ≥3 digital measures.  – Cognition & cognitive/disorganization symptoms: associated with 1–2 measures.  – Heart rate (asleep): showed the broadest clinical associations (higher HR linked to higher PANSS total and factors, BNSS avolition/asociality, poorer functioning, and lower cognition).  – Time awake in bed & distance travelled: consistently associated with negative symptoms (anhedonia, avolition, functioning).  – Screen time: not significantly associated with any clinical measure. | Passive digital markers captured clinically relevant patterns across multiple domains.  Negative symptoms showed the strongest and most consistent associations: - Experiential deficits (anhedonia, avolition) linked to reduced mobility and increased time awake in bed. - Asociality associated with fewer messages sent. - Expressive deficits (blunted affect, alogia) linked to lower step count and altered sleep architecture (longer light and REM sleep).  HR during sleep stood out as the most consistent biomarker, linked with illness severity, functioning, and cognition. Authors suggest it may serve as a physiological indicator of psychosis burden.  Sleep efficiency was inversely related to avolition and cognitive/disorganization symptoms.  Screen time was not associated with any clinical domain.  Findings support the utility of wrist wearables and smartphones for unobtrusive, longitudinal monitoring of psychiatric symptoms and functioning. | Young outpatients, mildly ill (PANSS-total score: 51), with short duration of illness (2.6 years), limited generalizability to older or more severe psychosis.  Cross-sectional (week 1 only); no causal inference.  All on psychotropics, possible effects on HR/sleep.  Incentives may have boosted compliance.  No multiple-testing correction (exploratory). |
|  | An Observational Pilot Study using a Digital Phenotyping Approach in Patients with Major Depressive Disorder Treated with Trazodone | Čermák, Jan; Pietrucha, Slavomír; Nawka, Alexander; Lipone, Paola; Ruggieri, Alessandro; Bonelli, Annalisa; Comandini, Alessandro; Cattaneo, Agnese | No significant correlations were discovered between total key counts and subjects' age (S=139.16, P=.68) or education levels (S=144.41, P=.60).  In predicting depression symptoms, likelihood ratio testing comparing the null model containing only the subject-level random effect to the full model indicated that the full model exhibited superior fit (χ²7=17.6, P=.01) The marginal R² was 0.41, while the conditional R² was .63 Factors positively correlated with HDRS scores included accelerometer displacement (P=.002), average interkey delay (P=.02), session count (P=.003), and autocorrect rate (P=.004).  Regarding the prediction of hypomania or mania symptoms, a multiple linear regression model explained 34% of the variance in the natural logarithm of YMRS scores (multiple R²=.34, F7,56=4.08, root mean square error=.66, P=.001) Accelerometer displacement (P=.003) demonstrated a positive correlation with YMRS scores, whereas the backspace rate (P=.01) exhibited a negative correlation. | Changes in mobile phone usage seem to be associated with mood states in individuals with bipolar disorder Developing models based on these changes suggests that using keyboard metadata collected passively could be a viable method for detecting and tracking mood disturbances. | Limitations of this study include its relatively small sample size compared to the complexity of the model, as well as the sample's characteristics, which may not be representative of the general population, primarily comprising mostly women with a high frequency of episodes Additionally, the study's constraint of participants using study-issued phones restricts its generalizability A larger study involving participants using their own phones is necessary to validate these findings and potentially enable the development of more sophisticated models with improved prediction accuracy and reliability.  Furthermore, the prediction of mania scores was less accurate compared to depression scores, likely due to fewer predictors and the sporadic nature of mania episodes This limitation is particularly significant given the clinical implications of predicting acute changes in mania, as individuals experiencing mania may be less inclined to seek treatment... |
|  | Utility of Digital Phenotyping Based on Wrist Wearables and Smartphones in Psychosis: Observational Study | [Zixu Yang](https://pubmed.ncbi.nlm.nih.gov/?term=%22Yang%20Z%22%5BAuthor%5D), [Creighton Heaukulani](https://pubmed.ncbi.nlm.nih.gov/?term=%22Heaukulani%20C%22%5BAuthor%5D), [Amelia Sim](https://pubmed.ncbi.nlm.nih.gov/?term=%22Sim%20A%22%5BAuthor%5D), [Thisum Buddhika](https://pubmed.ncbi.nlm.nih.gov/?term=%22Buddhika%20T%22%5BAuthor%5D), [Nur Amirah Abdul Rashid](https://pubmed.ncbi.nlm.nih.gov/?term=%22Abdul%20Rashid%20NA%22%5BAuthor%5D), [Xuancong Wang](https://pubmed.ncbi.nlm.nih.gov/?term=%22Wang%20X%22%5BAuthor%5D), [Shushan Zheng](https://pubmed.ncbi.nlm.nih.gov/?term=%22Zheng%20S%22%5BAuthor%5D), [Yue Feng Quek](https://pubmed.ncbi.nlm.nih.gov/?term=%22Quek%20YF%22%5BAuthor%5D), [Sutapa Basu](https://pubmed.ncbi.nlm.nih.gov/?term=%22Basu%20S%22%5BAuthor%5D), [Kok Wei Lee](https://pubmed.ncbi.nlm.nih.gov/?term=%22Lee%20KW%22%5BAuthor%5D), [Charmaine Tang](https://pubmed.ncbi.nlm.nih.gov/?term=%22Tang%20C%22%5BAuthor%5D), [Swapna Verma](https://pubmed.ncbi.nlm.nih.gov/?term=%22Verma%20S%22%5BAuthor%5D), [Robert J T Morris](https://pubmed.ncbi.nlm.nih.gov/?term=%22Morris%20RJT%22%5BAuthor%5D), [Jimmy Lee](https://pubmed.ncbi.nlm.nih.gov/?term=%22Lee%20J%22%5BAuthor%5D) | Device activation and deactivation occurred as planned at Baseline and Follow-up visits for all 11 patients (100.0%).  The majority of dose adjustments, observed in eight patients (72.7%), followed standard titrations consistent with the study protocol, increasing the dose of trazodone OAD No dose adjustments were made during the study for two patients, who remained on a 75.0 mg/day dose, below the therapeutic level.  The median dosage of trazodone OAD monotherapy at Day 0 was 75.0 (range: 75.0–150.0) mg/day; nine of the 11 patients (81.8%) started at 75.0 mg/day, while two patients (18.2%) started at 150 mg/day Treatment changes occurred for eight patients (80.0%) during the study, all at scheduled visits Maximum doses reached were 300.0 mg/day for two patients, 225.0 mg/day for one patient, 150.0 mg/day for six patients, and two patients remained at 75.0 mg/day for the duration.  Primary Endpoint: Distance traveled: Mean (SD) daily distance traveled per day during Week 1 was 4949.6 (4098.86) m... | Preliminary findings indicate the effectiveness of trazodone OAD monotherapy in improving depressive symptoms and sleep quality in patients with MDD Digital phenotyping, along with traditional clinical evaluation, has the potential to engage patients and provide valuable insights into treatment response and tolerability with minimal patient burden Further investigation in larger, controlled clinical trials is warranted. | The limited size of the study population constrains the extent to which conclusions can be made regarding the relationship between the digital phenotyping method and conventional clinical assessment. |
